# Computational elucidation of the effects induced by music making

**DOI:** 10.1371/journal.pone.0213247

**Published:** 2019-03-07

**Authors:** Billie Sandak, Shai Cohen, Avi Gilboa, David Harel

**Affiliations:** 1 Department of Computer Science and Applied Mathematics, Faculty of Mathematics and Computer Science, The Weizmann Institute of Science, Rehovot, Israel; 2 Department of Music, The Faculty of Humanities, Bar-Ilan University, Ramat-Gan, Israel; Griffith University, AUSTRALIA

## Abstract

Music making, in the form of free improvisations, is a common technique in music therapy, used to express one’s feelings or ideas in the non-verbal language of music. In the broader sense, arts therapies, and music therapy in particular, are used to induce therapeutic and psychosocial effects, and to help mitigate symptoms in serious and chronic diseases. They are also used to empower the wellbeing and quality of life for both healthy individuals and patients. However, much research is still required to understand how music-based and arts-based approaches work, and to eventually enhance their effectivity. The clinical setting employing the arts constitutes a rich dynamic environment of occurrences that is difficult to capture, being driven by complex, simultaneous, and interwoven behavioral processes. Our computational paradigm is designed to allow substantial barriers in the arts-based fields to be overcome by enabling the rigorous and quantitative tracking, analyzing and documenting of the underlying dynamic processes. Here we expand the method for the music modality and apply it in a proof of principle experimentation to study expressive behavioral effects of diverse musical improvisation tasks on individuals and collectives. We have obtained statistically significant results that include empirical expressive patterns of feelings, as well as proficiency, gender and age behavioral differences, which point to variation factors of these categorized collectives in music making. Our results also suggest that males are more exploratory than females (e.g., they exhibit a larger range of octaves and intensity) and that the older people express musical characterized negativity more than younger ones (e.g., exhibiting larger note clusters and more chromatic transitions). We discuss implications of these findings to music therapy, such as behavioral diversity causality in treatment, as well as future scientific and clinical applications of the methodology.

## Introduction

Musical improvisation making is a common technique in music therapy [1,2], and implies that playing can be conducted not only by people who were taught to play or to read notes, but by any person who can intuitively use an instrument to express an idea or a feeling, using the non-verbal language of music. Music therapy, as well as other arts-based approaches and interventions, is used in diverse populations and age groups to help alleviate symptoms and induce therapeutic and psychosocial effects in a wide variety of serious and chronic conditions, illnesses, mental disorders, disabilities, etc. For example, music therapy has been shown to help mitigate symptoms such as pain, stiffness, fatigue, depression, stress, breathlessness and anxiety are mitigated for cancer [3,4], Parkinson [5], coronary [6] dementia [7] and mental [8, 9] patients; young or old [10–12]. For a either patient or a healthy individual, the engagement with music also enhances one’s well-being and quality of life [13–15], and is also useful research and practice in the social sciences, aimed at understanding and empowering individuals, groups and society [16,17]. The benefits of music-based approaches are also manifested in psychophysiological measurements; e.g., reduction in heart-rate, blood pressure and cortisol levels, and increase in melatonin levels [18–21]. Music-based therapy has been employed clinically for centuries [22], in hospitals, schools, community centers, etc., and is now recognized as a discipline [23]. Nevertheless, much research is required to reveal the underlying expressive behavioral mechanisms by which music-based approaches operate, also as a non-pharmacological treatment, and to help enhance their effectiveness [24,25].

Improvisation is one of the powerful tools used in music therapy, and it achieves goals beyond the apparent aesthetic and social enjoyment. Directing patients or clients to improvise freely with no aesthetically pre-defined constraints, enables them to develop their creativity and expressivity. In addition, according to analytic music therapy [26–28], improvisations are used to interpret subconscious processes, that is, words and symbolic music improvisation are used as means to explore the clients’ inner life and facilitating growth. As such, it is natural for the music therapist to suggest to the client a title to improvise on, such as a certain topic, issue, or feeling [26,27,29–33]. These titles are not restricted to any theoretical schemata (such as a specific set of basic emotions) but are, on the contrary, tailored to suit the client’s troubling issues at hand. Titles can be abstract (e.g., “grand,” “tiny,” to encourage client’s creative exploration), personal (e.g., “father,” “home,” to encourage client’s examination of his or her past and present relationships), descriptive (e.g., “climbing a mountain,” to encourage visual imagery while improvising), and many other possibilities and/or combinations thereof.

A typical clinical setting employing the arts consists of the creation work itself, such as the musical work, the therapist and the patient. For example, in musical work, these include the beginning and end of a played musical note, the pitch, intensity, clustering of notes played in parallel, tempo and instrument choices. In addition to the dynamic processes of the artistic construction work itself, there is the social interaction of the patient and therapist, which involves their bodily and verbal and non-verbal communication. These complex, simultaneous, and interwoven behavioral processes are often considered hard to capture and track by human observers. As a consequence, they are usually perceived and interpreted subjectively, and are described verbally, thus affecting the subsequent analyses and understanding.

Methods were developed to analyze musical improvisations, such as phenomenological analysis [34,35] and graphical analysis [36,37]. However, these methods rely on subjective accounts and interpretations of the analyzers. Other, more quantitative, micro-analytical methods [38] rely on objective counting of specific occurrences, but they are usually conducted manually without the assistance of computers. Past attempts to use computation to analyze music making were found to be limited ad-hoc implementations; the recording of some particular parameters that were based on pre-determined hypotheses was carried out in [39], and some featured tools were demonstrated on two single case studies [40]. Music-based approaches are carried out along the continuum of ‘music as therapy’ ↔ ‘music in therapy’ [29]. In the latter notion, the therapist intervenes in trying to initiate changes, i.e., connects and acts upon psychological dimensions of the musical experience, whereas in former, ‘music as therapy’, it is assumed that the music making is the therapeutic process itself [41] and thus the musical work is the focus of attention and is what we investigate here.

In the paper, we expand the broad computational paradigm *(****CP****)* we previously developed [42], which allows substantial barriers in the arts fields to be overcome, and apply it to the music modality in real-world proof-of-principle experimentation. The technology was designed to capture the creation and interaction processes, and then to empirically elucidate and analyze the underlying expressive and social behaviors. This includes examining individual and collective parameters and measurements for performance analysis and comparisons. All these allow our technology to be used in investigations along the ‘music as therapy’ ↔ ‘music in therapy’ continuum, providing novel insights and empirical probing abilities, also in order to discover how arts-based approaches work, and eventually, to ameliorate their use.

The CP captures and decodes emergent behaviors; i.e., arising properties and patterns of the behavioral processes, and includes: *(i)* measuring and calculating exact time durations of occurrences within the music session; e.g., net idle time in which the patient/client is not engaged in musical activity or pressing a key, the actual start time within, net playing time and concurrent playing time, i.e., play time obtained from notes (keys) pressed in parallel; *(ii)* tracking note use per time and per presses; e.g., net number of notes used, total number of notes pressed, their time durations and density, and their cluster formations; *(iii)* capturing and analyzing preference profile of octave use and notes’ intensity in the music making process; e.g., whether it is carried out in confined pitch (registers) and intensity levels (musical dynamics); *(iv*) profiling pitch classes; that is, the note use distribution collapsed onto an octave (C, C#, D,…, A#, B pitch), as well as chromatic preference (say, note color on a piano keyboard, i.e., black and white keys); *(v)* calculating transitions; e.g., crescendo, diminuendo, accelerando, ritardando and chromatic (for example, black to white, white to white, etc.).

The musical work focus is the first step prior to the exploration of the contribution of therapist-patient interaction, and hence our study focuses on music making and the expressive dynamics therein. Four free musical improvisation tasks were given to 108 participants, who were asked to musically express the titles of “positive feeling,” “negative feeling,” “beautiful,” and “ugly” on a piano keyboard, which provides tremendous opportunities for expression (see Experimental design). These titles were chosen to account for the ecological validity of the music therapy environment, in which different titles could be presented to the client as triggers for his or her improvisations. The first two titles adhere to the general theoretical concept that feelings can be differentiated according to their valence (e.g., positive vs. negative; see Russell’s circumplex model; [43]). However, no specific emotions were dictated so as to enable different interpretations. The other two titles, still adhering to the general division between positive and negative valence, were tailored to be more abstract and open to personal projections (i.e., what beautiful/ugly is connected to in the client’s life), as would be typical in an analytical music therapy session [26,27,33]. Note that although there might seem to be dependence between “negative feeling” and “ugly” (which is usually perceived negatively) and between “positive feeling” and “beautiful” (which is usually perceived positively), the participants’ improvisations and their analysis will determine whether such dependence exists in the context of musical expression.

**Fig 1:**
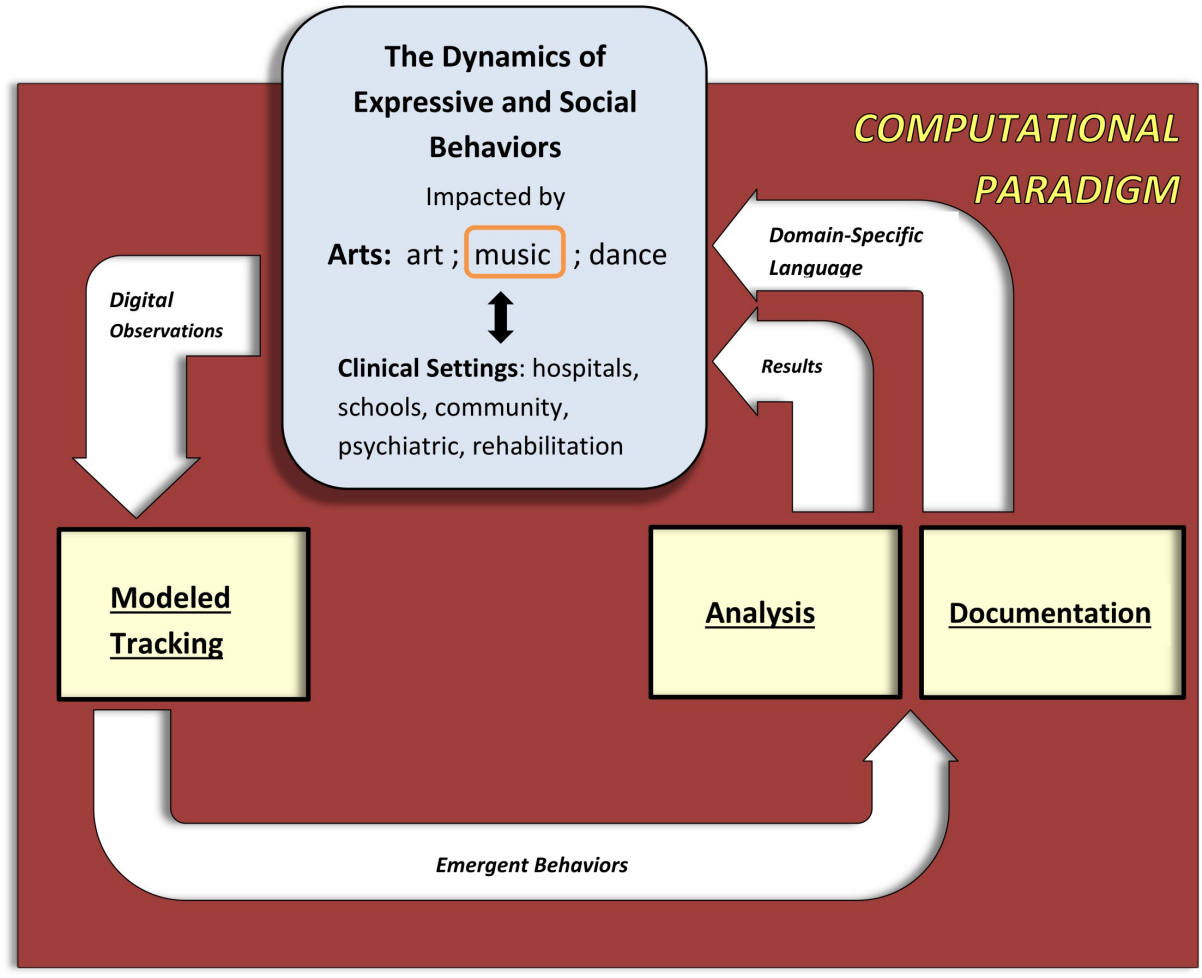
The computational paradigm and its constituting components. Digital observations of the system under study, e.g. music making, are fed into the ***Modeled Tracking*** module, which captures the occurring events to yield emergent behaviors. These are input to the ***Analysis*** and ***Documentation*** modules, the first of these outputs empirical insights into the field of study, e.g., music therapy, and the second transforms the behavioral dynamics to amenable description.

We analyzed the dynamics of the emergent behavioral processes in response to these improvisation tasks, according to the parameters described above (see also Fig 1 and Materials and methods section), for individuals and collectives at multiple levels; i.e., for single and multiple musical tasks, obtaining significant task-based and demographic-based differences, as described in the following sections, as well as their implications to music therapy. We also discuss the CP’s further potential contribution to scientific and clinical research, enabling one to carry out exploratory, hypotheses-testing and -generating and knowledge discovery investigations, which are empirically based.

## Materials and methods

The study reported upon in this paper is a proof of principle application of our CP to empirically unraveling the effects of music making. We refer the reader to [42] for a more detailed description of the methodology’s architecture and modeling considerations for the various arts modalities. Here we provide a briefer description of the development of the method for the music modality.

**Fig 2:**
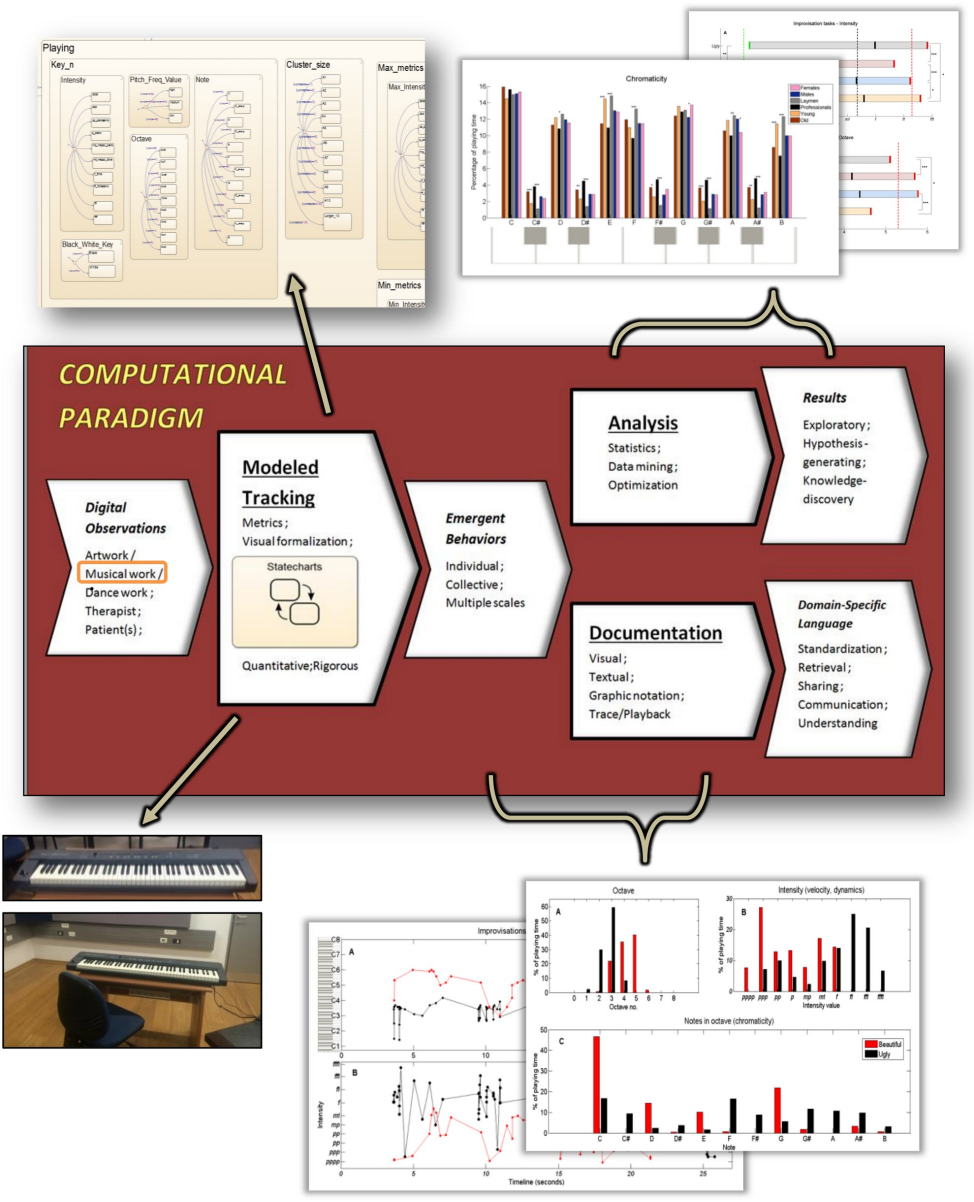
The paradigm applied to musical work. An illustration of the technology use in this study: Tracking, analyzing and documenting dynamic processes in music making.

As depicted in Figs 1 and 2, our CP suite consists of: (i) the ***Modeled Tracking*** module, responsible for capturing the dynamics of the system modeled, via digitized input, since the naked human eye cannot rigorously and objectively capture the observed behavior of the system studied. In this case, the system studied is musical work, input by a digital piano keyboard (see bottom left side of Fig 2). This module hosts the system’s model, which is Statecharts-based [44] (see top left side of Fig 2 and the following ‘Music room modeling’ subsection); (ii) the ***Analysis*** module, responsible for investigating decoded emerging individual and collective behaviors of the system modeled in response to music making. In this module we employ mathematical, computational, statistical and algorithmic tools to investigate the data output obtained by the modeled tracking module, as dictated by the study’s aims (see top right side of Fig 2 and the subsequent ‘Experimental design’ subsection); (iii) the ***Documentation*** module, which transforms the expressive emergent behaviors into a format amenable to easy contemplation. This is done by combining textual and graphical reports to convey the properties of the dynamics of the music-making processes (see bottom right side of Fig 2 and S1–S3 Figs).

### Music room modeling

Three major entities comprise the music room: the creation work (that is, the musical work itself), the patient and the therapist. These components and their interactions constitute a dynamic system that continuously reacts to internal and external stimuli; i.e., what has been termed a *reactive system* [45]. Within this system, the musical creation/construction work itself, which is the center of focus in this study, is considered a reactive sub-system, also driven by events. These include choosing a musical instrument, such as a piano keyboard, starting to play a musical note, and stopping it. The events transfer the system from state to state, for example, from ’instruments being selected’ to ’playing’. The system will enter the state of ’idle’ when the creator/client/patient/professional/layman stops being active; e.g., he/she starts to think of the next note or to take a rest. In a clinical setting, the idle state will often be reached as a result of the therapist asking the client to stop playing or simply when the improvisation ends. We base our modeling method on Statecharts [44] and its underlying execution and analysis tools [46–48]. Statecharts are a visual formalism [49], which enriches the basic state/event modeling approach with means for describing *hierarchy* (nested states) and multi-level transitions, as well as *orthogonality* (concurrent states), and more. See S4 Fig for the top view of the Statechart modeling of the music room. The model for a music session includes the three states **Music_Work**, **Client** and **Music_Therapist**, which are concurrent and are enclosed by their parent super-state, **MusicRoom_SessionOn**. The musical work is the center of creation in the music room, and can exist without the need for a clinical setting. We concentrate on it in the improvisation study carried out here. The **Music_Work** subsystem state is decomposed into its exclusive substates, **Idle**, **Selecting**, and **Playing**, with the latter state to further include the complex and rich dynamics therein, e.g., the orthogonal states **Timbre**, **Duration, Tempo, Cluster_size, Key_n, Max_metrics** and **Min_metrics** states. See S5 Fig. Each of these states is further described by its substates. For example, the **Duration** state tracks the duration of the current improvisation, e.g., **long**, **extremely_short** or other improvisation lengths, whereas **Cluster_size** is responsible for tracking the configuration size of current pressed cluster of keys, i.e., 1, 2, … 10, or above 10. The **Max_metrics** and **Min_metrics** states track current maximum and minimum values of the intensity, octave number and cluster size. Each key (state **Key_n**) is tracked for its intensity, the note produced, the octave it is in, its pitch level and whether the key is black or white (when playing the piano **keyboard**, a state in **Timbre**). Since the Statecharts language has a formal executable syntax and semantics, both textual and graphical terms have precise dynamic meaning, so that the model can be analyzed for dynamic properties and simulated directly, or translated into fully executable code.

### Experimental design

#### Setup

The musical instrument the participants used was a Roland A-30-MIDI (Musical Instrument Digital Interface) piano keyboard controller (see S6A Fig). We employed the MIDI protocol [50,51] for digital data collection; that is, pressing a key generates its time stamp, note number and velocity (intensity or pressure exerted on the key). The velocity values range from 0 to 127 are categorized from level *pppp* to *ffff* according to the common categorization of raw MIDI velocity data [51]. The improvisation data was recorded using Cubase9 [52] and was transformed by Max/MSP [53] to output script files. These were subsequently “read into” the Statecharts model and analyzed by our methodology.

#### Subjects

The study involved 108 healthy/normal-hearing participants, 54 male and 54 female, with an age mean of 33.1 (SEM = 1.3), age range of 18 to 77, and age median of 28. Half of the total number of participants had formal musical studies/training or playing experience (professionals), and the other half either had none or had some childhood playing training (lay persons or laymen). All participants came from similar cultural and educational backgrounds—campus students, faculty, administration and visitors. The participants were recruited by ads hung around the campus or by directly approaching them (135 were approached). No participants dropped out after consenting to take part in the study.

#### Procedure

Each individual participant was seated comfortably in front of the piano keyboard, which was placed on a table, designed to be a dedicated playing station (see S6B Fig). He or she was alone in the recording studio with only the experimenter present. The experimenter was seated next to the participant at the control station (S6C Fig), not facing the keyboard or the participant. The participant was asked by the experimenter to produce improvisations for four musical tasks, which were described each to him/her, and which he or she then carried out, one after the other. The improvisation tasks were not limited in time. The first task the participant was asked to improvise was a positive feeling. The second was a negative feeling. For the third and fourth tasks the individual was asked to improvise the notions of beautiful and ugly. The order of these tasks was counterbalanced switched between the participants to avoid emotional fixation. For example, a negative emotion may condition one’s reaction and might produce a bad mood when playing a positive emotion. Preceding this, the research intentions and full procedure were explained to the participant (see full instructions in S6D Fig). Before the actual improvisation tasks, the participant was acquainted with the keyboard by being allowed to use it freely with no time limit.

#### Statistical analysis

The 108 participants improvised the notion of “ugly” (*n* = 103), “beautiful” (*n* = 108), “negative feeling” (*n* = 108) and “positive feeling” (*n* = 107). One participant did not improvise “positive feeling” and five did not improvise “ugly”. The de-identified data set of the participants’ improvisations can be found in S1 File. Statistical analysis was performed using MATLAB’s Statistic Toolbox [54]. For finding mean improvisation tasks’ parameter differences within subjects, repeated measures Anova was used, and subsequently, Bonferroni method was used for the multiple comparisons procedure to identify the differences among tasks groups. Full statistical analysis output including can be found in S2 File. For finding demographic differences of gender, age and proficiency level in improvisation making, independent two sample *t-*test for means (α = 0.05) was used. With a total number of improvisation samples of 426, mean differences of improvisation parameters were tested between females (*n* = 212) and males (*n* = 214); laymen (*n* = 216) and professionals (*n* = 210); and young (*n* = 209) and old (*n* = 217). See the Results section for the latter grouping considerations. Two-sided testing was used to identify mean differences, as well as one-sided right and left testing to evaluate the difference type; that is, whether the alternative hypothesis of the mean of one group was greater or lesser than the other. Full statistical analysis output can be found in S3 File. Even though the data is relatively normally distributed, normality is assumed for the sampling distribution of the means, which allows mean hypotheses testing. This assumption is based on the Central Limit Theorem and the Law of Large Numbers, that is, the distribution of sample means approaches normality as the size of *n* increases, regardless of the shape of the population’s distribution, and here the sample size is relatively large (i.e., *n≥*30), for all mean hypotheses tests carried out.

#### Ethics statement

The research protocol was reviewed and approved by Bar-Ilan University’s Ethics Committee. All participants signed a written informed consent.

## Results

### The effects of musical improvisation tasks

#### Analysis of individual emergent behaviors

**Fig 3:**
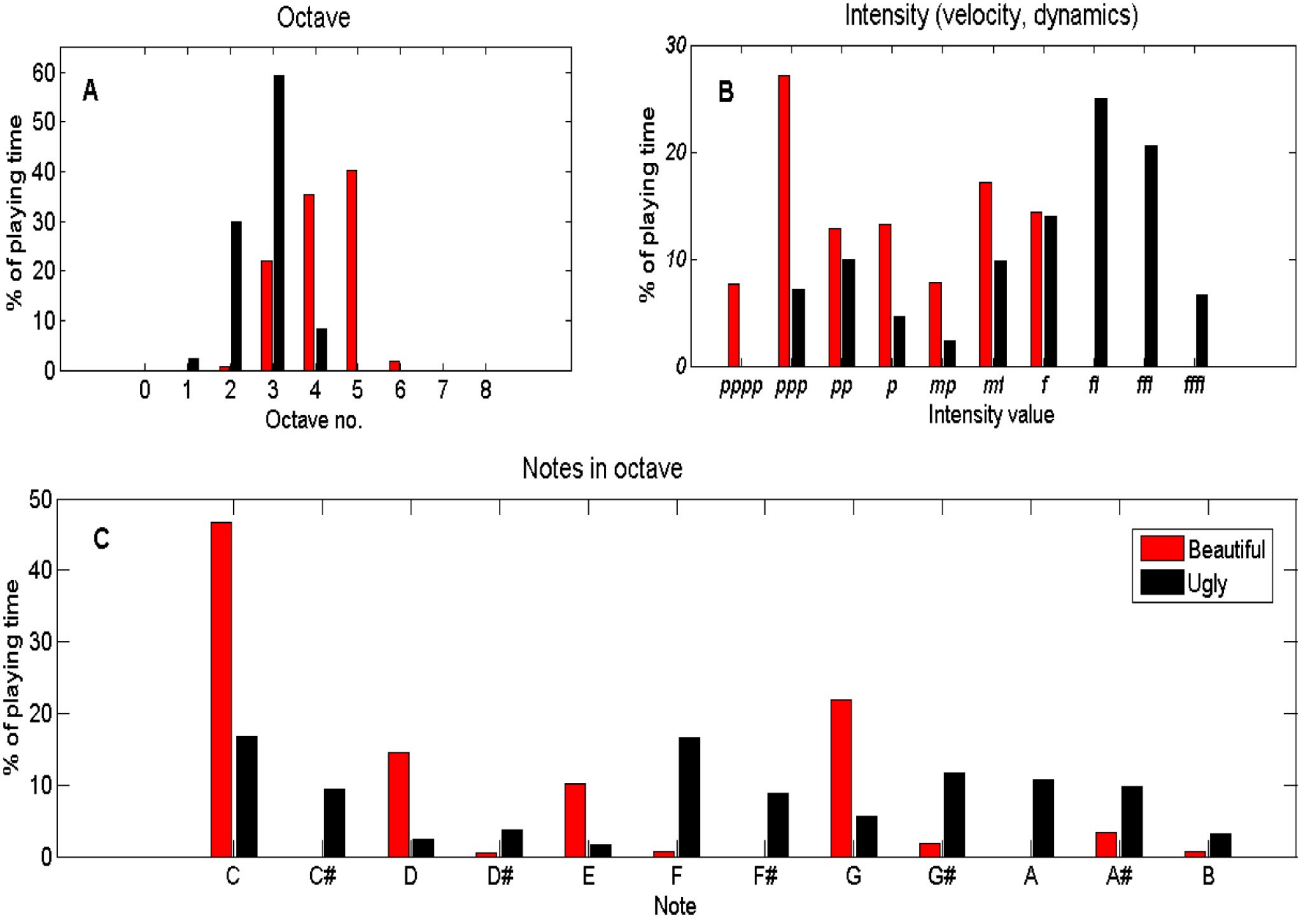
Visual comparison of the improvisations of “ugly” and “beautiful” of an individual participant. These correspond to the improvisations’ timeline appearing in S2 Fig and S1 and S2 Audio Files. (A) Histogram presentation of the octave number as percentage of the playing time. In “ugly”, the octave most used is no. 3, whereas for improvising “beautiful” it is octave no. 5. The histogram also shows that the higher pitch section of the keyboard was used for the latter. (B) Intensity histogram showing that “ugly” was played with more intensity than “beautiful”. Note that the *ff* (fortissimo) was the value most used in improvising the former but *ppp* (pianississimo) in the latter. (C) The pitch classes, as a percentage of playing time, showing that the black keys were preferable when improvising “ugly” and the white keys for “beautiful”; most notably the notes C and G.

We were able to point to phenomena that consist of complex events and their exact time durations, and which are likely to be missed if one relies only on the human observer. For example, given the “ugly” improvisation task to a participant, we captured the number of simultaneous/parallel key presses (S1 Fig), which accurately tracked and documented the fact that the participant used his or her ten fingers to carry out the improvisation and/or other body parts (e.g., his full arm, allowing more than ten keys to be pressed together). This is also important especially with disabled and diseased clients for their assessment and progress. We also compared the dynamics of multiple improvisations. For example, the differences between the “ugly” and “beautiful” improvisations played by another participant (S1 and S2 Audio Files, respectively). We captured and tracked the note choices (S2A Fig) and the pressure exerted on the keys; i.e., the intensity or musical dynamics (S2B Fig). We then analyzed the improvisations’ dynamics according to the parameters (S3 Fig), which include the keyboard use, that is, the octave and intensity range, and pitch classes preferences, all computed over time (Fig 3). These yielded precise quantitative differences between the two individual’s expressive behaviors, thus enabling objective comparison and interpretation.

The method’s capabilities can serve in evaluation and diagnosis, and also in determining the progression of a therapy session; that is, its micro-analysis, where the focus is on specific moments within it, and macro-analysis, with reference to wider perspectives, across sessions, individuals and collectives. We now discuss the latter.

#### Analysis of collective emergent behaviors

**Fig 4:**
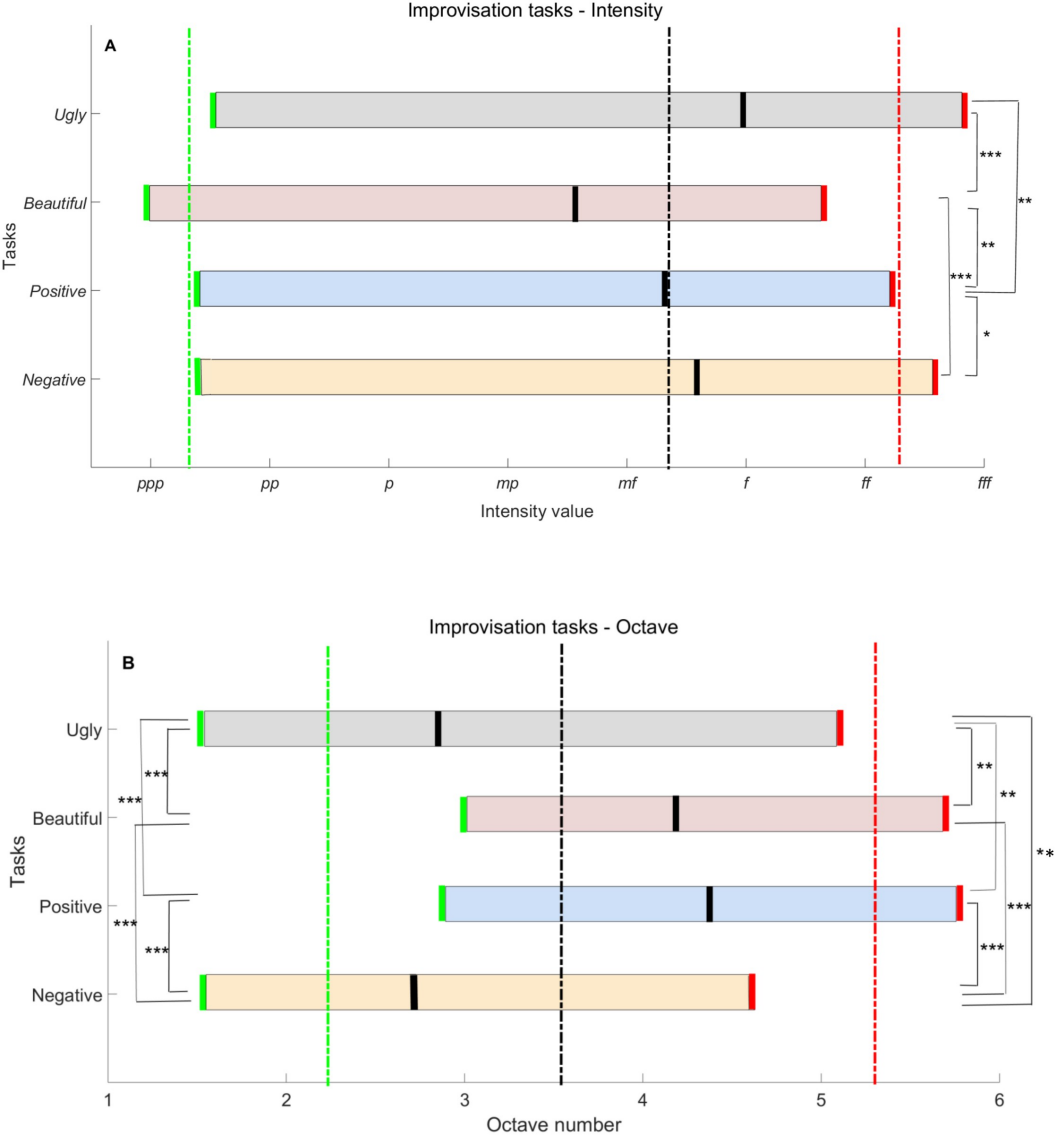
Improvisation task differences in keyboard use. Shown here are the mean (A) intensity values and (B*)* octave numbers for the task collectives. Marked in green is the minimum value, in red the maximum value, and in black the most used value. The respective average values of all improvisations appear in dashed lines. *p < .05, **p < .01, ***p < .001. 1-*pppp*; 2-*ppp*; 3-*pp*; 4-*p*; 5-*mp*; 6-*mf*; 7-*f*; 8-*ff*; 9-*fff*; 10-*ffff*. Statistically significant differences were also obtained between “ugly” and “beautiful”, “ugly” and “positive”, “beautiful” and “negative”, and “beautiful” and “positive” for most used intensity, as well as between “ugly” and “beautiful”, “ugly” and “positive”, “positive” and “negative”, and “beautiful” and “negative” for most used octave. Significance of highest intensity value is obtained with *F* = 19.67, *p* < .0001, effect size η^2^ = .12; most used intensity value obtained with *F* = 14.72, *p* < .0001, η^2^ = .1; lowest octave value obtained with *F* = 55.11, *p* < .0001, η^2^ = .3; highest octave value obtained with *F* = 17.81, *p* < .0001, η^2^ = .12 and most used octave value obtained with *F* = 60.33, *p* < .0001, η^2^ = .31.

**Fig 5:**
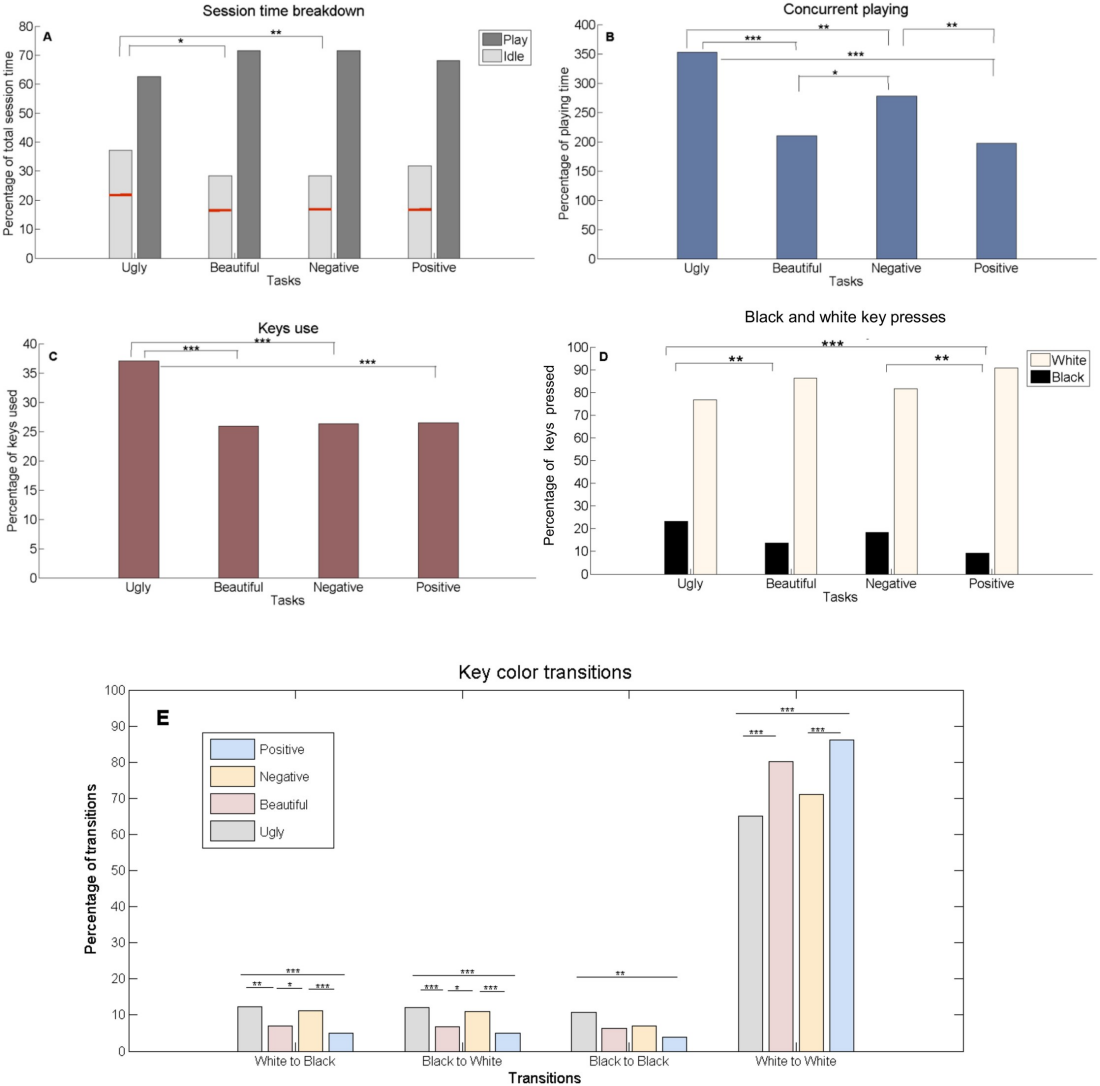
Improvisation task differences in keys and time use. Computed mean values of the task collectives: (A) The session time, broken down into the percentages of playing time (dark gray) and of idle time (light gray) (*F* = 3.98, *p* = .008, η^2^ = .03). The red line on the idle time bar depicts the percentage of time passed since the actual start of the improvisation; i.e., the percentage of time passed until the first note was pressed (*F* = 2.788, *p* = .04, η^2^ = .02). (B) Concurrent playing metric, quantifies the percentage of concurrent playing time per net session play time yielded by keys pressed in parallel (e.g., two keys pressed throughout the session play time yield 200%) (*F* = 19.55, *p* < .0001, η^2^ = .13). (C) Percentage of keys used (*F* = 9.8, *p* < .0001, η^2^ = .07). (D) Black and white key use, quantified as the percentage of presses on black and white keys (*F* = 10.31, *p* < .0001, η^2^ = .07). (E) Black and white key transformations, quantified as the percentage of key presses from white to black (*F* = 11.83, *p* < .0001, η^2^ = .08), black to white (*F* = 11.57, *p* < .0001, η^2^ = .08), black to black (*F* = 4.8, *p* = .003, η^2^ = .03) and white to white (*F* = 11.84, *p* < .0001, η^2^ = .08). *p < .05, **p < .01, ***p < .001.

The improvisations carried out by the participants were grouped according to the four improvisation tasks. As seen in Figs 4 and 5, investigation of collective behaviors yielded significant expressive mean differences when comparing the “ugly” group of improvisations against the collective of “beautiful” improvisations, and “negative feeling” against “positive feeling” (“positive” and “negative” for short, respectively). Furthermore, even though the differences in expressing “beautiful” versus “positive” and “ugly” versus “negative” seem a-priori subtle valence wise, statistically significant emergent behaviors were also obtained by our CP, which unravels empirical differences in title expression.

The *F* statistic reported throughout this section is for *F*(3,404). See the legends of Figs 4 and 5 and the text body. The subscripts *u*, *b*, *n* and *p* identify the group task for the reported mean (*M*) and standard error of mean (*SEM*) values. That is, *u* for “ugly”, *b* for “beautiful”, *n* for “negative” and *p* for “positive” (e.g., *M*_*u*_ and *SEM*_*p*_)_._

Both the “ugly” and “negative” tasks resulted in stronger pressed keys, i.e., notes with higher intensity (Fig 4A), which were played on the lower part of the keyboard, i.e., notes with lower pitch (Fig 4B) as compared to “beautiful” and “positive”. Notably, as seen in Fig 4A, significant differences were obtained between the mean highest used intensity values in a comparison of the “ugly” and “beautiful” tasks, with *p* < .0001 (*M*_*u*_ = 8.8, *SEM*_*u*_ = .12, *M*_*b*_ = 7.6, *SEM*_*b*_ = .11). In addition, the improvisation for “ugly” was played mostly with the intensity of forte (*f*), whereas “beautiful” was played mostly with intensity stronger than mezzo piano (*mp*), a significant difference, with *p* < .0001 (*M*_*u*_ = 7, *SEM*_*u*_ = .15, *M*_*b*_ = 5.6, *SEM*_*b*_ = .16). Furthermore, as displayed in Fig 4B, comparison of octave use for these tasks, resulted in obtaining significant mean differences between lowest, highest and most used octave with respective values of (*M*_*u*_ = 1.5, *SEM*_*u*_ = .08, *M*_*b*_ = 3, *SEM*_*b*_ = .14, *p* < .0001), (*M*_*u*_ = 5.1, *SEM*_*u*_ = .15, *M*_*b*_ = 5.7, *SEM*_*b*_ = .1, *p* < .01) and (*M*_*u*_ = 2.9, *SEM*_*u*_ = .12, *M*_*b*_ = 4.2, *SEM*_*b*_ = .11, *p* < .0001). Whereas “ugly” was mostly played on the left side of the keyboard, i.e., in low octaves, “beautiful” was mostly played on its middle part, that is, medium pitch notes. For the “negative” versus “positive” improvisations, the mean lowest, highest and most used octave, had significant differences, all with *p* < .0001, and with values of (*M*_*n*_ = 1.5, *SEM*_*n*_ = .08, *M*_*p*_ = 2.9, *SEM*_*p*_ = .12), (*M*_*n*_ = 4.6, *SEM*_*n*_ = .17, *M*_*p*_ = 5.8, *SEM*_*p*_ = .09) and (*M*_*n*_ = 2.7, *SEM*_*n*_ = .12, *M*_*p*_ = 4.4, *SEM*_*p*_ = .1), respectively.

When comparing “beautiful” and “positive”, although a similar range of octaves was used, the former task “beautiful”, induced softer improvisations (lowest intensity of *ppp*, pianississimo), whereas the latter task, “positive”, exhibited louder improvisations (lowest intensity below *pp*), which is also due to participants tending to play jolly/happy music, that is, pressing the keys with more pressure (the mean highest and most used intensity differences are with values (*M*_*b*_ = 7.6, *SEM*_*b*_ = .11, *M*_*p*_ = 8.2, *SEM*_*p*_ = .13, *p* = .003) and (*M*_*b*_ = 5.6, *SEM*_*b*_ = .16, *M*_*p*_ = 3.6, *SEM*_*p*_ = .13, *p* = .003), respectively, and with intensity values almost as strong as those of the “negative” task.

Analysis of the behavioral differences between “ugly” and “negative” shows that the participants pressed the keys more strongly during the “ugly” task, and even used a higher octave range (highest value difference is with *M*_*u*_ = 5.1, *SEM*_*u*_ = .15, *M*_*n*_ = 4.6, *SEM*_*n*_ = .17, *p =* .001). Basically, when improvising “ugly”, the participants pressed and hit as many keys as possible, almost as if they were ‘attacking’ the keyboard. This can be also seen in Fig 5. Although the playing percentage time of “ugly” was significantly lower compared to the other tasks (as seen in Fig 5A, e.g., 63% for “ugly” vs. 72% for “negative”, with *SEM*_*u*_ = 2, *SEM*_*n*_ = 2, *p* = .01), the “ugly” improvisation resulted in the participants’ tendency to press more keys in parallel (Fig 5B), with a larger number of keys used (Fig 5C), with a preference to hitting more black keys (Fig 5D) and with chromatic cluster formations (Fig 5E).

Interestingly, the “ugly” improvisation task actually took more time to start (22.2 sec as compared to the 16.4 sec start time of “negative”); that is, this challenge made the participants think longer before starting to play (Fig 5A). When the participant finally did start, as seen in Fig 5C, for “ugly”, 37% of the keyboard’s keys were used, whereas for “negative”, the percentage of the used keys was 26% (*SEM*_*u*_ = 2.3, *SEM*_*n*_ = 1.8, *p* = .0001). In addition, as depicted in Fig 5B, concurrent playing metric (i.e., keys pressed together, for example, 3 keys pressed in parallel throughout the session play time yield 300%) took 353% of the net playing time for “ugly”, in comparison with the 266% of “negative” (*SEM*_*u*_ = 24, *SEM*_*n*_ = 19, *p* = .002) and the 200% of “beautiful” (*SEM*_*b*_ = 11, *p* = .0001). Furthermore, 23% keys of the total number of keys pressed were black for the “ugly” improvisation as compared to the 18% for the improvisation of “negative” and with the 13% of “beautiful” (*SEM*_*u*_ = 1.9, *SEM*_*b*_ = 2, *p* = .002). See Fig 5D. Noticeable also (Fig 5E), was the preference of using the white keys for the “positive” improvisation, and with significantly less chromatic transitions, e.g., black to white (5%) as compared to “ugly” (12.3%), (*SEM*_*p*_ = .8, *SEM*_*u*_ = 1.1, *p =* .001) and “negative” (11.1%), (*SEM*_*n*_ = 1.2, *p =* .0001).

### Emergent demographic variation factors in music making

**Fig 6:**
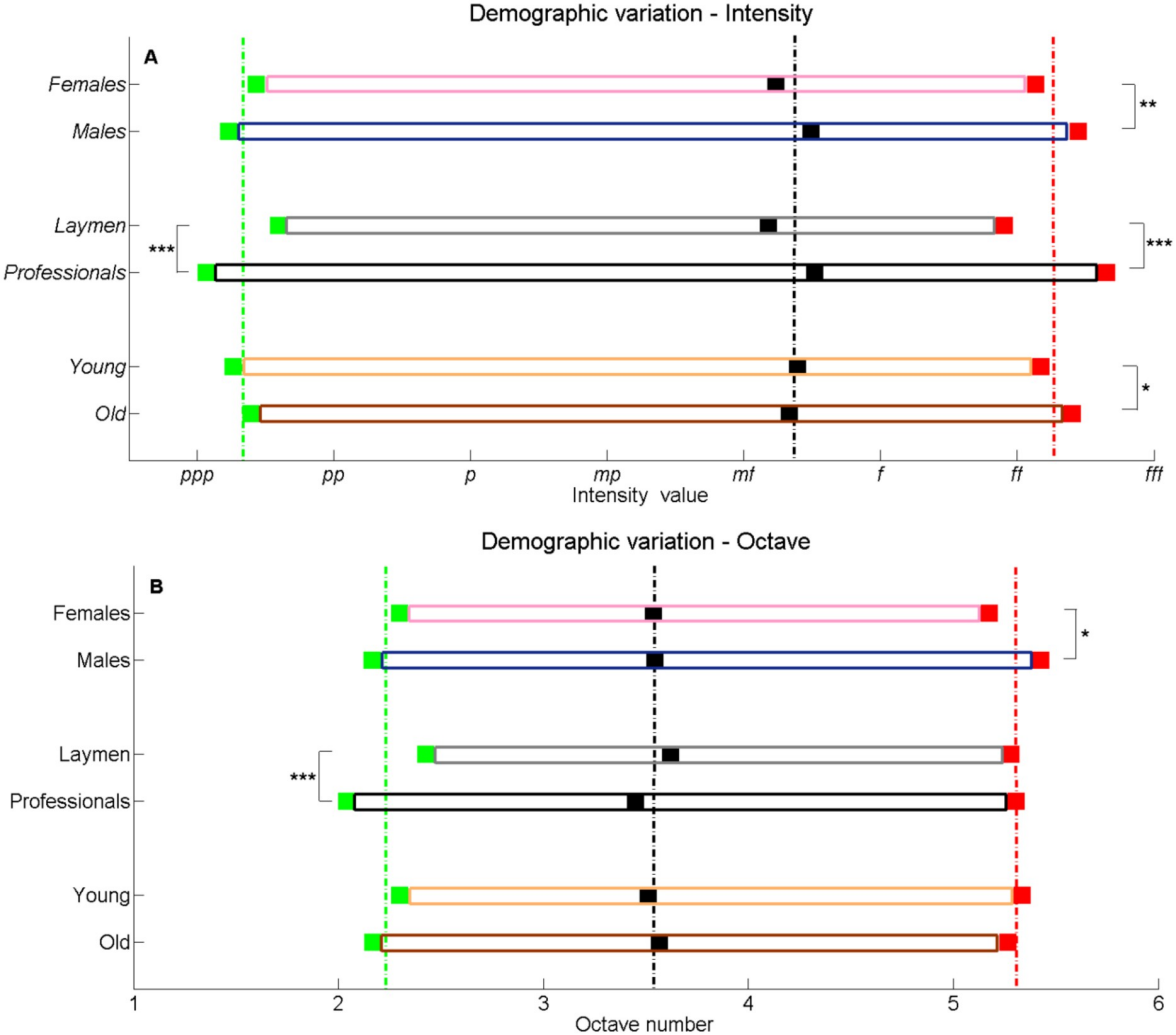
Demographic variation in keyboard use. The ordinate displayed differences between females and males, laymen and professionals, and young and old. The abscissa depicts the mean: (A) intensity values and (B) octave values for the demographic collectives. Marked in green is the minimum value, in red the maximum value, and in black the most used value. The respective average values of all improvisations appear in dashed lines. *p < .05, **p < .01, ***p < .001. 1-*pppp*; 2-*ppp*; 3-*pp*; 4-*p*; 5-*mp*; 6-*mf*; 7-*f*; 8-*ff*; 9-*fff*; 10-*ffff*. Statistically significant differences were also found between laymen and professionals for most used intensity and octave number.

**Fig 7:**
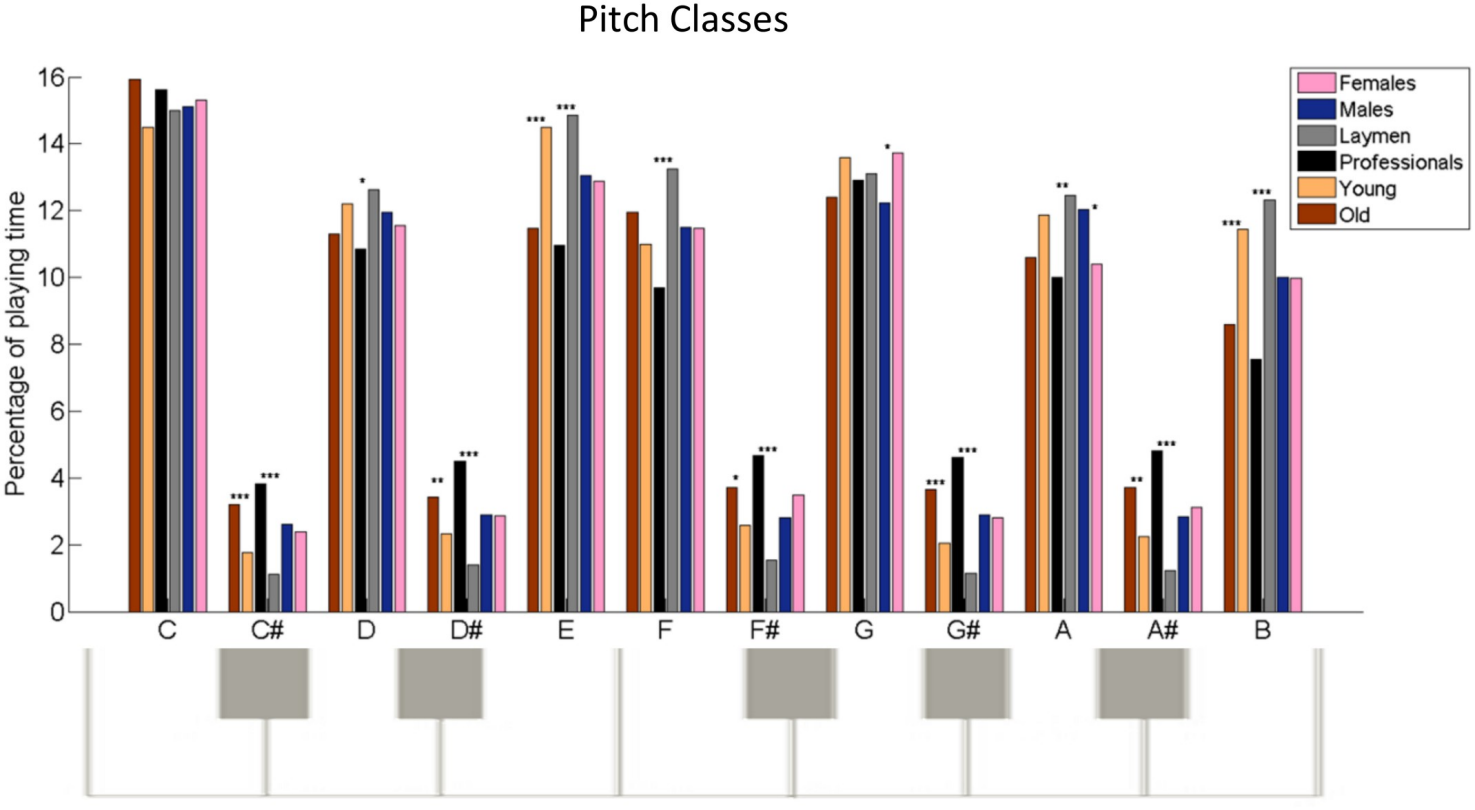
Demographic variation in pitch classes preference. Notes used in the improvisations are “collapsed” onto an octave to yield the mean differences in the percentage of playing time between the collectives of females and males (pink and blue bars, respectively), laymen and professionals (gray and black bars, respectively), and young and old (orange and brown bars, respectively). *p < .05, **p < .01, ***p < .001.

We also studied the demographic differences among the participants. The improvisations were categorized according to gender, age and proficiency level. Behavioral analysis of these yielded significant variation factors between females and males, young and old, as well as between lay persons (laymen) and professionals. See Figs 6 and 7 and Table 1. The subscripts *f*, *m*, *l*, *pr*, *y*, and *o* identify the categories, that is, *f* for females, *m* for males, *l* for laymen, *pr* for professionals, *y* for young, and *o* for old.

**Table 1:** Parameter comparison in improvisation making of collectives.

*Attribute*	*Parameter*	*Average*	*Females*	*Males*	*Laymen*	*Profess-ionals*	*Young*	*Old*
**Time**	% playing time	69 (1)	70 (1.5)	67 (1.5)	62 (1.5)	75(1.3)***	70 (1.4)	68 (1.5)
	% idle time	31 (1)	30 (1.5)	33 (1.5)	38(1.4)***	25 (1.3)	30 (1.4)	32 (1.5)
	% start time	18 (.5)	18 (1.2)	18 (1)	20 (1.2)**	16 (1)	17 (1)	20 (1.5) *
	% concurrent	259 (9)	257 (13)	260 (12)	211 (12)	305 (12) ***	225 (9)	291 (15)***
	total (minutes)	0.86 (.07)	0.76 (.1)	0.95(.09)	0.47 (.04)	1.25(.12)***	0.83 (.1)	0.88 (.09)
**Notes/ Keys**	# of presses	162 (20)	118 (18)	206 (37)*	82 (14)	242 (39)***	183 (37)	143 (17)
	% used	29 (1)	27 (1.2)	31(1.4) *	23 (1.1)	35 (1.3) ***	27 (1.3)	30 (1.3) *
	presses per key	5.9 (.4)	4.7 (.3)	7.2 (.8) **	4.0 (.3)	7.8 (.8)***	6.2 (.8)	5.7 (.4)
	play per key (sec)	0.4 (.02)	0.42(.03)	0.37(.02)	0.38 (.02)	0.4 (.02)	0.38 (.02)	0.41 (.03)
	% black presses	16 (1)	17 (1.4)	16 (1.3)	7 (1)	25 (1.4)***	12 (1.2)	20(1.5)***
	% white presses	84 (1)	83 (1.4)	84 (1.3)	93 (1) ***	75 (1.4)	88(1.2)***	80 (1.5)
**Intensity**^†^	average	6.0 (.06)	5.9 (.09)	6.1 (.09)	5.9 (.1)	6.1 (.09)	6.0 (.09)	6.0 (.1)
	lowest (minimum)	2.3 (.07)	2.4 (.1)	2.3 (.1)	2.6 (.11)***	2.1 (.09)	2.3 (.1)	2.4 (.1)
	highest (maximum)	8.3 (.06)	8.1 (.09)	8.4(.09) **	7.9 (.08)	8.7 (.08)***	8.2 (.08)	8.4 (.09)*
	most used	6.4 (.08)	6.2 (.11)	6.5 (.11)	6.2 (.11)	6.5 (.11) *	6.4 (.1)	6.3 (.12)
**Octave**	average	3.6 (.06)	3.6 (.08)	3.6 (.08)	3.7 (.1) *	3.5 (.06)	3.6 (.09)	3.6 (.07)
	lowest (minimum)	2.2 (.06)	2.3 (.09)	2.2 (.09)	2.4 (.1) ***	2.0 (.07)	2.3 (.1)	2.2 (.08)
	highest (maximum)	5.3 (.07)	5.2 (.1)	5.4(.09) *	5.3 (.11)	5.3 (.08)	5.3 (.1)	5.3 (.09)
	most used	3.5 (.07)	3.5 (.09)	3.5 (.1)	3.6 (.11)	3.4 (.07)	3.5 (.11)	3.6 (.08)
**Cluster of notes**	# of instances	300 (40)	219 (34)	381(72) *	141 (26)	460 (76)***	338 (74)	264 (33)
	max pressed^‡^	5.8 (.2)	5.6 (.3)	5.9 (.3)	4.7 (.2)	6.8 (.33)***	5.1 (.2)	6.4 (.3) ***
	most pressed^§^	2.4 (.1)	2.4 (.2)	2.3 (.1)	1.9 (.1)	2.8 (.2) ***	2.0 (.1)	2.7 (.2) ***
	% most played^||^	56 (1)	57 (1.8)	55 (1.8)	65(1.7)***	47 (1.6)	58(1.8)***	53 (1.6)
**Transit-ions**	% diminuendo	47 (.3)	46 (.4)	47 (.4)	45 (.5)	48 (.3)***	47 (.4)	46 (.4)
	% crescendo	50 (.3)	50 (.4)	50 (.4)	51 (.5) ***	48 (.3)	50 (.4)	50 (.5)
	% same intensity	3 (.2)	4 (.3)	3 (.2)	4 (.3)	4 (.3)	3 (.2)	4 (.3)
	% accelerando	11 (.5)	11 (.7)	11 (.7)	8 (.6)	14 (.7) ***	10 (.7)	12 (.7)
	% ritardando	89 (.5)	89 (.7)	89 (.7)	92 (.6)***	86 (.7)	90 (.7)	88 (.7)
	% white to black	9 (.5)	9 (.8)	9 (.7)	4 (.5)	15 (.7) ***	7 (.7)	11 (.7) ***
	% black to white	9 (.5)	9 (.8)	8 (.7)	4 (.5)	14 (.7)***	7 (.7)	10 (.7) ***
	% black to black	7 (.6)	7 (1)	7 (.8)	8 (.6)	10 (1) ***	5 (.7)	9 (1) ***
	% white to white	75 (1.4)	75 (2)	76 (2)	89(1.4)***	61 (2)	81(1.8)***	70 (2)
**Pitch classes**	% playing time	See Fig 7						

Data in cells is presented as: mean (SEM)

* *p* < .05;

** *p* < .01;

*** *p* < .001

^†^ 1-*pppp*; 2-*ppp*; 3-*pp*; 4-*p*; 5-*mp*; 6-*mf*; 7-*f*; 8-*ff*; 9-*fff*; 10-*ffff* (See Fig 6A)

^‡^ configuration of maximum number of keys pressed;

^§^ most pressed configuration;

^||^ relative playing time of the most pressed configuration;

Comparing the improvisations of females and males (Fig 6), ones sees that females were more confined in using the keyboard. Notably, as seen in Fig 6A, the highest intensity (*M*_*f*_ = 8.1, *SEM*_*f*_ = .09) is lower than that of the males (*M*_*m*_ = 8.4, *SEM*_*m*_ = .09), with (*t*(424) = 1.36, *p* = .005, Cohen’s D effect size *d* = .25) (and the lowest intensity is higher than that of males), and in Fig 6B, the highest octave number (*M*_*f*_ = 5.2, *SEM*_*f*_ = .1) is lower than that of males (*M*_*m*_ = 5.4, *SEM*_*m*_ = .09), with (*t*(420) = 1.87, *p* = .003, *d* = .18) (and the lowest octave number is higher than that of males). The octave numbers and intensity values appear in Table 1. This expressive behavior is qualitatively reminiscent of the laymen versus the professionals. Here too, laymen are more reserved in their keyboard use than professionals. Notable are the differences of lowest intensity (*M*_*l*_ = 2.6, *SEM*_*l*_ = .11, *M*_*pr*_ = 2.1, *SEM*_*pr*_ = .09, *t*(414) = 3.6, *p* = .0002, *d* = .35) and octave number (*M*_*l*_ = 2.4, *SEM*_*l*_ = .1, *M*_*pr*_ = 2, *SEM*_*pr*_ = .07, *t*(362) = 3.11, *p* = .001, *d* = .3) as compared to professionals, and of the highest intensity as well (*M*_*l*_ = 7.9, *SEM*_*l*_ = .08, *M*_*pr*_ = 8.7, *SEM*_*pr*_ = .08, *t*(424) = 6.45, *p* < .0001, *d* = .62). Table 1 also shows that males used and pressed more keys than females, which resulted in 7.2 presses per key (*SEM*_*m*_ = .83), as compared to the 4.7 presses of females (*SEM*_*f*_ = .31) (*t*(270) = 2.83, *p* < .0001, *d* = .28). These phenomena are also seen when comparing lay females to lay males and professional females to professional males. Notable, are the significant differences of the total number of presses, and the number of presses per key. For lay females versus lay males, these are 52 (*SEM* = 5.1) versus 83 (*SEM* = 12.6) overall presses (*t*(94) = 2.24, *p* = .03, *d* = .37), and 3.1 (SEM = .3) versus 4.8 (*SEM* = .5) presses per key (*t*(112) = 2.91, *p* = .002, *d* = .49). All these seem to imply that males were more exploratory than females. An analogous implication was obtained in our previous study of gender difference in artwork, where males used more drawing tools than females [42].

In Fig 7, it is seen that laymen preferred to use the white keys more, as compared to the professionals, resulting in 93% (*SEM*_*l*_ = 1) of the number of keys pressed for the former vs. 75% (*SEM*_*pr*_ = 1.4) for the latter, with (*t*(375) = 10.07, *p* < .0001, *d* = .39). Table 1 also shows additional differences between these two collectives, such as the absolute playing time and percentage of total time devoted to playing time, the use and presses of keys, their note clustering characteristics, and transitions; e.g., from playing from soft to loud and vice versa.

The participants were also categorized according to two age groups, termed old and young. Those over the age median (28 years) constitute the older group of participants, and those below are the younger group. As seen in Fig 7, the older group had a preference for playing the black keys, i.e., the C#, D#, F#, G# and A# notes, whereas the younger group preferred the white keys. For example, the old used C# for 3% (*SEM*_*o*_ = .4) of the playing time whereas the young for 2% (*SEM*_*y*_ = .2) (*t*(382) = 3.4, *p* = .0004, *d* = .33). Furthermore, as seen in Table 1, the old group improvised with keys pressed concurrently for a larger percentage of net playing time than the young, *M*_*o*_ = 291% vs. *M*_*y =*_ 225% (*SEM*_*o*_ = 9, *SEM*_*y*_ *=* 15, *t*(352) = 3.79, *p* < .0001, *d* = .36), with the percentage of more keys used, *M*_*o*_ = 30% vs. *M*_*y*_ = 27% (*SEM*_*o*_ = 1.3, *SEM*_*y*_ *=* 1.3, *t*(424) = 1.69, *p* < .05, *d* = .04), and with larger cluster sizes, 6.4 keys for the former as compared to 5.1 keys for the latter (*SEM*_*o*_ = .3, *SEM*_*y*_ *=* .2, *t*(374) = 3.22, *p* = .0007, *d* = .31). These phenomena were similar when we compared young laymen to old laymen and young professionals to old professionals. Noticeable are the concurrent playing and black key preference of old laymen versus young laymen. That is, 215% (*SEM* = 18.6) versus 176% (*SEM* = 12) of concurrent playing (*t*(121) = 1.77, *p* = .04, *d* = .3), and 10% (*SEM* = 1.8) versus 3% (*SEM* = 1) black key presses (*t*(113) = 3.21, *p* < .001, *d* = .53). Similar is the keyboard use (Fig 6A and 6B), for which the old tend to play at a lower pitch and more intensely. The age differences in the behavioral parameters obtained are reminiscent of the differences of those expressed in carrying out the different tasks. That is, the results of old versus young are qualitatively similar to the results of the “ugly” versus “beautiful” and “negative” versus “positive” tasks. To simplify this exposition, we collapsed the “ugly”/“negative” and “beautiful”/“positive” into general bipolar “valence” dimensions. See S1 Table. This may suggest that as we grow older we tend towards a negative mood or state-of-mind, as discussed in the next section. This implied phenomenon was also seen in the age difference of our artwork study [42], where the older group used fewer colors than the younger group, and erased more.

## Discussion

### Summary and implications to music therapy

We have implemented our computational paradigm (CP) [42] for the music modality and then applied it in a proof-of-principle study to elucidate behavioral effects induced by music making, investigating the expressive behavioral response of individuals and collectives to several improvisation tasks.

Significant demographic differences emerged; i.e., gender, age and proficiency level differences, which point to collective variation factors in music making. For example, males were more exploratory in their keyboard use than females. This is compatible with findings that show gender difference in spatial and exploration abilities, i.e., males are engaged in wide-range exploration, and explore more than females [55,56]. However, further research is required to conclude whether these differences are biologically innate (as implied by cognitive studies that show gender differences in spatial abilities [57,58]) or culturally acquired through gender socialization [59]. Furthermore, the old versus young behavioral difference was also seen in the comparison between the “negative”/“ugly” versus the “positive”/“beautiful” behavioral patterns, exhibiting a possible change from general bipolar valence positive to negative moods or state-of-mind as we grow old. This is in line with the well-known gradual personality changes throughout life, where personality in older age becomes quite different from personality in childhood [60,61]. Although research into musical development in a lifespan perspective has been accumulating in the past decade or so [62,63], more specific research is required to conclude whether improvisational abilities and expressiveness change from younger to older ages, and if so—why. Here, we also suggest a changed characteristic. Providing the music therapist with these empirical findings can supply him or her with explicit knowledge of demographic variation factors as one of the causes of behavioral diversification. That is, the age, gender and proficiency level factors may be accounted for in treatment design and may help ameliorate its efficacy. Additional factors, such as ethno-cultural background and disorder/illness/pathology type can also be studied using our CP to account for further variation in response to musical interventions.

In addition, task-based behavioral patterns of musical expressivity were identified empirically, exhibiting significant differences between them, and revealing the dynamic nature of “ugly” expression, as well as that of “beautiful”, “negative” and “positive”. By providing empirical evidence of improvisation title differentiation, our CP can be used for designing diverse musical tasks and/or musical interventions even with subtle nuances. As such, and since the behaviors are rigorously identified and quantified, the method could serve as an empirical platform for comparing these against known patterns, so as to test and map tasks and interventions, and could also yield a “titled” library of behavioral patterns to serve music therapists and researchers. For example, our CP can be used in assessment and evaluation in therapy, e.g., in analyzing free style improvisation and correlating the response to the known expressive patterns.

Furthermore, the emerged differences when comparing the titled improvisation tasks may suggest that a-priori restricting the keyboard use to the overall preference obtained by the participants per task, might be tested to generate a particular mood in which the therapist wishes the patient to be, through playing. For example, we postulate that limiting the range of octaves, intensity values and key color to the significant outputs obtained by the collective of participants in response to, say, the “positive” task; i.e., high octaves, low intensity and white keys, may induce such a feeling in a study that also measures emotional mood state. See [64] which exemplifies this notion for the movement modality and [65] for passive music listening. It may also be interesting to further test key color preferences. Evidently, “ugly” (as compared to “beautiful”) was characterized by significant high percentage of chromatic white-to-black and black-to-white transitions and low percentage of diatonic white-to-white transitions. However, further CP based studies can explore whether the black key color significant preference in “ugly” vs. “beautiful” is due to its color or to the mere fact that the black keys are physically raised and are narrower than the white ones, and hence are more amenable to the brute force and concurrent presses observed, as well as to the chromatic clustering of their formation. Reversing the keyboard colors, and/or using an all-white or all-black keyboard may facilitate providing an answer in further experimentation.

Finally, the behavioral results we generate; e.g., task-based and demography-based, may serve as systematic prediction leads to brain activity and bio-neural mechanisms mapping [66,67].

### Future goals and implementations of the CP

We now discuss additional technological expansions of our CP (schematically appearing in Figs 1 and 2) and its use in systematic and mechanistic musical behavior investigations aimed for further scientific and clinical research.

#### Modeled tracking module

Our method is designed to capture additional musical instruments other than the one we used here, i.e., a piano keyboard. These include percussion, woodwinds and string instruments [68–70]. This capability may be used in studies where the patient’s choice of instrument is investigated. In the future, we plan to expand the method to accommodate acoustic instruments too. We also expect to further implement the CP to capture occurrences in 3-dimensional and audio space. This data will be the input to the patient and therapist models we plan to develop. These models will track bodily and auditory dynamics, narrating social interaction; e.g., facial expressions, body language and therapist intervention. This will enable studies along the right side of the ‘music as therapy’ ↔ ‘music in therapy’ continuum; i.e., music-based approaches, where the therapist-patient interaction is also considered a focus of attention, and will enable model development for the dance/movement modality. Initial steps in these directions can be found in S4 Fig and in [42].

#### Analysis module

The Analysis module, which is study-based, analyses the emergent behaviors stemming out of the Modeled Tracking module per devised study. As such, in addition to the aforementioned studies, we plan to employ our CP in studies related to clinical settings for discovering ‘behavioral markers’; i.e., determining which of the parameters (as in Table 1) evaluate session progress and outcome. An example would be quantifiably discovering ‘moments-of-change’ within a session and/or a succession of sessions [24], which will be parameter-based. A currently ongoing study employing our CP is aimed at this. It consists of a therapist and a client in a clinical setting. We empirically investigate treatment progression and its outcome for several clients throughout a series of sessions each client participates in. A potential evolution of this goal is to also notify the therapist, in real time, of changing ‘behavioral markers’ throughout the session(s). The CP can also be employed in studies that characterize individual dynamics of recreational music making. For example, sight reading and performance comparison [71].

#### Documentation module

We have taken some initial steps to enable reporting patient cases and behavioral patterns, hoping to eventually devise an appropriate formal language for representing the dynamics of the clinical session domain. This will allow session comparisons and documentation, retrieval and sharing of information. Examples of preliminary graphical notations for music therapy sessions can be found in [36] and [37]. We plan to further develop these, and our current textual and visual reports of musical session dynamics (e.g., Fig 3 and S1–S3 Figs), yielding automated or semi-automated domain-specific languages. When an agreed-upon language is adopted in a domain of activity, it will enable numerous opportunities for communication and understanding between specialists and communities of the domain’s relevant fields. It is our hope to contribute to this quest [72].

We believe that our approach has the potential of helping make progress in fields employing the arts in general and music in particular, such as healthcare, psychology, social work, education, and recreation, in both scientific research and clinical settings.

## Supporting information

S1 Fig Depiction of an improvisation of “ugly”. The improvisation was carried out by a 67 year old male; its timeline is shown on the abscissa. The ordinate: the keyboard layout where Cn denotes the note C and the octave number it is in. The black dots represent the keys pressed. Note that there are clusters of more than ten keys pressed in parallel. This also shows that the participant improvised not only with his fingers, i.e., and/or with other body parts, for example, his arm. See S2A Fig for improvisations where cluster sizes were less than ten keys.
(TIF)

S2 Fig Improvisations of “ugly” and “beautiful” depicted over time. These two improvisations were carried out by a 29 year old female. (A) The ordinate displays the keyboard marked by the C notes and the octaves they are in, whereas the notes pressed for “ugly” and “beautiful” appear as black and red dots, respectively. (B) The intensity values for the notes pressed in (A) appear as dots ranging from *pppp* to *ffff* (ordinate). The two improvisations can be heard by playing the S1 and S2 Audio Files, respectively.
(TIF)

S3 Fig The comprehensive textual report for an improvisation of “ugly”. The improvisation can be heard by playing S1 Audio File. Its graphic depiction appears in S2 Fig in black.
(TIF)

S4 Fig The top view of the system’s model. The Statecharts visual formalism [44] modeling the music room and three concurrent/orthogonal states (dashed lines) specifying the entities therein: the **Music_work**, **Client** (patient) and **Music_Therapist**. The figure also shows the events that trigger the beginning of the therapy session and its termination, specified as mutually exclusive states, **MusicRoomSessionOn** and **MusicRoomSessionOff**, respectively (with solid lines). The **Music_work** creator can be in one of three states: **Playing** (see also S5 Fig), musical instrument **Selecting** or **Idle.**
(TIF)

S5 Fig Hierarchical view of the system’s model. The visual modeling of the system using the Statecharts formalism. (***Top Panel***) The top view of the system, see S4 Fig. (***Bottom Panel***) The **Playing** state, zoomed in, is further decomposed into sub-states formulating the music making process.
(TIF)

S6 Fig Study setup and procedure. The study was carried out in the recording studio in the Music Department of Bar Ilan University. (A) The apparatus—Roland A-30-MIDI keyboard controller, comprised of 76 keys, 31 of them black and 45 white. Piano sound. The auditory feedback through speakers. **(**B) The participant playing station. (C) The experimenter control station. (D) The instructions for the participants given by the experimenter.
(TIF)

S1 Table Similarity in parameter comparison of collectives. (PDF)

S1 Audio An improvisation of “ugly”. (MP3)

S2 Audio An improvisation of “beautiful”. (MP3)

S1 File The participants’ de-identified data set. (TXT)

S2 File Statistical analysis of the improvisation tasks study. (PDF)

S3 File Statistical analysis of the demographic study. (PDF)
