# Supplementary figures and images for: Computational elucidation of the effects induced by music making
Source: PLoS One. 2019 Mar 7;14(3):e0213247. doi: 10.1371/journal.pone.0213247 (PMC6405055; doi:10.1371/journal.pone.0213247)

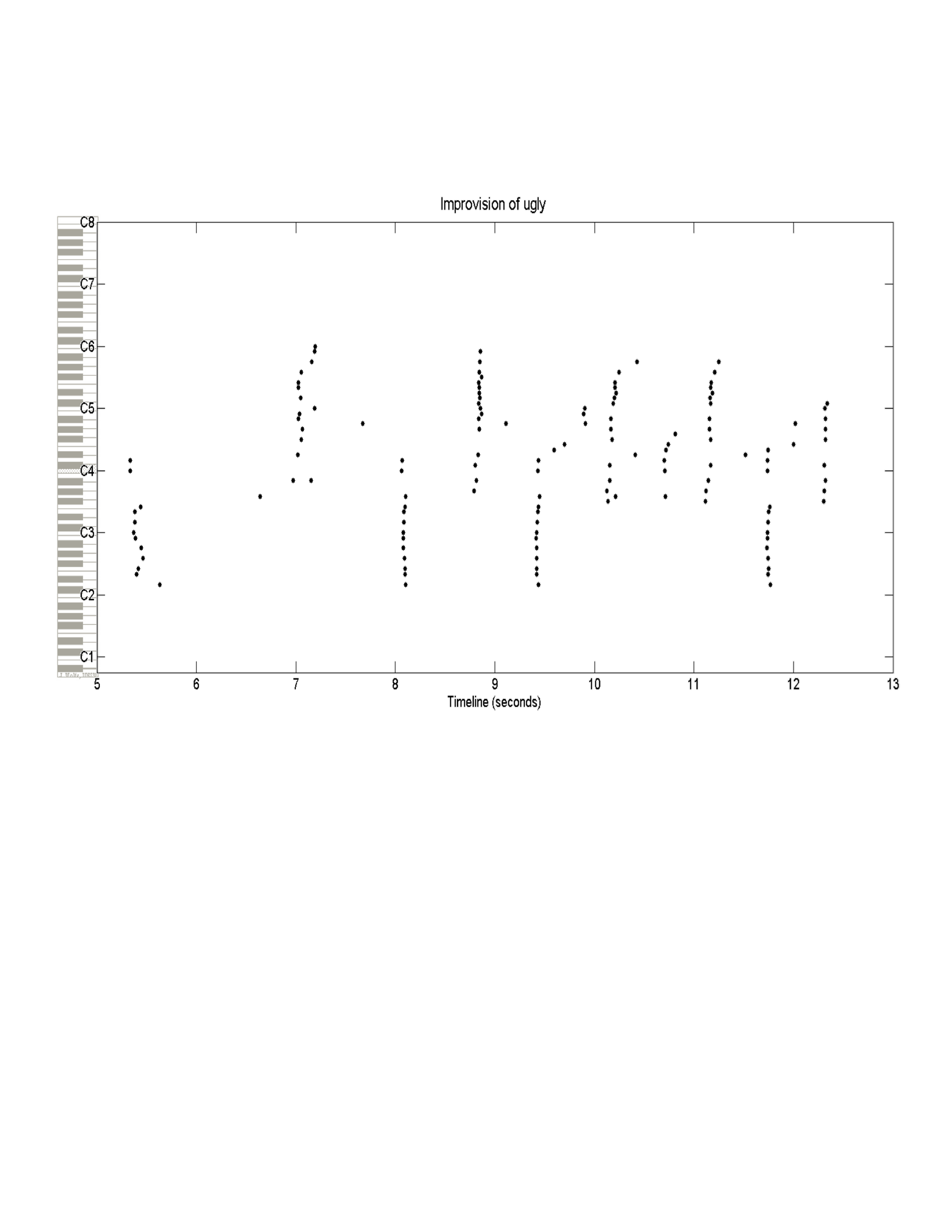

Supplement: S1 Fig — The improvisation was carried out by a 67 year old male; its timeline is shown on the abscissa. The ordinate: the keyboard layout where Cn denotes the note C and the octave number it is in. The black dots represent the keys pressed. Note that there are clusters of more than ten keys pressed in parallel. This also shows that the participant improvised not only with his fingers, i.e., and/or with other body parts, for example, his arm. See S2A Fig for improvisations where cluster sizes were less than ten keys. (TIF) [file pone.0213247.s001.tif]

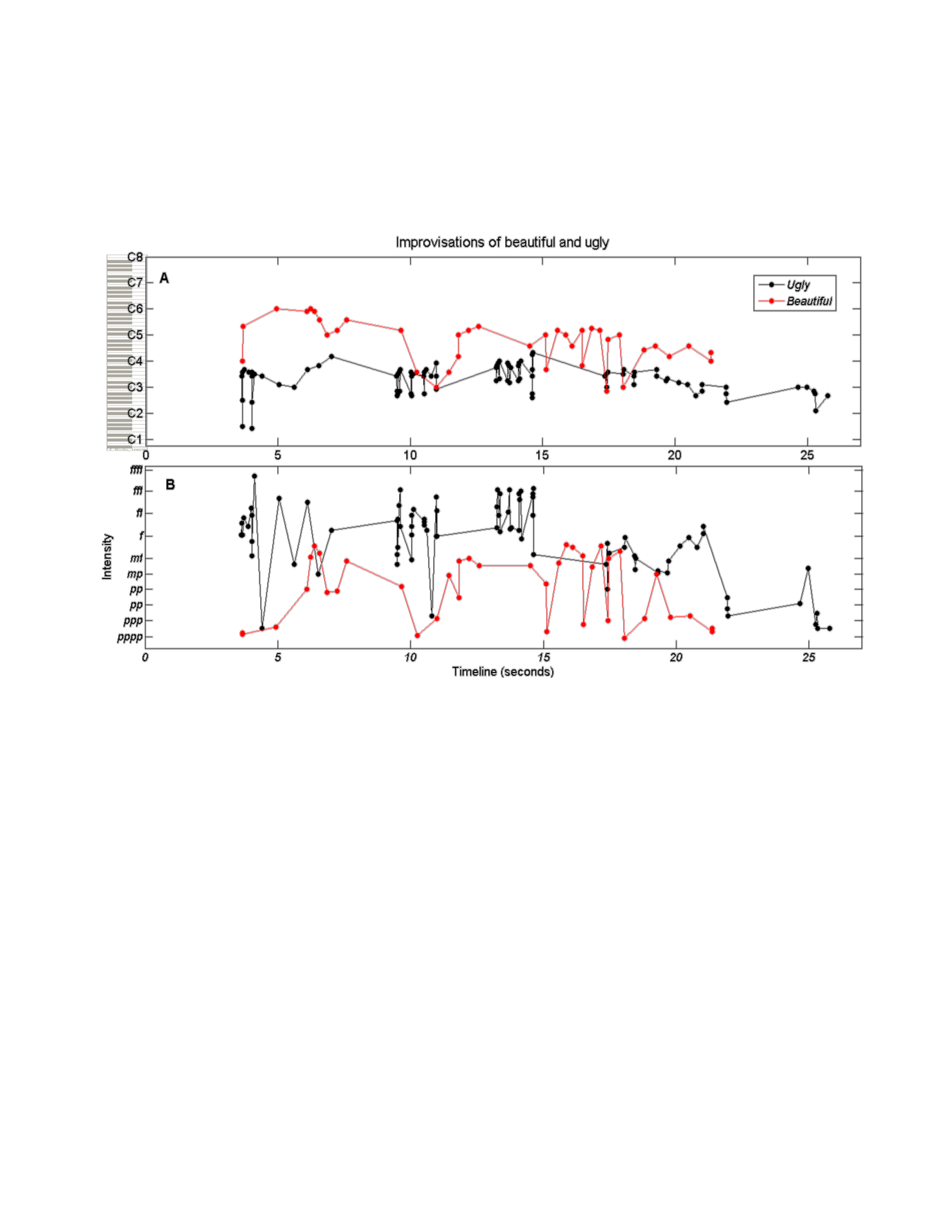

Supplement: S2 Fig — These two improvisations were carried out by a 29 year old female. (A) The ordinate displays the keyboard marked by the C notes and the octaves they are in, whereas the notes pressed for “ugly” and “beautiful” appear as black and red dots, respectively. (B) The intensity values for the notes pressed in (A) appear as dots ranging from pppp to ffff (ordinate). The two improvisations can be heard by playing the S1 and S2 Audio Files, respectively. (TIF) [file pone.0213247.s002.tif]

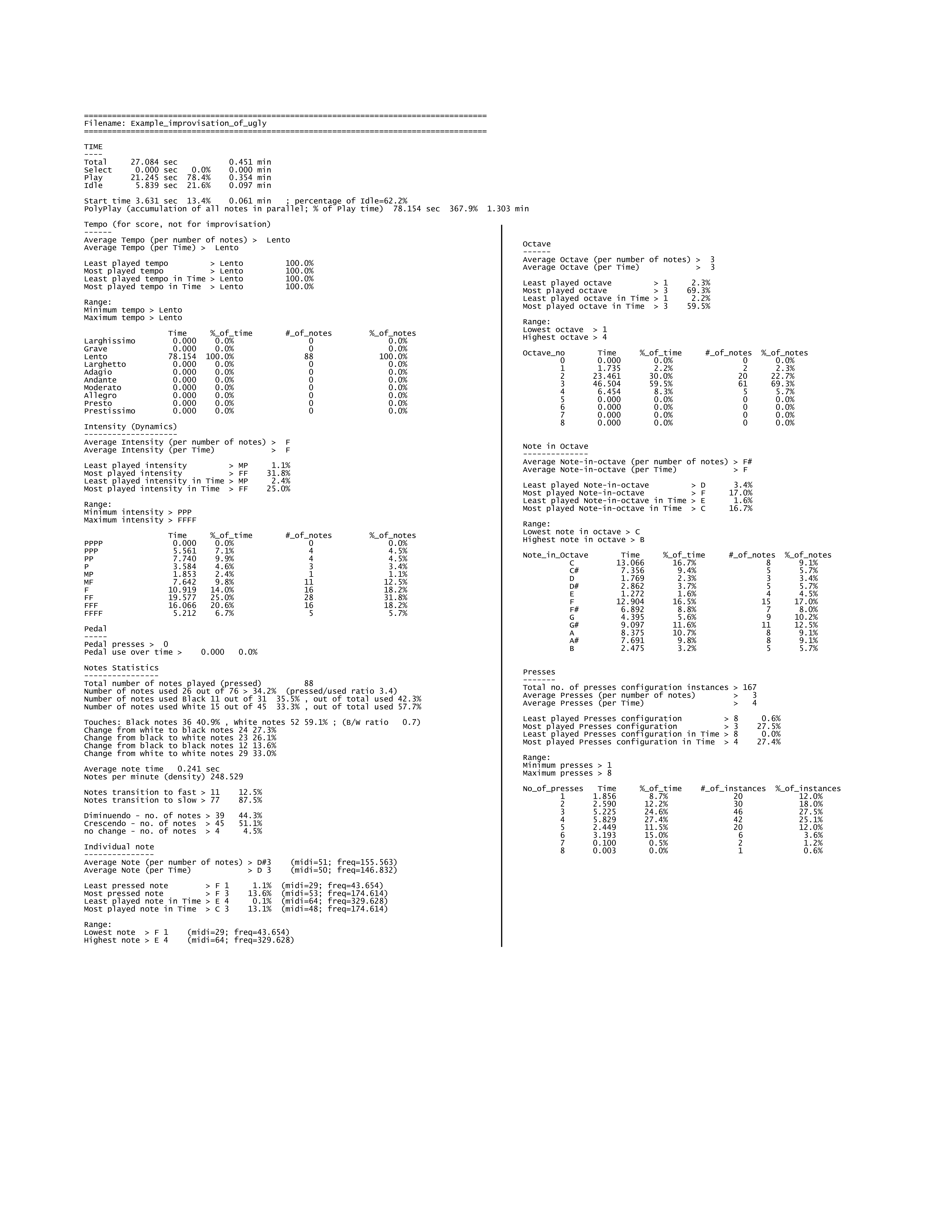

Supplement: S3 Fig — The improvisation can be heard by playing S1 Audio File. Its graphic depiction appears in S2 Fig in black. (TIF) [file pone.0213247.s003.tif]

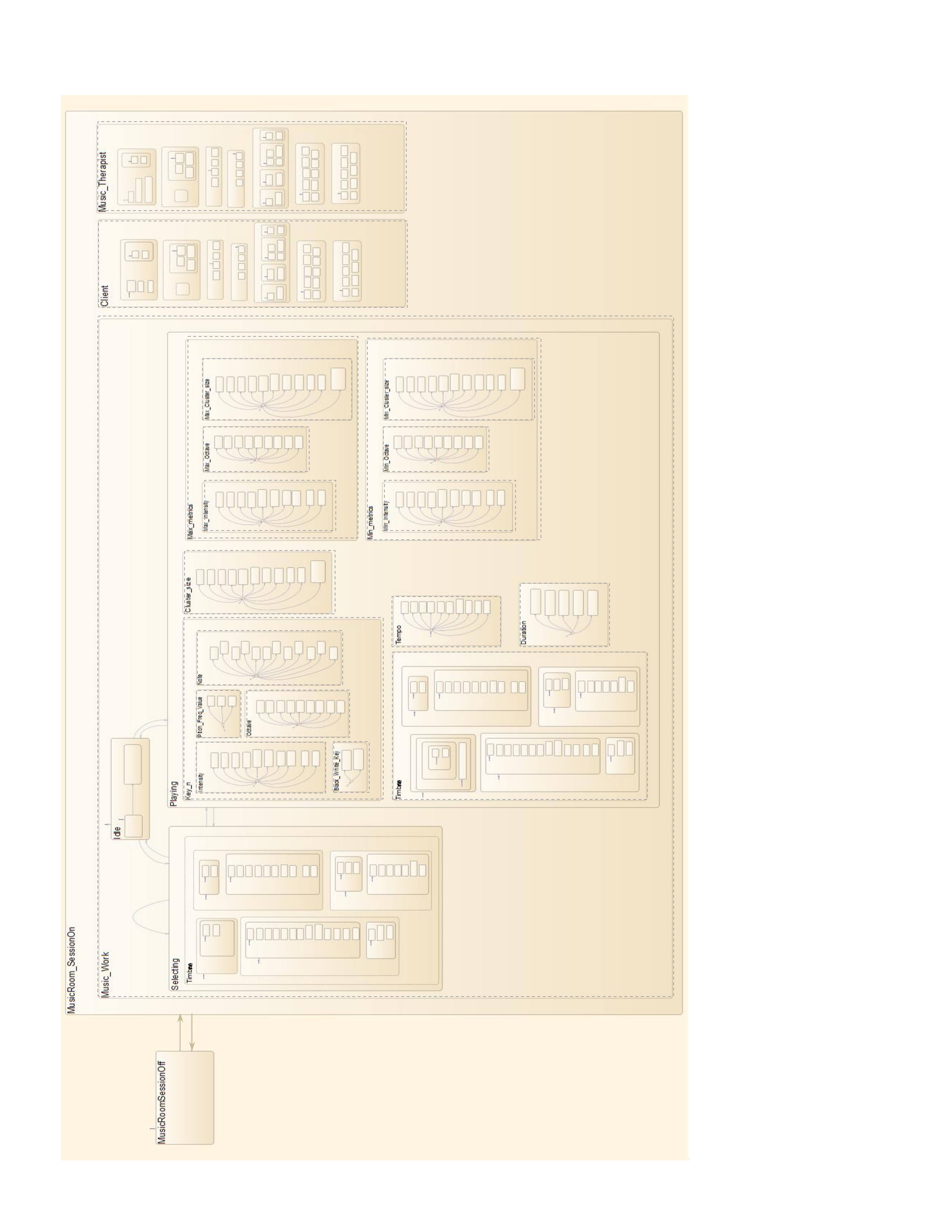

Supplement: S4 Fig — The Statecharts visual formalism [44] modeling the music room and three concurrent/orthogonal states (dashed lines) specifying the entities therein: the Music_work, Client (patient) and Music_Therapist. The figure also shows the events that trigger the beginning of the therapy session and its termination, specified as mutually exclusive states, MusicRoomSessionOn and MusicRoomSessionOff, respectively (with solid lines). The Music_work creator can be in one of three states: Playing (see also S5 Fig), musical instrument Selecting or Idle. (TIF) [file pone.0213247.s004.tif]

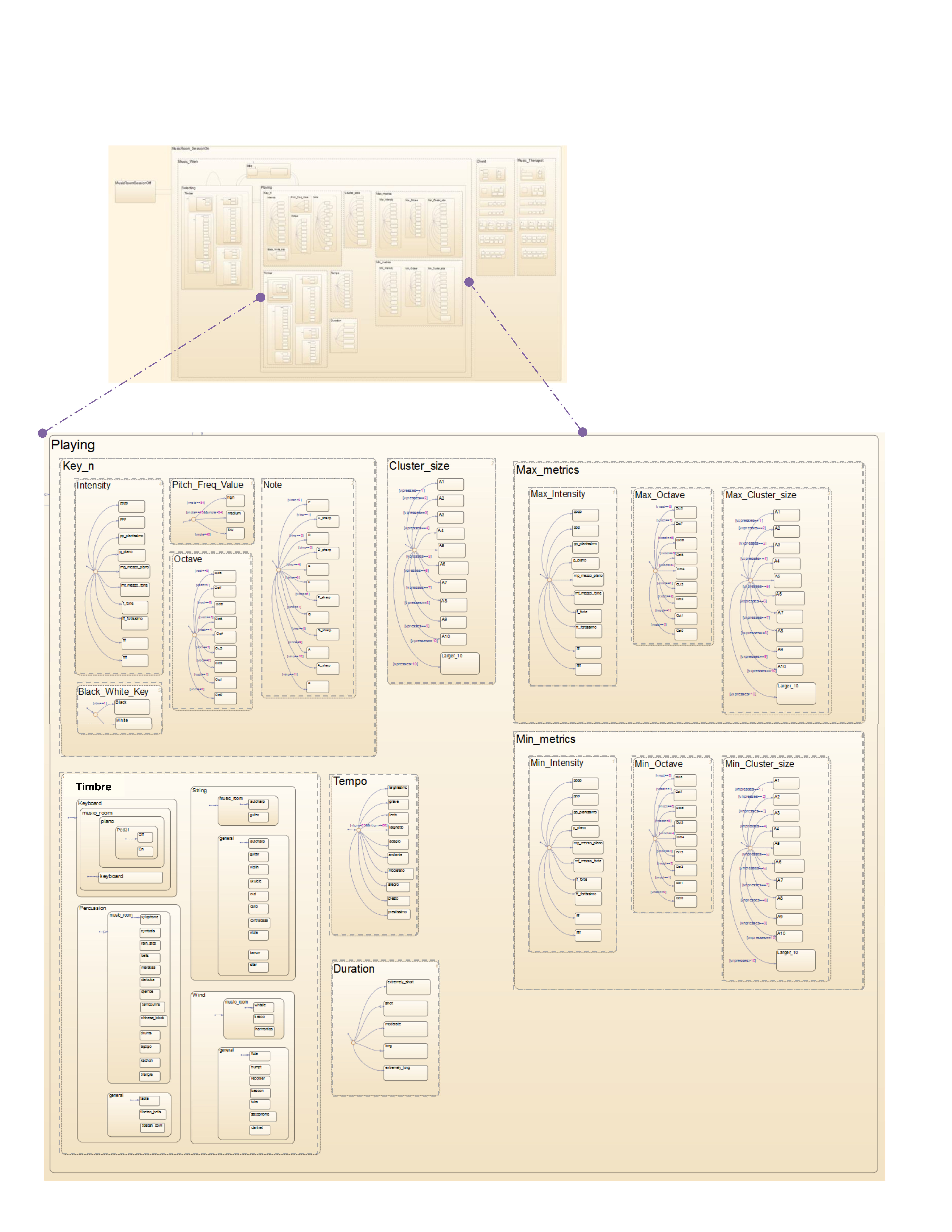

Supplement: S5 Fig — The visual modeling of the system using the Statecharts formalism. (Top Panel) The top view of the system, see S4 Fig. (Bottom Panel) The Playing state, zoomed in, is further decomposed into sub-states formulating the music making process. (TIF) [file pone.0213247.s005.tif]

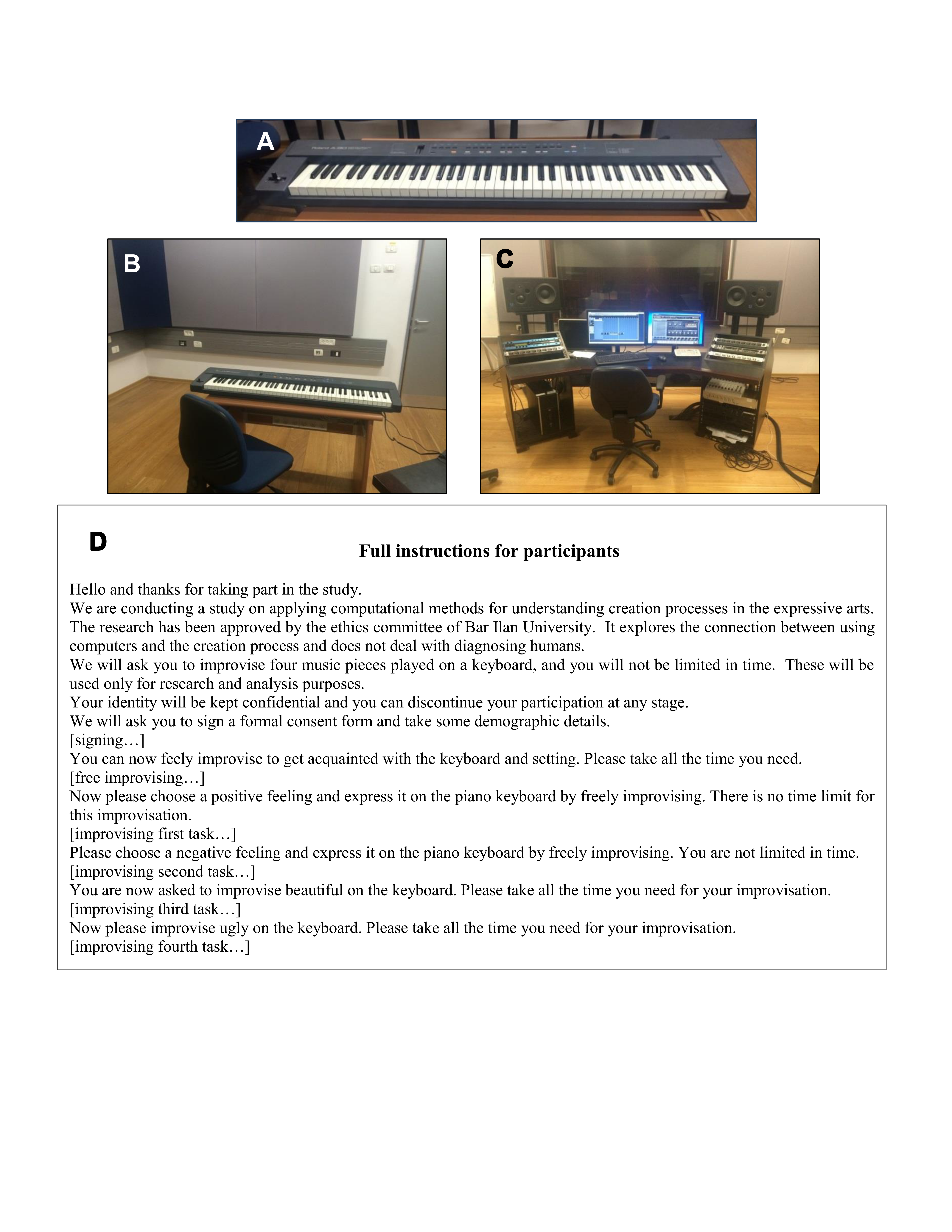

Supplement: S6 Fig — The study was carried out in the recording studio in the Music Department of Bar Ilan University. (A) The apparatus—Roland A-30-MIDI keyboard controller, comprised of 76 keys, 31 of them black and 45 white. Piano sound. The auditory feedback through speakers. (B) The participant playing station. (C) The experimenter control station. (D) The instructions for the participants given by the experimenter. (TIF) [file pone.0213247.s006.tif]
