# Supplementary material for: Computational elucidation of the effects induced by music making
Source: PLoS One. 2019 Mar 7;14(3):e0213247. doi: 10.1371/journal.pone.0213247 (PMC6405055; doi:10.1371/journal.pone.0213247)
Supplement: S1 Table — (PDF) [file pone.0213247.s007.pdf]

Attribute	*Parameter*	*Young*	*Old*	*Beautiful & Positive*	*Ugly & Negative*
**Time**	% playing time	69.6%	67.5%	69.2%	67.4%
	% idle time	30.4%	32.5%	30.8%	32.6%
	% start time	16.6%	19.5%*	16.9%	19.3%
	% concurrent	224.6%	291%***	196.1%	309.5%***
	total (minutes)	0.83	0.88	0.8	0.8
**Notes/ Keys**	# of presses	182.7	142.5	119.9	189.0
	% used	27.3%	30.4%*	25.5%	31.3%**
	presses per key	6.2	5.7	5.3	6.4
	play per key (sec)	0.38	0.41	0.39	0.4
	% black presses	12.2%	19.7%***	11%	20.5%***
	% white presses	87.8%***	80.3%	89%***	79.5%
**Intensity**†	average	6.0	6.0	5.7	6.4***
	lowest (minimum)	2.3	2.4	2.2	2.5
	highest (maximum)	8.2	8.4*	7.9	8.4***
	most used	6.4	6.3	6	6.8***
**Octave**	average	3.6	3.6	4.3***	2.9
	lowest (minimum)	2.3	2.2	3***	1.5
	highest (maximum)	5.3	5.3	5.7***	4.8
	most used	3.5	3.6	4.3***	2.8
**Cluster of notes**	# of instances	337.9	264	216.5	352.7
	max pressed‡	5.1	6.4***	4.2	6.9***
	most pressed§	2.0	2.7***	1.8	2.7***
	% most played$^{\|}$	58.2%***	53.2%	63.1%***	50.6
**Transit-ions**	% diminuendo	46.9%	46.6%	46.8%	46.6%
	% crescendo	49.6%	49.7%	49.9%	49.6%
	% same intensity	3.5%	3.8%	3.3%	3.8%
	% accelerando	10.4%	11.7%	11.4%	10.3%
	% ritardando	89.6%	88.3%	88.6%	89.7%
	% white to black	7.3%	10.8%***	5.9%	11.7%***
	% black to white	7.1%	10.6%***	5.8%	11.5%***
	% black to black	4.9%	8.9%***	5.1%	8.8%***
	% white to white	80.7%***	69.7%	83.2%***	68.0%

* $p < 0.05$; ** $p < 0.01$; *** $p < 0.001$

† 1-*pppp* ; 2-*ppp* ; 3-*pp* ; 4-*p* ; 5-*mp* ; 6-*mf* ; 7-*f* ; 8-*ff* ; 9-*fff* ; 10-*ffff*

‡ configuration of maximum number of keys pressed ;

§ most pressed configuration ;

$^{\|}$ relative playing time of the most pressed configuration ;
