# Supplementary material for: Computational elucidation of the effects induced by music making
Source: PLoS One. 2019 Mar 7;14(3):e0213247. doi: 10.1371/journal.pone.0213247 (PMC6405055; doi:10.1371/journal.pone.0213247)
Supplement: S2 File — (PDF) [file pone.0213247.s011.pdf]

Tasks Study (Ugly, Beautiful, Negative, Positive)- MATLAB Anova Results

Attribute	*Parameter*	Column# in results
**Time**	% playing time	2
	% idle time	4
	% start time	46
	% concurrent	3
	total (minutes)	1
**Notes/ Keys**	# of presses	10
	% used	11
	presses per key	29
	play per key (sec)	12
	% black presses	34
	% white presses	35
**Intensity**†	average	9
	lowest (minimum)	7
	highest (maximum)	8
	most used	5
**Octave**	average	21
	lowest (minimum)	19
	highest (maximum)	20
	most used	17
**Cluster of notes**	# of instances	24
	max pressed‡	28
	most pressed§	25
	% most played‖	26
**Transit-ions**	% diminuendo	41
	% crescendo	42
	% same intensity	43
	% accelerando	44
	% ritardando	45
	% white to black	37
	% black to white	38
	% black to black	39
	% white to white	40
**Pitch classes**	% playing time: C,C#,D,D#,E,F,F#, G,G#,A,A#,B	48-59

**Variable**	**Definition**
Parameters matrix representation ***(pages 2-8)***	
U	Ugly task mean
USD	Ugly task SD
USE	Ugly task SEM
B	Beautiful task mean
BSD	Beautiful task SD
BSE	Beautiful task SEM
N	Negative task mean
NSD	Negative task SD
NSE	Negative task SEM
P	Positive task mean
PSD	Positive task SD
PSE	Positive task SEM
PP	*p*-value is the probability that the F-statistic can take a value larger than the computed test-statistic value. Anova tests the null hypothesis that all tasks means are equal to each other against the alternative hypothesis that at least one tasks mean is different from the others.
η^2	Effect size
Per parameter description ***(see page 9 onwards)***	
F (in anova table)	F-statistic, which is the ratio of the mean squares.
df (in anova table)	The degrees of freedom associated with each source.
multiple comparison (Bonferroni) results table	The first two columns show which groups are compared. The fourth column shows the mean parameter difference for the compared groups. The third and fifth columns show the lower and upper limits for a 95% confidence interval for the difference in the group means. The last column shows the p-values for the tests.
means table	mean estimates for each group of tasks

https://www.mathworks.com/help/stats/anova1.html
https://www.mathworks.com/help/stats/multiple-comparisons.html

† 1-*pppp* ; 2-*ppp* ; 3-*pp* ; 4-*p* ; 5-*mp* ; 6-*mf* ; 7-*f* ; 8-*ff* ; 9-*fff* ; 10-*ffff* (See Fig 4A)
‡ configuration of maximum number of keys pressed ; § most pressed configuration ;
‖ relative playing time of the most pressed configuration;

U =

Columns 1 through 10

0.7304	62.6843	352.9343	37.3157	6.9902	39.2755	2.5294	8.8333	6.5882	254.9412

Columns 11 through 20

36.8912	0.2702	572.4137	51.3137	2.8627	15.2480	2.8725	50.1206	1.5196	5.0784

Columns 21 through 30

3.0882	4.6078	21.9598	475.6373	2.8431	46.7059	1.0000	8.1961	7.3167	27.4873

Columns 31 through 40

25.3686	43.3725	74.6324	22.9520	77.0510	0.3882	12.2912	12.0020	10.6598	65.0588

Columns 41 through 50

47.3382	48.7794	3.8814	7.2873	92.7127	22.2010	59.5716	13.0373	3.8186	11.5235

Columns 51 through 60

3.2118	11.1784	10.7627	4.0500	10.9049	4.1755	11.7637	4.2961	11.2735	NaN

B =

Columns 1 through 10

0.8804	70.8402	200.3980	29.1598	5.5980	39.2882	2.0294	7.6176	5.3431	117.8922

Columns 11 through 20

24.9863	0.4483	180.6922	67.6373	4.2745	19.7000	4.2157	56.1500	3.0098	5.6667

Columns 21 through 30

4.2745	3.8431	28.0275	215.8824	1.9510	64.5735	1.0000	4.0882	4.9863	12.2118

Columns 31 through 40

15.7324	33.7931	84.2686	13.1735	86.8265	0.1824	6.9196	6.6529	6.2549	80.1765

Columns 41 through 50

46.3961	50.0882	3.5127	12.9549	86.9990	16.8167	63.8471	17.0961	1.7588	13.1873

Columns 51 through 60

3.1971	12.6186	11.4784	2.4412	13.6363	2.5608	10.7971	2.6392	8.5706	NaN

```
N =

  Columns 1 through 10

    0.8618   72.0833  266.0382   27.9167    6.6078   41.0902    2.3824    8.4608    6.1765  123.1373

  Columns 11 through 20

   25.6882    0.5276  290.3980   48.2941    2.6275   20.8363    2.6569   58.9637    1.5098    4.5784

  Columns 21 through 30

    2.7647    4.2353   28.3147  229.8431    2.4804   54.5324    1.0000    5.6667    5.5814   15.7510

  Columns 31 through 40

   20.3961   32.5235   79.6049   18.0559   81.9441    0.4314   11.1157   10.9373    6.9382   71.0049

  Columns 41 through 50

   45.8422   50.3824    3.7784   13.3578   86.6235   16.4235   62.5529   14.9637    2.7892   10.0529

  Columns 51 through 60

    3.8088   13.3647   12.1245    3.9069   12.4392    2.9049   10.3059    3.4922    9.8529       NaN

P =

  Columns 1 through 10

    0.7196   67.5853  191.8814   32.4147    6.3529   40.7000    2.4314    8.2059    6.0490  121.8529

  Columns 11 through 20

   26.0304    0.3329  293.0422   67.8725    4.2941   19.4892    4.4020   55.6167    2.9118    5.7647

  Columns 21 through 30

    4.3137    4.0294   26.9284  217.1961    1.6961   61.6284    1.0000    4.3725    5.5961   11.0098

  Columns 31 through 40

   12.4108   36.3814   87.5931    8.8196   91.1804    0.1108    4.9598    4.9461    3.8608   86.2373

  Columns 41 through 50

   47.2745   49.7265    3.0069    9.8480   90.1539   17.0157   58.0637   16.3373    1.5216   12.7059

  Columns 51 through 60

    1.3853   13.4324   12.0735    1.8471   15.2304    1.4765   12.4382    1.4461   10.1029       NaN

>>
```

PP =

Columns 1 through 10

0.6335 0.0081 0.0000 0.0081 0.0000 0.6067 0.1013 0.0000 0.0000 0.0431

Columns 11 through 20

0.0000 0.0000 0.0000 0.0000 0.0000 0.0028 0.0000 0.0062 0.0000 0.0000

Columns 21 through 30

0.0000 0.3700 0.0001 0.0462 0.0000 0.0000 NaN 0.0000 0.3040 0.0000

Columns 31 through 40

0.0000 0.0012 0.0000 0.0000 0.0000 0.0063 0.0000 0.0000 0.0027 0.0000

Columns 41 through 50

0.1987 0.2788 0.2479 0.0000 0.0000 0.0407 0.5083 0.1140 0.0007 0.0380

Columns 51 through 59

0.0189 0.2222 0.7075 0.0050 0.0042 0.0018 0.2571 0.0008 0.1283

Eta-sqaure =

Columns 1 through 10

[0.0042] [0.0287] [0.1268] [0.0287] [0.0985] [0.0045] [0.0153] [0.1217] [0.1132] [0.0199]

Columns 11 through 20

[0.0681] [0.0730] [0.0865] [0.2784] [0.2837] [0.0342] [0.3094] [0.0302] [0.2978] [0.1168]

Columns 21 through 30

[0.3388] [0.0077] [0.0489] [0.0196] [0.0572] [0.0725] [NaN] [0.1637] [0.0089] [0.0903]

Columns 31 through 40

[0.0636] [0.0386] [0.0636] [0.0711] [0.0711] [0.0301] [0.0808] [0.0791] [0.0344] [0.0808]

Columns 41 through 30

[0.0114] [0.0095] [0.0102] [0.0583] [0.0586] [0.0202] [0.0057] [0.0146] [0.0411] [0.0206]

Columns 51 through 59

[0.0243] [0.0108] [0.0034] [0.0312] [0.0321] [0.0366] [0.0099] [0.0408] [0.0139]

USD =

1.0e+03 *

Columns 1 through 10

0.0010 0.0201 0.2439 0.0201 0.0016 0.0119 0.0018 0.0012 0.0013 0.7398

Columns 11 through 20

0.0232 0.0002 0.8062 0.0156 0.0014 0.0102 0.0012 0.0167 0.0009 0.0015

Columns 21 through 30

0.0009 0.0032 0.0098 1.4195 0.0022 0.0239 0 0.0057 0.0158 0.0265

Columns 31 through 40

0.0180 0.0243 0.0180 0.0190 0.0190 0.0004 0.0110 0.0109 0.0106 0.0290

Columns 41 through 50

0.0057 0.0058 0.0036 0.0080 0.0080 0.0179 0.0290 0.0095 0.0042 0.0078

Columns 51 through 60

0.0039 0.0078 0.0081 0.0044 0.0068 0.0054 0.0074 0.0056 0.0075 NaN

USE =

Columns 1 through 10

0.0974 1.9938 24.1496 1.9938 0.1548 1.1807 0.1761 0.1175 0.1280 73.2536

Columns 11 through 20

2.2981 0.0217 79.8264 1.5413 0.1365 1.0057 0.1228 1.6531 0.0844 0.1505

Columns 21 through 30

0.0860 0.3178 0.9745 140.5472 0.2189 2.3622 0 0.5620 1.5684 2.6230

Columns 31 through 40

1.7784 2.4032 1.7785 1.8818 1.8817 0.0384 1.0908 1.0762 1.0519 2.8709

Columns 41 through 50

0.5655 0.5736 0.3591 0.7873 0.7873 1.7715 2.8678 0.9373 0.4132 0.7707

Columns 51 through 60

0.3832 0.7694 0.7987 0.4333 0.6744 0.5362 0.7310 0.5580 0.7461 NaN

BSD =

Columns 1 through 10

1.3496	21.3076	108.8513	21.3076	1.5937	13.3318	1.2700	1.1261	1.1301	253.4949

Columns 11 through 20

15.9158	0.2396	109.7490	14.5110	1.2118	11.8091	1.1400	18.3101	1.4316	1.0082

Columns 21 through 30

0.9765	3.2235	10.6154	487.0401	1.3815	27.4064	0	2.1531	6.2212	17.4566

Columns 31 through 40

20.4810	20.1192	20.4809	21.1713	21.1713	0.4239	9.5532	9.2610	16.0886	28.2563

Columns 41 through 50

6.0140	5.7744	3.1981	10.8862	10.9844	15.8787	31.2393	14.2146	4.2041	7.8457

Columns 51 through 60

7.5465	7.1746	8.0676	6.0930	9.0528	5.8873	8.6613	5.2641	6.9623	NaN

BSE =

Columns 1 through 10

0.1336	2.1098	10.7779	2.1098	0.1578	1.3200	0.1258	0.1115	0.1119	25.0997

Columns 11 through 20

1.5759	0.0237	10.8668	1.4368	0.1200	1.1693	0.1129	1.8130	0.1417	0.0998

Columns 21 through 30

0.0967	0.3192	1.0511	48.2242	0.1368	2.7136	0	0.2132	0.6160	1.7285

Columns 31 through 40

2.0279	1.9921	2.0279	2.0963	2.0963	0.0420	0.9459	0.9170	1.5930	2.7978

Columns 41 through 50

0.5955	0.5718	0.3167	1.0779	1.0876	1.5722	3.0931	1.4075	0.4163	0.7768

Columns 51 through 60

0.7472	0.7104	0.7988	0.6033	0.8964	0.5829	0.8576	0.5212	0.6894	NaN

NSD =

Columns 1 through 10

1.3614	20.1977	192.8303	20.1977	1.6837	12.9885	1.5028	1.2717	1.4171	185.9623

Columns 11 through 20

18.4790	0.5991	419.8788	14.6467	1.2342	13.1345	1.2308	19.7170	0.8052	1.6735

Columns 21 through 30

1.0733	3.2064	14.5415	360.5857	2.3661	23.7293	0	3.7000	5.2217	20.2102

Columns 31 through 40

19.5445	21.2361	19.5433	20.1908	20.1908	1.4011	11.8762	11.8162	12.6471	30.5043

Columns 41 through 50

6.9754	7.8332	3.7417	10.5389	10.5566	15.9902	30.8011	15.5558	4.9976	9.0590

Columns 51 through 60

7.1720	11.8647	10.8870	6.3714	8.6052	4.8159	9.3309	5.8439	8.4108	NaN

NSE =

Columns 1 through 10

0.1348	1.9999	19.0930	1.9999	0.1667	1.2860	0.1488	0.1259	0.1403	18.4130

Columns 11 through 20

1.8297	0.0593	41.5742	1.4502	0.1222	1.3005	0.1219	1.9523	0.0797	0.1657

Columns 21 through 30

0.1063	0.3175	1.4398	35.7033	0.2343	2.3496	0	0.3664	0.5170	2.0011

Columns 31 through 40

1.9352	2.1027	1.9351	1.9992	1.9992	0.1387	1.1759	1.1700	1.2523	3.0204

Columns 41 through 50

0.6907	0.7756	0.3705	1.0435	1.0453	1.5833	3.0498	1.5403	0.4948	0.8970

Columns 51 through 60

0.7101	1.1748	1.0780	0.6309	0.8520	0.4768	0.9239	0.5786	0.8328	NaN

```
PSD =

  Columns 1 through 10

    0.6730   23.1584   85.9609   23.1584    1.3249   10.2646    1.4992    1.1885    1.1889  151.6445

  Columns 11 through 20

   14.2566    0.2158  238.4786   13.7212    1.1043    9.9987    1.0555   18.1649    1.2516    0.9353

  Columns 21 through 30

    0.9645    3.2775    9.7691  293.5891    0.9728   24.1309         0    1.9495    4.7539   17.6498

  Columns 31 through 40

   17.2160   18.0353   17.2153   16.0682   16.0682    0.2803    7.6221    7.6268   11.9235   22.1308

  Columns 41 through 50

    4.3629    5.0804    2.7802   10.2795   10.2802   16.4214   30.8482   10.7326    4.2671    8.7586

  Columns 51 through 60

    3.2494    7.1936   10.3573    3.7987   10.1922    3.3924    7.7414    3.5094    9.3473       NaN


PSE =

  Columns 1 through 10

    0.0666    2.2930    8.5114    2.2930    0.1312    1.0163    0.1484    0.1177    0.1177   15.0150

  Columns 11 through 20

    1.4116    0.0214   23.6129    1.3586    0.1093    0.9900    0.1045    1.7986    0.1239    0.0926

  Columns 21 through 30

    0.0955    0.3245    0.9673   29.0697    0.0963    2.3893         0    0.1930    0.4707    1.7476

  Columns 31 through 40

    1.7046    1.7858    1.7046    1.5910    1.5910    0.0278    0.7547    0.7552    1.1806    2.1913

  Columns 41 through 50

    0.4320    0.5030    0.2753    1.0178    1.0179    1.6260    3.0544    1.0627    0.4225    0.8672

  Columns 51 through 60

    0.3217    0.7123    1.0255    0.3761    1.0092    0.3359    0.7665    0.3475    0.9255       NaN

>>
```

Explanation of output format

(See https://www.mathworks.com/help/stats/anova1.html ;
https://www.mathworks.com/help/stats/multiple-comparisons.html)

34 parameter/column number (see table on first page)

Anova Table	Definition
source	The source of the variability
SS	The sum of squares due to each source
df	The degrees of freedom associated with each source.
MS	The mean squares for each source; SS/df
F	F-statistic, which is the ratio of the mean squares.
Prob>F	The *p* value probability that the F statisticcan take a value larget than the computed test-statistic value.
Columns	Variability between groups
Error	Variability within groups
Total	Total variability

'Source'	'SS'	'df'	'MS'	'F'	'Prob>F'
'Columns'	[1.1409e+04]	[3]	[3.8030e+03]	[10.3114]	[1.4886e-06]
'Error'	[1.4900e+05]	[404]	[368.8187]	[]	[]
'Total'	[1.6041e+05]	[407]	[]	[]	[]

1.0000	2.0000	2.6485	9.7784	16.9083	0.0019
1.0000	3.0000	-2.2338	4.8961	12.0260	0.4164
1.0000	4.0000	7.0025	14.1324	21.2622	0.0000
2.0000	3.0000	-12.0122	-4.8824	2.2475	0.4211
2.0000	4.0000	-2.7760	4.3539	11.4838	0.6373
3.0000	4.0000	2.1064	9.2363	16.3662	0.0039

Multiple comparison (Bonferroni) results table
The first two columns show which groups are compared. The fourth column shows the mean parameter difference for the compared groups. The third and fifth columns show the lower and upper limits for a 95% confidence interval for the difference in the group means. The last column shows the p-values for the tests

22.9520	1.9015
13.1735	1.9015
18.0559	1.9015
8.8196	1.9015

Means table
mean estimates for each group of tasks, and standard errors of the estimates

---

1

'Source'	'SS'	'df'	'MS'	'F'	'Prob>F'
'Columns'	[2.1867]	[3]	[0.7289]	[0.5723]	[0.6335]
'Error'	[514.5821]	[404]	[1.2737]	[]	[]
'Total'	[516.7688]	[407]	[]	[]	[]

1.0000	2.0000	-0.5680	-0.1490	0.2700	1.0000
1.0000	3.0000	-0.5494	-0.1304	0.2886	1.0000
1.0000	4.0000	-0.4072	0.0118	0.4308	1.0000
2.0000	3.0000	-0.4004	0.0186	0.4376	1.0000
2.0000	4.0000	-0.2582	0.1608	0.5798	1.0000
3.0000	4.0000	-0.2768	0.1422	0.5612	1.0000

0.7314	0.1117
0.8804	0.1117
0.8618	0.1117
0.7196	0.1117

2

'Source'	'SS'	'df'	'MS'	'F'	'Prob>F'
'Columns'	[5.3869e+03]	[3]	[1.7956e+03]	[3.9820]	[0.0081]
'Error'	[1.8218e+05]	[404]	[450.9357]	[]	[]
'Total'	[1.8756e+05]	[407]	[]	[]	[]

1.0000	2.0000	-16.0396	-8.1559	-0.2721	0.0382
1.0000	3.0000	-17.2828	-9.3990	-1.5153	0.0101
1.0000	4.0000	-12.7847	-4.9010	2.9828	0.6005
2.0000	3.0000	-9.1269	-1.2431	6.6406	1.0000
2.0000	4.0000	-4.6289	3.2549	11.1387	1.0000
3.0000	4.0000	-3.3857	4.4980	12.3818	0.7868

62.6843	2.1026
70.8402	2.1026
72.0833	2.1026
67.5853	2.1026

3

'Source'	'SS'	'df'	'MS'	'F'	'Prob>F'
'Columns'	[1.6992e+06]	[3]	[5.6641e+05]	[19.5469]	[7.4884e-12]
'Error'	[1.1707e+07]	[404]	[2.8977e+04]	[]	[]
'Total'	[1.3406e+07]	[407]	[]	[]	[]

1.0000	2.0000	89.3383	152.5363	215.7343	0.0000
1.0000	3.0000	23.6981	86.8961	150.0941	0.0018
1.0000	4.0000	97.8549	161.0529	224.2509	0.0000
2.0000	3.0000	-128.8382	-65.6402	-2.4422	0.0369
2.0000	4.0000	-54.6813	8.5167	71.7147	1.0000
3.0000	4.0000	10.9589	74.1569	137.3549	0.0120

352.9343	16.8549
200.3980	16.8549
266.0382	16.8549
191.8814	16.8549

4

'Source'	'SS'	'df'	'MS'	'F'	'Prob>F'
'Columns'	[5.3869e+03]	[3]	[1.7956e+03]	[3.9820]	[0.0081]
'Error'	[1.8218e+05]	[404]	[450.9357]	[]	[]
'Total'	[1.8756e+05]	[407]	[]	[]	[]

1.0000	2.0000	0.2721	8.1559	16.0396	0.0382
1.0000	3.0000	1.5153	9.3990	17.2828	0.0101
1.0000	4.0000	-2.9828	4.9010	12.7847	0.6005
2.0000	3.0000	-6.6406	1.2431	9.1269	1.0000
2.0000	4.0000	-11.1387	-3.2549	4.6289	1.0000
3.0000	4.0000	-12.3818	-4.4980	3.3857	0.7868

37.3157	2.1026
29.1598	2.1026
27.9167	2.1026
32.4147	2.1026

5

'Source'	'SS'	'df'	'MS'	'F'	'Prob>F'
'Columns'	[105.6961]	[3]	[35.2320]	[14.7177]	[4.1029e-09]
'Error'	[967.1176]	[404]	[2.3939]	[]	[]
'Total'	[1.0728e+03]	[407]	[]	[]	[]

1.0000	2.0000	0.8177	1.3922	1.9666	0.0000
1.0000	3.0000	-0.1921	0.3824	0.9568	0.4701
1.0000	4.0000	0.0628	0.6373	1.2117	0.0207
2.0000	3.0000	-1.5842	-1.0098	-0.4354	0.0000
2.0000	4.0000	-1.3293	-0.7549	-0.1805	0.0033
3.0000	4.0000	-0.3195	0.2549	0.8293	1.0000

6.9902	0.1532
5.5980	0.1532
6.6078	0.1532
6.3529	0.1532

6

'Source'	'SS'	'df'	'MS'	'F'	'Prob>F'
'Columns'	[273.2311]	[3]	[91.0770]	[0.6133]	[0.6067]
'Error'	[5.9993e+04]	[404]	[148.4972]	[]	[]
'Total'	[6.0266e+04]	[407]	[]	[]	[]

1.0000	2.0000	-4.5369	-0.0127	4.5114	1.0000
1.0000	3.0000	-6.3388	-1.8147	2.7094	1.0000
1.0000	4.0000	-5.9486	-1.4245	3.0996	1.0000
2.0000	3.0000	-6.3261	-1.8020	2.7222	1.0000
2.0000	4.0000	-5.9359	-1.4118	3.1124	1.0000
3.0000	4.0000	-4.1339	0.3902	4.9143	1.0000

39.2755	1.2066
39.2882	1.2066
41.0902	1.2066
40.7000	1.2066

7

'Source'	'SS'	'df'	'MS'	'F'	'Prob>F'
'Columns'	[14.5294]	[3]	[4.8431]	[2.0872]	[0.1013]
'Error'	[937.4314]	[404]	[2.3204]	[]	[]

'Total'	[951.9608]	[407]	[]	[]	[]

1.0000	2.0000	-0.0655	0.5000	1.0655	0.1173
1.0000	3.0000	-0.4185	0.1471	0.7126	1.0000
1.0000	4.0000	-0.4675	0.0980	0.6636	1.0000
2.0000	3.0000	-0.9185	-0.3529	0.2126	0.5926
2.0000	4.0000	-0.9675	-0.4020	0.1636	0.3613
3.0000	4.0000	-0.6145	-0.0490	0.5165	1.0000

2.5294	0.1508
2.0294	0.1508
2.3824	0.1508
2.4314	0.1508

8

'Source'	'SS'	'df'	'MS'	'F'	'Prob>F'
'Columns'	[79.8725]	[3]	[26.6242]	[18.6650]	[2.3391e-11]
'Error'	[576.2745]	[404]	[1.4264]	[]	[]
'Total'	[656.1471]	[407]	[]	[]	[]

1.0000	2.0000	0.7723	1.2157	1.6591	0.0000
1.0000	3.0000	-0.0709	0.3725	0.8160	0.1587
1.0000	4.0000	0.1840	0.6275	1.0709	0.0012
2.0000	3.0000	-1.2865	-0.8431	-0.3997	0.0000
2.0000	4.0000	-1.0316	-0.5882	-0.1448	0.0029
3.0000	4.0000	-0.1885	0.2549	0.6983	0.7695

8.8333	0.1183
7.6176	0.1183
8.4608	0.1183
8.2059	0.1183

9

'Source'	'SS'	'df'	'MS'	'F'	'Prob>F'
'Columns'	[82.0980]	[3]	[27.3660]	[17.1869]	[1.5984e-10]
'Error'	[643.2745]	[404]	[1.5923]	[]	[]
'Total'	[725.3725]	[407]	[]	[]	[]

1.0000	2.0000	0.7766	1.2451	1.7136	0.0000
1.0000	3.0000	-0.0567	0.4118	0.8802	0.1217
1.0000	4.0000	0.0707	0.5392	1.0077	0.0146
2.0000	3.0000	-1.3018	-0.8333	-0.3649	0.0000
2.0000	4.0000	-1.1744	-0.7059	-0.2374	0.0005
3.0000	4.0000	-0.3410	0.1275	0.5959	1.0000

6.5882	0.1249
5.3431	0.1249
6.1765	0.1249
6.0490	0.1249

10

'Source'	'SS'	'df'	'MS'	'F'	'Prob>F'
'Columns'	[1.3748e+06]	[3]	[4.5825e+05]	[2.7392]	[0.0431]
'Error'	[6.7587e+07]	[404]	[1.6729e+05]	[]	[]
'Total'	[6.8962e+07]	[407]	[]	[]	[]

1.0000	2.0000	-14.8018	137.0490	288.8998	0.1030

1.0000	3.0000	-20.0469	131.8039	283.6547	0.1313
1.0000	4.0000	-18.7626	133.0882	284.9391	0.1238
2.0000	3.0000	-157.0959	-5.2451	146.6057	1.0000
2.0000	4.0000	-155.8116	-3.9608	147.8900	1.0000
3.0000	4.0000	-150.5665	1.2843	153.1351	1.0000

254.9412	40.4987
117.8922	40.4987
123.1373	40.4987
121.8529	40.4987

11

'Source'	'SS'	'df'	'MS'	'F'	'Prob>F'
'Columns'	[9.8657e+03]	[3]	[3.2886e+03]	[9.8406]	[2.8138e-06]
'Error'	[1.3501e+05]	[404]	[334.1828]	[]	[]
'Total'	[1.4488e+05]	[407]	[]	[]	[]

1.0000	2.0000	5.1181	11.9049	18.6917	0.0000
1.0000	3.0000	4.4161	11.2029	17.9898	0.0001
1.0000	4.0000	4.0739	10.8608	17.6476	0.0002
2.0000	3.0000	-7.4888	-0.7020	6.0849	1.0000
2.0000	4.0000	-7.8310	-1.0441	5.7427	1.0000
3.0000	4.0000	-7.1290	-0.3422	6.4447	1.0000

36.8912	1.8101
24.9863	1.8101
25.6882	1.8101
26.0304	1.8101

12

'Source'	'SS'	'df'	'MS'	'F'	'Prob>F'
'Columns'	[4.0638]	[3]	[1.3546]	[10.6024]	[1.0049e-06]
'Error'	[51.6168]	[404]	[0.1278]	[]	[]
'Total'	[55.6806]	[407]	[]	[]	[]

1.0000	2.0000	-0.3107	-0.1780	-0.0453	0.0025
1.0000	3.0000	-0.3901	-0.2574	-0.1246	0.0000
1.0000	4.0000	-0.1953	-0.0626	0.0701	1.0000
2.0000	3.0000	-0.2120	-0.0793	0.0534	0.6825
2.0000	4.0000	-0.0173	0.1154	0.2481	0.1299
3.0000	4.0000	0.0620	0.1947	0.3274	0.0007

0.2702	0.0354
0.4483	0.0354
0.5276	0.0354
0.3329	0.0354

13

'Source'	'SS'	'df'	'MS'	'F'	'Prob>F'
'Columns'	[8.5601e+06]	[3]	[2.8534e+06]	[12.7499]	[5.6168e-08]
'Error'	[9.0414e+07]	[404]	[2.2380e+05]	[]	[]
'Total'	[9.8974e+07]	[407]	[]	[]	[]

1.0000	2.0000	216.0901	391.7216	567.3531	0.0000
1.0000	3.0000	106.3842	282.0157	457.6472	0.0002
1.0000	4.0000	103.7401	279.3716	455.0031	0.0002
2.0000	3.0000	-285.3374	-109.7059	65.9256	0.5909

2.0000	4.0000	-287.9815	-112.3500	63.2815	0.5439
3.0000	4.0000	-178.2756	-2.6441	172.9874	1.0000

572.4137	46.8410
180.6922	46.8410
290.3980	46.8410
293.0422	46.8410

14

'Source'	'SS'	'df'	'MS'	'F'	'Prob>F'
'Columns'	[3.3336e+04]	[3]	[1.1112e+04]	[51.9458]	[2.0490e-28]
'Error'	[8.6422e+04]	[404]	[213.9160]	[]	[]
'Total'	[1.1976e+05]	[407]	[]	[]	[]

1.0000	2.0000	-21.7535	-16.3235	-10.8936	0.0000
1.0000	3.0000	-2.4104	3.0196	8.4496	0.8469
1.0000	4.0000	-21.9888	-16.5588	-11.1289	0.0000
2.0000	3.0000	13.9132	19.3431	24.7731	0.0000
2.0000	4.0000	-5.6653	-0.2353	5.1947	1.0000
3.0000	4.0000	-25.0084	-19.5784	-14.1485	0.0000

51.3137	1.4482
67.6373	1.4482
48.2941	1.4482
67.8725	1.4482

15

'Source'	'SS'	'df'	'MS'	'F'	'Prob>F'
'Columns'	[244.5000]	[3]	[81.5000]	[53.3291]	[4.6522e-29]
'Error'	[617.4118]	[404]	[1.5282]	[]	[]
'Total'	[861.9118]	[407]	[]	[]	[]

1.0000	2.0000	-1.8707	-1.4118	-0.9528	0.0000
1.0000	3.0000	-0.2237	0.2353	0.6943	1.0000
1.0000	4.0000	-1.8903	-1.4314	-0.9724	0.0000
2.0000	3.0000	1.1881	1.6471	2.1060	0.0000
2.0000	4.0000	-0.4786	-0.0196	0.4393	1.0000
3.0000	4.0000	-2.1256	-1.6667	-1.2077	0.0000

2.8627	0.1224
4.2745	0.1224
2.6275	0.1224
4.2941	0.1224

16

'Source'	'SS'	'df'	'MS'	'F'	'Prob>F'
'Columns'	[1.8407e+03]	[3]	[613.5812]	[4.7647]	[0.0028]
'Error'	[5.2026e+04]	[404]	[128.7771]	[]	[]
'Total'	[5.3867e+04]	[407]	[]	[]	[]

1.0000	2.0000	-8.6650	-4.4520	-0.2389	0.0320
1.0000	3.0000	-9.8013	-5.5882	-1.3752	0.0029
1.0000	4.0000	-8.4542	-4.2412	-0.0281	0.0475
2.0000	3.0000	-5.3493	-1.1363	3.0768	1.0000
2.0000	4.0000	-4.0023	0.2108	4.4238	1.0000
3.0000	4.0000	-2.8660	1.3471	5.5601	1.0000

15.2480	1.1236
19.7000	1.1236
20.8363	1.1236
19.4892	1.1236

17

'Source'	'SS'	'df'	'MS'	'F'	'Prob>F'
'Columns'	[247.3407]	[3]	[82.4469]	[60.3298]	[3.0110e-32]
'Error'	[552.1078]	[404]	[1.3666]	[]	[]
'Total'	[799.4485]	[407]	[]	[]	[]

1.0000	2.0000	-1.7771	-1.3431	-0.9091	0.0000
1.0000	3.0000	-0.2183	0.2157	0.6497	1.0000
1.0000	4.0000	-1.9634	-1.5294	-1.0954	0.0000
2.0000	3.0000	1.1248	1.5588	1.9928	0.0000
2.0000	4.0000	-0.6203	-0.1863	0.2477	1.0000
3.0000	4.0000	-2.1791	-1.7451	-1.3111	0.0000

2.8725	0.1158
4.2157	0.1158
2.6569	0.1158
4.4020	0.1158

18

'Source'	'SS'	'df'	'MS'	'F'	'Prob>F'
'Columns'	[4.1862e+03]	[3]	[1.3954e+03]	[4.1881]	[0.0062]
'Error'	[1.3461e+05]	[404]	[333.1832]	[]	[]
'Total'	[1.3879e+05]	[407]	[]	[]	[]

1.0000	2.0000	-12.8061	-6.0294	0.7473	0.1128
1.0000	3.0000	-15.6198	-8.8431	-2.0664	0.0036
1.0000	4.0000	-12.2728	-5.4961	1.2806	0.1928
2.0000	3.0000	-9.5904	-2.8137	3.9630	1.0000
2.0000	4.0000	-6.2434	0.5333	7.3100	1.0000
3.0000	4.0000	-3.4296	3.3471	10.1237	1.0000

50.1206	1.8073
56.1500	1.8073
58.9637	1.8073
55.6167	1.8073

19

'Source'	'SS'	'df'	'MS'	'F'	'Prob>F'
'Columns'	[213.7917]	[3]	[71.2639]	[57.1076]	[8.5566e-31]
'Error'	[504.1471]	[404]	[1.2479]	[]	[]
'Total'	[717.9387]	[407]	[]	[]	[]

1.0000	2.0000	-1.9049	-1.4902	-1.0755	0.0000
1.0000	3.0000	-0.4049	0.0098	0.4245	1.0000
1.0000	4.0000	-1.8069	-1.3922	-0.9774	0.0000
2.0000	3.0000	1.0853	1.5000	1.9147	0.0000
2.0000	4.0000	-0.3167	0.0980	0.5128	1.0000
3.0000	4.0000	-1.8167	-1.4020	-0.9872	0.0000

1.5196	0.1106
3.0098	0.1106
1.5098	0.1106

2.9118 0.1106

20

'Source'	'SS'	'df'	'MS'	'F'	'Prob>F'
'Columns'	[93.5368]	[3]	[31.1789]	[17.8099]	[7.0970e-11]
'Error'	[707.2647]	[404]	[1.7507]	[]	[]
'Total'	[800.8015]	[407]	[]	[]	[]

1.0000	2.0000	-1.0795	-0.5882	-0.0970	0.0097
1.0000	3.0000	0.0088	0.5000	0.9912	0.0435
1.0000	4.0000	-1.1775	-0.6863	-0.1951	0.0014
2.0000	3.0000	0.5970	1.0882	1.5795	0.0000
2.0000	4.0000	-0.5893	-0.0980	0.3932	1.0000
3.0000	4.0000	-1.6775	-1.1863	-0.6951	0.0000

5.0784	0.1310
5.6667	0.1310
4.5784	0.1310
5.7647	0.1310

21

'Source'	'SS'	'df'	'MS'	'F'	'Prob>F'
'Columns'	[196.2034]	[3]	[65.4011]	[69.0171]	[4.7243e-36]
'Error'	[382.8333]	[404]	[0.9476]	[]	[]
'Total'	[579.0368]	[407]	[]	[]	[]

1.0000	2.0000	-1.5477	-1.1863	-0.8249	0.0000
1.0000	3.0000	-0.0379	0.3235	0.6849	0.1085
1.0000	4.0000	-1.5869	-1.2255	-0.8641	0.0000
2.0000	3.0000	1.1484	1.5098	1.8712	0.0000
2.0000	4.0000	-0.4006	-0.0392	0.3222	1.0000
3.0000	4.0000	-1.9104	-1.5490	-1.1876	0.0000

3.0882	0.0964
4.2745	0.0964
2.7647	0.0964
4.3137	0.0964

22

'Source'	'SS'	'df'	'MS'	'F'	'Prob>F'
'Columns'	[32.8701]	[3]	[10.9567]	[1.0507]	[0.3700]
'Error'	[4.2131e+03]	[404]	[10.4284]	[]	[]
'Total'	[4.2459e+03]	[407]	[]	[]	[]

1.0000	2.0000	-0.4342	0.7647	1.9636	0.5495
1.0000	3.0000	-0.8264	0.3725	1.5715	1.0000
1.0000	4.0000	-0.6205	0.5784	1.7773	1.0000
2.0000	3.0000	-1.5911	-0.3922	0.8067	1.0000
2.0000	4.0000	-1.3852	-0.1863	1.0126	1.0000
3.0000	4.0000	-0.9930	0.2059	1.4048	1.0000

4.6078	0.3197
3.8431	0.3197
4.2353	0.3197
4.0294	0.3197

23

'Source'	'SS'	'df'	'MS'	'F'	'Prob>F'
'Columns'	[2.6801e+03]	[3]	[893.3542]	[6.9194]	[1.4940e-04]
'Error'	[5.2160e+04]	[404]	[129.1081]	[]	[]
'Total'	[5.4840e+04]	[407]	[]	[]	[]

1.0000	2.0000	-10.2861	-6.0676	-1.8492	0.0010
1.0000	3.0000	-10.5734	-6.3549	-2.1365	0.0005
1.0000	4.0000	-9.1871	-4.9686	-0.7502	0.0115
2.0000	3.0000	-4.5057	-0.2873	3.9312	1.0000
2.0000	4.0000	-3.1194	1.0990	5.3175	1.0000
3.0000	4.0000	-2.8322	1.3863	5.6047	1.0000

21.9598	1.1251
28.0275	1.1251
28.3147	1.1251
26.9284	1.1251

24

'Source'	'SS'	'df'	'MS'	'F'	'Prob>F'
'Columns'	[4.9734e+06]	[3]	[1.6578e+06]	[2.6866]	[0.0462]
'Error'	[2.4930e+08]	[404]	[6.1707e+05]	[]	[]
'Total'	[2.5427e+08]	[407]	[]	[]	[]

1.0000	2.0000	-31.8827	259.7549	551.3925	0.1121
1.0000	3.0000	-45.8435	245.7941	537.4317	0.1560
1.0000	4.0000	-33.1964	258.4412	550.0788	0.1157
2.0000	3.0000	-305.5984	-13.9608	277.6768	1.0000
2.0000	4.0000	-292.9513	-1.3137	290.3239	1.0000
3.0000	4.0000	-278.9905	12.6471	304.2847	1.0000

475.6373	77.7799
215.8824	77.7799
229.8431	77.7799
217.1961	77.7799

25

'Source'	'SS'	'df'	'MS'	'F'	'Prob>F'
'Columns'	[81.6936]	[3]	[27.2312]	[8.1656]	[2.7340e-05]
'Error'	[1.3473e+03]	[404]	[3.3349]	[]	[]
'Total'	[1.4290e+03]	[407]	[]	[]	[]

1.0000	2.0000	0.2142	0.8922	1.5701	0.0032
1.0000	3.0000	-0.3152	0.3627	1.0407	0.9408
1.0000	4.0000	0.4691	1.1471	1.8250	0.0001
2.0000	3.0000	-1.2074	-0.5294	0.1486	0.2343
2.0000	4.0000	-0.4231	0.2549	0.9329	1.0000
3.0000	4.0000	0.1063	0.7843	1.4623	0.0138

2.8431	0.1808
1.9510	0.1808
2.4804	0.1808
1.6961	0.1808

26

'Source'	'SS'	'df'	'MS'	'F'	'Prob>F'
'Columns'	[1.9458e+04]	[3]	[6.4859e+03]	[10.5219]	[1.1203e-06]

'Error'	[2.4903e+05]	[404]	[616.4171]	[]	[]
'Total'	[2.6849e+05]	[407]	[]	[]	[]

1.0000	2.0000	-27.0852	-17.8676	-8.6501	0.0000
1.0000	3.0000	-17.0440	-7.8265	1.3910	0.1495
1.0000	4.0000	-24.1401	-14.9225	-5.7050	0.0001
2.0000	3.0000	0.8237	10.0412	19.2587	0.0245
2.0000	4.0000	-6.2724	2.9451	12.1626	1.0000
3.0000	4.0000	-16.3136	-7.0961	2.1214	0.2513

46.7059	2.4583
64.5735	2.4583
54.5324	2.4583
61.6284	2.4583

27

'Source'	'SS'	'df'	'MS'	'F'	'Prob>F'
'Columns'	[0]	[3]	[0]	[NaN]	[NaN]
'Error'	[0]	[404]	[0]	[]	[]
'Total'	[0]	[407]	[]	[]	[]

1	2	0	0	0	NaN
1	3	0	0	0	NaN
1	4	0	0	0	NaN
2	3	0	0	0	NaN
2	4	0	0	0	NaN
3	4	0	0	0	NaN

1	0
1	0
1	0
1	0

28

'Source'	'SS'	'df'	'MS'	'F'	'Prob>F'
'Columns'	[1.0745e+03]	[3]	[358.1789]	[26.3636]	[1.3601e-15]
'Error'	[5.4888e+03]	[404]	[13.5861]	[]	[]
'Total'	[6.5633e+03]	[407]	[]	[]	[]

1.0000	2.0000	2.7394	4.1078	5.4763	0.0000
1.0000	3.0000	1.1610	2.5294	3.8978	0.0000
1.0000	4.0000	2.4551	3.8235	5.1920	0.0000
2.0000	3.0000	-2.9469	-1.5784	-0.2100	0.0143
2.0000	4.0000	-1.6527	-0.2843	1.0841	1.0000
3.0000	4.0000	-0.0743	1.2941	2.6626	0.0753

8.1961	0.3650
4.0882	0.3650
5.6667	0.3650
4.3725	0.3650

29

'Source'	'SS'	'df'	'MS'	'F'	'Prob>F'
'Columns'	[309.2797]	[3]	[103.0932]	[1.2148]	[0.3040]
'Error'	[3.4286e+04]	[404]	[84.8674]	[]	[]
'Total'	[3.4596e+04]	[407]	[]	[]	[]

1.0000	2.0000	-1.0898	2.3304	5.7506	0.4295
1.0000	3.0000	-1.6849	1.7353	5.1555	1.0000
1.0000	4.0000	-1.6996	1.7206	5.1407	1.0000
2.0000	3.0000	-4.0153	-0.5951	2.8251	1.0000
2.0000	4.0000	-4.0300	-0.6098	2.8104	1.0000
3.0000	4.0000	-3.4349	-0.0147	3.4055	1.0000

7.3167	0.9122
4.9863	0.9122
5.5814	0.9122
5.5961	0.9122

30

'Source'	'SS'	'df'	'MS'	'F'	'Prob>F'
'Columns'	[1.7315e+04]	[3]	[5.7718e+03]	[13.3724]	[2.4479e-08]
'Error'	[1.7438e+05]	[404]	[431.6228]	[]	[]
'Total'	[1.9169e+05]	[407]	[]	[]	[]

1.0000	2.0000	7.5624	15.2755	22.9886	0.0000
1.0000	3.0000	4.0232	11.7363	19.4494	0.0004
1.0000	4.0000	8.7644	16.4775	24.1905	0.0000
2.0000	3.0000	-11.2523	-3.5392	4.1739	1.0000
2.0000	4.0000	-6.5111	1.2020	8.9150	1.0000
3.0000	4.0000	-2.9719	4.7412	12.4543	0.6236

27.4873	2.0571
12.2118	2.0571
15.7510	2.0571
11.0098	2.0571

31

'Source'	'SS'	'df'	'MS'	'F'	'Prob>F'
'Columns'	[9.7420e+03]	[3]	[3.2473e+03]	[9.1444]	[7.2287e-06]
'Error'	[1.4347e+05]	[404]	[355.1141]	[]	[]
'Total'	[1.5321e+05]	[407]	[]	[]	[]

1.0000	2.0000	2.6401	9.6363	16.6324	0.0018
1.0000	3.0000	-2.0236	4.9725	11.9687	0.3613
1.0000	4.0000	5.9617	12.9578	19.9540	0.0000
2.0000	3.0000	-11.6599	-4.6637	2.3324	0.4675
2.0000	4.0000	-3.6746	3.3216	10.3177	1.0000
3.0000	4.0000	0.9891	7.9853	14.9815	0.0158

25.3686	1.8659
15.7324	1.8659
20.3961	1.8659
12.4108	1.8659

32

'Source'	'SS'	'df'	'MS'	'F'	'Prob>F'
'Columns'	[7.1792e+03]	[3]	[2.3931e+03]	[5.4077]	[0.0012]
'Error'	[1.7878e+05]	[404]	[442.5277]	[]	[]
'Total'	[1.8596e+05]	[407]	[]	[]	[]

1.0000	2.0000	1.7695	9.5794	17.3893	0.0075
1.0000	3.0000	3.0391	10.8490	18.6589	0.0016
1.0000	4.0000	-0.8187	6.9912	14.8011	0.1086

2.0000	3.0000	-6.5403	1.2696	9.0795	1.0000
2.0000	4.0000	-10.3981	-2.5882	5.2217	1.0000
3.0000	4.0000	-11.6678	-3.8578	3.9521	1.0000

43.3725	2.0829
33.7931	2.0829
32.5235	2.0829
36.3814	2.0829

33

'Source'	'SS'	'df'	'MS'	'F'	'Prob>F'
'Columns'	[9.7456e+03]	[3]	[3.2485e+03]	[9.1483]	[7.1912e-06]
'Error'	[1.4346e+05]	[404]	[355.0980]	[]	[]
'Total'	[1.5321e+05]	[407]	[]	[]	[]

1.0000	2.0000	-16.6323	-9.6363	-2.6403	0.0018
1.0000	3.0000	-11.9686	-4.9725	2.0235	0.3613
1.0000	4.0000	-19.9568	-12.9608	-5.9648	0.0000
2.0000	3.0000	-2.3323	4.6637	11.6597	0.4675
2.0000	4.0000	-10.3205	-3.3245	3.6715	1.0000
3.0000	4.0000	-14.9842	-7.9882	-0.9922	0.0158

74.6324	1.8658
84.2686	1.8658
79.6049	1.8658
87.5931	1.8658

34

'Source'	'SS'	'df'	'MS'	'F'	'Prob>F'
'Columns'	[1.1409e+04]	[3]	[3.8030e+03]	[10.3114]	[1.4886e-06]
'Error'	[1.4900e+05]	[404]	[368.8187]	[]	[]
'Total'	[1.6041e+05]	[407]	[]	[]	[]

1.0000	2.0000	2.6485	9.7784	16.9083	0.0019
1.0000	3.0000	-2.2338	4.8961	12.0260	0.4164
1.0000	4.0000	7.0025	14.1324	21.2622	0.0000
2.0000	3.0000	-12.0122	-4.8824	2.2475	0.4211
2.0000	4.0000	-2.7760	4.3539	11.4838	0.6373
3.0000	4.0000	2.1064	9.2363	16.3662	0.0039

22.9520	1.9015
13.1735	1.9015
18.0559	1.9015
8.8196	1.9015

35

'Source'	'SS'	'df'	'MS'	'F'	'Prob>F'
'Columns'	[1.1405e+04]	[3]	[3.8016e+03]	[10.3077]	[1.4961e-06]
'Error'	[1.4900e+05]	[404]	[368.8126]	[]	[]
'Total'	[1.6041e+05]	[407]	[]	[]	[]

1.0000	2.0000	-16.9053	-9.7755	-2.6457	0.0019
1.0000	3.0000	-12.0230	-4.8931	2.2367	0.4174
1.0000	4.0000	-21.2592	-14.1294	-6.9996	0.0000
2.0000	3.0000	-2.2475	4.8824	12.0122	0.4211
2.0000	4.0000	-11.4837	-4.3539	2.7759	0.6373
3.0000	4.0000	-16.3661	-9.2363	-2.1064	0.0039

77.0510	1.9015
86.8265	1.9015
81.9441	1.9015
91.1804	1.9015

36

'Source'	'SS'	'df'	'MS'	'F'	'Prob>F'
'Columns'	[7.4240]	[3]	[2.4747]	[4.1731]	[0.0063]
'Error'	[239.5719]	[404]	[0.5930]	[]	[]
'Total'	[246.9959]	[407]	[]	[]	[]

1.0000	2.0000	-0.0800	0.2059	0.4918	0.3416
1.0000	3.0000	-0.3290	-0.0431	0.2428	1.0000
1.0000	4.0000	-0.0084	0.2775	0.5633	0.0626
2.0000	3.0000	-0.5349	-0.2490	0.0369	0.1286
2.0000	4.0000	-0.2143	0.0716	0.3575	1.0000
3.0000	4.0000	0.0347	0.3206	0.6065	0.0187

0.3882	0.0762
0.1824	0.0762
0.4314	0.0762
0.1108	0.0762

37

'Source'	'SS'	'df'	'MS'	'F'	'Prob>F'
'Columns'	[3.6548e+03]	[3]	[1.2183e+03]	[11.8343]	[1.9144e-07]
'Error'	[4.1590e+04]	[404]	[102.9448]	[]	[]
'Total'	[4.5245e+04]	[407]	[]	[]	[]

1.0000	2.0000	1.6047	5.3716	9.1384	0.0011
1.0000	3.0000	-2.5914	1.1755	4.9423	1.0000
1.0000	4.0000	3.5645	7.3314	11.0982	0.0000
2.0000	3.0000	-7.9629	-4.1961	-0.4292	0.0200
2.0000	4.0000	-1.8070	1.9598	5.7267	1.0000
3.0000	4.0000	2.3890	6.1559	9.9227	0.0001

12.2912	1.0046
6.9196	1.0046
11.1157	1.0046
4.9598	1.0046

38

'Source'	'SS'	'df'	'MS'	'F'	'Prob>F'
'Columns'	[3.4857e+03]	[3]	[1.1619e+03]	[11.5703]	[2.7293e-07]
'Error'	[4.0570e+04]	[404]	[100.4213]	[]	[]
'Total'	[4.4056e+04]	[407]	[]	[]	[]

1.0000	2.0000	1.6286	5.3490	9.0694	0.0010
1.0000	3.0000	-2.6557	1.0647	4.7851	1.0000
1.0000	4.0000	3.3355	7.0559	10.7763	0.0000
2.0000	3.0000	-8.0047	-4.2843	-0.5639	0.0145
2.0000	4.0000	-2.0135	1.7069	5.4273	1.0000
3.0000	4.0000	2.2708	5.9912	9.7116	0.0001

12.0020	0.9922
6.6529	0.9922

10.9373	0.9922
4.9461	0.9922

39

'Source'	'SS'	'df'	'MS'	'F'	'Prob>F'
'Columns'	[2.4263e+03]	[3]	[808.7695]	[4.8011]	[0.0027]
'Error'	[6.8056e+04]	[404]	[168.4548]	[]	[]
'Total'	[7.0482e+04]	[407]	[]	[]	[]

1.0000	2.0000	-0.4137	4.4049	9.2235	0.0948
1.0000	3.0000	-1.0970	3.7216	8.5401	0.2474
1.0000	4.0000	1.9805	6.7990	11.6176	0.0013
2.0000	3.0000	-5.5019	-0.6833	4.1352	1.0000
2.0000	4.0000	-2.4244	2.3941	7.2127	1.0000
3.0000	4.0000	-1.7411	3.0775	7.8960	0.5470

10.6598	1.2851
6.2549	1.2851
6.9382	1.2851
3.8608	1.2851

40

'Source'	'SS'	'df'	'MS'	'F'	'Prob>F'
'Columns'	[2.7165e+04]	[3]	[9.0551e+03]	[11.8390]	[1.9024e-07]
'Error'	[3.0900e+05]	[404]	[764.8483]	[]	[]
'Total'	[3.3616e+05]	[407]	[]	[]	[]

1.0000	2.0000	-25.3851	-15.1176	-4.8502	0.0007
1.0000	3.0000	-16.2136	-5.9461	4.3214	0.7528
1.0000	4.0000	-31.4459	-21.1784	-10.9110	0.0000
2.0000	3.0000	-1.0959	9.1716	19.4390	0.1100
2.0000	4.0000	-16.3283	-6.0608	4.2067	0.7101
3.0000	4.0000	-25.4998	-15.2324	-4.9649	0.0006

65.0588	2.7383
80.1765	2.7383
71.0049	2.7383
86.2373	2.7383

41

'Source'	'SS'	'df'	'MS'	'F'	'Prob>F'
'Columns'	[159.6320]	[3]	[53.2107]	[1.5595]	[0.1987]
'Error'	[1.3785e+04]	[404]	[34.1202]	[]	[]
'Total'	[1.3944e+04]	[407]	[]	[]	[]

1.0000	2.0000	-1.2265	0.9422	3.1108	1.0000
1.0000	3.0000	-0.6725	1.4961	3.6647	0.4087
1.0000	4.0000	-2.1049	0.0637	2.2323	1.0000
2.0000	3.0000	-1.6147	0.5539	2.7225	1.0000
2.0000	4.0000	-3.0470	-0.8784	1.2902	1.0000
3.0000	4.0000	-3.6010	-1.4324	0.7363	0.4840

47.3382	0.5784
46.3961	0.5784
45.8422	0.5784
47.2745	0.5784

42

'Source'	'SS'	'df'	'MS'	'F'	'Prob>F'
'Columns'	[148.5865]	[3]	[49.5288]	[1.2858]	[0.2788]
'Error'	[1.5562e+04]	[404]	[38.5189]	[]	[]
'Total'	[1.5710e+04]	[407]	[]	[]	[]

1.0000	2.0000	-3.6130	-1.3088	0.9953	0.7971
1.0000	3.0000	-3.9071	-1.6029	0.7012	0.3951
1.0000	4.0000	-3.2512	-0.9471	1.3571	1.0000
2.0000	3.0000	-2.5983	-0.2941	2.0100	1.0000
2.0000	4.0000	-1.9424	0.3618	2.6659	1.0000
3.0000	4.0000	-1.6483	0.6559	2.9600	1.0000

48.7794	0.6145
50.0882	0.6145
50.3824	0.6145
49.7265	0.6145

43

'Source'	'SS'	'df'	'MS'	'F'	'Prob>F'
'Columns'	[46.7434]	[3]	[15.5811]	[1.3817]	[0.2479]
'Error'	[4.5559e+03]	[404]	[11.2771]	[]	[]
'Total'	[4.6027e+03]	[407]	[]	[]	[]

1.0000	2.0000	-0.8781	0.3686	1.6154	1.0000
1.0000	3.0000	-1.1438	0.1029	1.3497	1.0000
1.0000	4.0000	-0.3722	0.8745	2.1212	0.3819
2.0000	3.0000	-1.5124	-0.2657	0.9811	1.0000
2.0000	4.0000	-0.7409	0.5059	1.7526	1.0000
3.0000	4.0000	-0.4752	0.7716	2.0183	0.6097

3.8814	0.3325
3.5127	0.3325
3.7784	0.3325
3.0069	0.3325

44

'Source'	'SS'	'df'	'MS'	'F'	'Prob>F'
'Columns'	[2.4905e+03]	[3]	[830.1573]	[8.3336]	[2.1755e-05]
'Error'	[4.0245e+04]	[404]	[99.6163]	[]	[]
'Total'	[4.2735e+04]	[407]	[]	[]	[]

1.0000	2.0000	-9.3731	-5.6676	-1.9622	0.0004
1.0000	3.0000	-9.7760	-6.0706	-2.3651	0.0001
1.0000	4.0000	-6.2662	-2.5608	1.1447	0.4059
2.0000	3.0000	-4.1084	-0.4029	3.3025	1.0000
2.0000	4.0000	-0.5986	3.1069	6.8123	0.1606
3.0000	4.0000	-0.1956	3.5098	7.2153	0.0745

7.2873	0.9882
12.9549	0.9882
13.3578	0.9882
9.8480	0.9882

45

'Source'	'SS'	'df'	'MS'	'F'	'Prob>F'

'Columns'	[2.5202e+03]	[3]	[840.0622]	[8.3797]	[2.0432e-05]
'Error'	[4.0501e+04]	[404]	[100.2498]	[]	[]
'Total'	[4.3021e+04]	[407]	[]	[]	[]

1.0000	2.0000	1.9965	5.7137	9.4309	0.0003
1.0000	3.0000	2.3720	6.0892	9.8064	0.0001
1.0000	4.0000	-1.1584	2.5588	6.2760	0.4124
2.0000	3.0000	-3.3417	0.3755	4.0927	1.0000
2.0000	4.0000	-6.8721	-3.1549	0.5623	0.1498
3.0000	4.0000	-7.2476	-3.5304	0.1868	0.0731

92.7127	0.9914
86.9990	0.9914
86.6235	0.9914
90.1539	0.9914

46

'Source'	'SS'	'df'	'MS'	'F'	'Prob>F'
'Columns'	[2.2899e+03]	[3]	[763.3158]	[2.7818]	[0.0407]
'Error'	[1.1086e+05]	[404]	[274.3974]	[]	[]
'Total'	[1.1315e+05]	[407]	[]	[]	[]

1.0000	2.0000	-0.7656	5.3843	11.5342	0.1246
1.0000	3.0000	-0.3724	5.7775	11.9273	0.0789
1.0000	4.0000	-0.9646	5.1853	11.3352	0.1556
2.0000	3.0000	-5.7567	0.3931	6.5430	1.0000
2.0000	4.0000	-6.3489	-0.1990	5.9509	1.0000
3.0000	4.0000	-6.7420	-0.5922	5.5577	1.0000

22.2010	1.6402
16.8167	1.6402
16.4235	1.6402
17.0157	1.6402

47

'Source'	'SS'	'df'	'MS'	'F'	'Prob>F'
'Columns'	[2.1603e+03]	[3]	[720.0922]	[0.7753]	[0.5083]
'Error'	[3.7522e+05]	[404]	[928.7676]	[]	[]
'Total'	[3.7738e+05]	[407]	[]	[]	[]

1.0000	2.0000	-15.5898	-4.2755	7.0389	1.0000
1.0000	3.0000	-14.2957	-2.9814	8.3330	1.0000
1.0000	4.0000	-9.8065	1.5078	12.8222	1.0000
2.0000	3.0000	-10.0202	1.2941	12.6085	1.0000
2.0000	4.0000	-5.5310	5.7833	17.0977	1.0000
3.0000	4.0000	-6.8251	4.4892	15.8036	1.0000

59.5716	3.0175
63.8471	3.0175
62.5529	3.0175
58.0637	3.0175

48

'Source'	'SS'	'df'	'MS'	'F'	'Prob>F'
'Columns'	[971.1589]	[3]	[323.7196]	[1.9957]	[0.1140]
'Error'	[6.5532e+04]	[404]	[162.2086]	[]	[]
'Total'	[6.6503e+04]	[407]	[]	[]	[]

1.0000	2.0000	-8.7872	-4.0588	0.6696	0.1403
1.0000	3.0000	-6.6549	-1.9265	2.8019	1.0000
1.0000	4.0000	-8.0284	-3.3000	1.4284	0.3899
2.0000	3.0000	-2.5960	2.1324	6.8607	1.0000
2.0000	4.0000	-3.9696	0.7588	5.4872	1.0000
3.0000	4.0000	-6.1019	-1.3735	3.3549	1.0000

13.0373	1.2611
17.0961	1.2611
14.9637	1.2611
16.3373	1.2611

49

'Source'	'SS'	'df'	'MS'	'F'	'Prob>F'
'Columns'	[339.2491]	[3]	[113.0830]	[5.7791]	[7.0787e-04]
'Error'	[7.9053e+03]	[404]	[19.5675]	[]	[]
'Total'	[8.2445e+03]	[407]	[]	[]	[]

1.0000	2.0000	0.4175	2.0598	3.7021	0.0058
1.0000	3.0000	-0.6129	1.0294	2.6717	0.5838
1.0000	4.0000	0.6548	2.2971	3.9393	0.0014
2.0000	3.0000	-2.6727	-1.0304	0.6119	0.5819
2.0000	4.0000	-1.4050	0.2373	1.8795	1.0000
3.0000	4.0000	-0.3746	1.2676	2.9099	0.2481

3.8186	0.4380
1.7588	0.4380
2.7892	0.4380
1.5216	0.4380

50

'Source'	'SS'	'df'	'MS'	'F'	'Prob>F'
'Columns'	[597.2689]	[3]	[199.0896]	[2.8348]	[0.0380]
'Error'	[2.8373e+04]	[404]	[70.2309]	[]	[]
'Total'	[2.8971e+04]	[407]	[]	[]	[]

1.0000	2.0000	-4.7750	-1.6637	1.4476	0.9422
1.0000	3.0000	-1.6407	1.4706	4.5819	1.0000
1.0000	4.0000	-4.2936	-1.1824	1.9289	1.0000
2.0000	3.0000	0.0230	3.1343	6.2456	0.0472
2.0000	4.0000	-2.6299	0.4814	3.5927	1.0000
3.0000	4.0000	-5.7642	-2.6529	0.4583	0.1458

11.5235	0.8298
13.1873	0.8298
10.0529	0.8298
12.7059	0.8298

51

'Source'	'SS'	'df'	'MS'	'F'	'Prob>F'
'Columns'	[337.1848]	[3]	[112.3949]	[3.3569]	[0.0189]
'Error'	[1.3527e+04]	[404]	[33.4815]	[]	[]
'Total'	[1.3864e+04]	[407]	[]	[]	[]

1.0000	2.0000	-2.1335	0.0147	2.1629	1.0000
1.0000	3.0000	-2.7453	-0.5971	1.5512	1.0000

1.0000	4.0000	-0.3217	1.8265	3.9747	0.1483
2.0000	3.0000	-2.7600	-0.6118	1.5365	1.0000
2.0000	4.0000	-0.3365	1.8118	3.9600	0.1554
3.0000	4.0000	0.2753	2.4235	4.5717	0.0177

3.2118	0.5729
3.1971	0.5729
3.8088	0.5729
1.3853	0.5729

52

'Source'	'SS'	'df'	'MS'	'F'	'Prob>F'
'Columns'	[335.5158]	[3]	[111.8386]	[1.4697]	[0.2222]
'Error'	[3.0742e+04]	[404]	[76.0940]	[]	[]
'Total'	[3.1077e+04]	[407]	[]	[]	[]

1.0000	2.0000	-4.6788	-1.4402	1.7984	1.0000
1.0000	3.0000	-5.4248	-2.1863	1.0523	0.4454
1.0000	4.0000	-5.4925	-2.2539	0.9846	0.3944
2.0000	3.0000	-3.9846	-0.7461	2.4925	1.0000
2.0000	4.0000	-4.0523	-0.8137	2.4248	1.0000
3.0000	4.0000	-3.3062	-0.0676	3.1709	1.0000

11.1784	0.8637
12.6186	0.8637
13.3647	0.8637
13.4324	0.8637

53

'Source'	'SS'	'df'	'MS'	'F'	'Prob>F'
'Columns'	[123.9025]	[3]	[41.3008]	[0.4641]	[0.7075]
'Error'	[3.5952e+04]	[404]	[88.9901]	[]	[]
'Total'	[3.6076e+04]	[407]	[]	[]	[]

1.0000	2.0000	-4.2179	-0.7157	2.7866	1.0000
1.0000	3.0000	-4.8640	-1.3618	2.1405	1.0000
1.0000	4.0000	-4.8130	-1.3108	2.1915	1.0000
2.0000	3.0000	-4.1483	-0.6461	2.8562	1.0000
2.0000	4.0000	-4.0973	-0.5951	2.9072	1.0000
3.0000	4.0000	-3.4513	0.0510	3.5532	1.0000

10.7627	0.9341
11.4784	0.9341
12.1245	0.9341
12.0735	0.9341

54

'Source'	'SS'	'df'	'MS'	'F'	'Prob>F'
'Columns'	[362.2468]	[3]	[120.7489]	[4.3395]	[0.0050]
'Error'	[1.1241e+04]	[404]	[27.8253]	[]	[]
'Total'	[1.1604e+04]	[407]	[]	[]	[]

1.0000	2.0000	-0.3496	1.6088	3.5672	0.1799
1.0000	3.0000	-1.8152	0.1431	2.1015	1.0000
1.0000	4.0000	0.2446	2.2029	4.1613	0.0182
2.0000	3.0000	-3.4241	-1.4657	0.4927	0.2874
2.0000	4.0000	-1.3643	0.5941	2.5525	1.0000

3.0000	4.0000	0.1014	2.0598	4.0182	0.0333

4.0500	0.5223
2.4412	0.5223
3.9069	0.5223
1.8471	0.5223

55

'Source'	'SS'	'df'	'MS'	'F'	'Prob>F'
'Columns'	[1.0274e+03]	[3]	[342.4583]	[4.4726]	[0.0042]
'Error'	[3.0934e+04]	[404]	[76.5685]	[]	[]
'Total'	[3.1961e+04]	[407]	[]	[]	[]

1.0000	2.0000	-5.9800	-2.7314	0.5173	0.1581
1.0000	3.0000	-4.7830	-1.5343	1.7143	1.0000
1.0000	4.0000	-7.5741	-4.3255	-1.0769	0.0028
2.0000	3.0000	-2.0516	1.1971	4.4457	1.0000
2.0000	4.0000	-4.8428	-1.5941	1.6545	1.0000
3.0000	4.0000	-6.0398	-2.7912	0.4575	0.1395

10.9049	0.8664
13.6363	0.8664
12.4392	0.8664
15.2304	0.8664

56

'Source'	'SS'	'df'	'MS'	'F'	'Prob>F'
'Columns'	[378.4441]	[3]	[126.1480]	[5.1131]	[0.0018]
'Error'	[9.9673e+03]	[404]	[24.6715]	[]	[]
'Total'	[1.0346e+04]	[407]	[]	[]	[]

1.0000	2.0000	-0.2293	1.6147	3.4588	0.1245
1.0000	3.0000	-0.5735	1.2706	3.1146	0.4108
1.0000	4.0000	0.8550	2.6990	4.5431	0.0007
2.0000	3.0000	-2.1882	-0.3441	1.4999	1.0000
2.0000	4.0000	-0.7597	1.0843	2.9284	0.7187
3.0000	4.0000	-0.4156	1.4284	3.2725	0.2439

4.1755	0.4918
2.5608	0.4918
2.9049	0.4918
1.4765	0.4918

57

'Source'	'SS'	'df'	'MS'	'F'	'Prob>F'
'Columns'	[280.4071]	[3]	[93.4690]	[1.3521]	[0.2571]
'Error'	[2.7928e+04]	[404]	[69.1298]	[]	[]
'Total'	[2.8209e+04]	[407]	[]	[]	[]

1.0000	2.0000	-2.1201	0.9667	4.0535	1.0000
1.0000	3.0000	-1.6290	1.4578	4.5446	1.0000
1.0000	4.0000	-3.7613	-0.6745	2.4123	1.0000
2.0000	3.0000	-2.5956	0.4912	3.5780	1.0000
2.0000	4.0000	-4.7280	-1.6412	1.4456	0.9565
3.0000	4.0000	-5.2192	-2.1324	0.9544	0.4066

11.7637	0.8233

10.7971	0.8233
10.3059	0.8233
12.4382	0.8233

58

'Source'	'SS'	'df'	'MS'	'F'	'Prob>F'
'Columns'	[455.2134]	[3]	[151.7378]	[5.7296]	[7.5739e-04]
'Error'	[1.0699e+04]	[404]	[26.4833]	[]	[]
'Total'	[1.1154e+04]	[407]	[]	[]	[]

1.0000	2.0000	-0.2537	1.6569	3.5674	0.1320
1.0000	3.0000	-1.1066	0.8039	2.7145	1.0000
1.0000	4.0000	0.9394	2.8500	4.7606	0.0005
2.0000	3.0000	-2.7635	-0.8529	1.0576	1.0000
2.0000	4.0000	-0.7174	1.1931	3.1037	0.5913
3.0000	4.0000	0.1355	2.0461	3.9566	0.0285

4.2961	0.5095
2.6392	0.5095
3.4922	0.5095
1.4461	0.5095

59

'Source'	'SS'	'df'	'MS'	'F'	'Prob>F'
'Columns'	[376.1065]	[3]	[125.3688]	[1.9041]	[0.1283]
'Error'	[2.6600e+04]	[404]	[65.8418]	[]	[]
'Total'	[2.6976e+04]	[407]	[]	[]	[]

1.0000	2.0000	-0.3096	2.7029	5.7154	0.1070
1.0000	3.0000	-1.5919	1.4206	4.4331	1.0000
1.0000	4.0000	-1.8419	1.1706	4.1831	1.0000
2.0000	3.0000	-4.2949	-1.2824	1.7301	1.0000
2.0000	4.0000	-4.5449	-1.5324	1.4801	1.0000
3.0000	4.0000	-3.2625	-0.2500	2.7625	1.0000

11.2735	0.8034
8.5706	0.8034
9.8529	0.8034
10.1029	0.8034
