# Supplementary material for: Computational elucidation of the effects induced by music making
Source: PLoS One. 2019 Mar 7;14(3):e0213247. doi: 10.1371/journal.pone.0213247 (PMC6405055; doi:10.1371/journal.pone.0213247)
Supplement: S3 File — (PDF) [file pone.0213247.s012.pdf]

Demographic study (Females vs. Males, Professionals vs. Laymen, Young vs. Old) - MATLAB *t*-test results

Attribute	*Parameter*	Column# in results
**Time**	% playing time	2
	% idle time	4
	% start time	46
	% concurrent	3
	total (minutes)	1
**Notes/ Keys**	# of presses	10
	% used	11
	presses per key	29
	play per key (sec)	12
	% black presses	34
	% white presses	35
**Intensity**†	average	9
	lowest (minimum)	7
	highest (maximum)	8
	most used	5
**Octave**	average	21
	lowest (minimum)	19
	highest (maximum)	20
	most used	17
**Cluster of notes**	# of instances	24
	max pressed‡	28
	most pressed§	25
	% most played‖	26
**Transit-ions**	% diminuendo	41
	% crescendo	42
	% same intensity	43
	% accelerando	44
	% ritardando	45
	% white to black	37
	% black to white	38
	% black to black	39
	% white to white	40
**Pitch classes**	% playing time: C,C#,D,D#,E,F,F#, G,G#,A,A#,B	48-59

† 1-*pppp* ; 2-*ppp* ; 3-*pp* ; 4-*p* ; 5-*mp* ; 6-*mf* ; 7-*f* ; 8-*ff* ; 9-*fff* ; 10-*ffff*
‡ configuration of maximum number of keys pressed ; § most pressed configuration ;
‖ relative playing time of the most pressed configuration;

https://www.mathworks.com/help/stats/ttest2.html

**Variable**	**Definition**
Parameters matrix representation	
F	Females mean
M	Males mean
L	Laymen mean
P	Professional mean
Y	Young mean
O	Old mean
d	Cohen's D size effect
Test the alternative hypothesis that the population means are not equal.	
h	Hypothesis test result, returned as 1 or 0
p	*p*-value of the test
ci	Confidence interval for the *difference* in population means of x and y
stats.tstat	Value of the test statistic
stats.df	Degrees of freedom of the test
stats.sd	Unpooled estimates of the population SD
Test the alternative hypothesis that the population mean of x is greater than the population mean of y.	
hr	Hypothesis test result, returned as 1 or 0
pr	*p*-value of the test
cir	Confidence interval for the *difference* in population means of x and y
statsr.tstat	Value of the test statistic
statsr.df	Degrees of freedom of the test
statsr.sd	Unpooled estimates of the population SD
Test the alternative hypothesis that the population mean of x is less than the population mean of y.	
hl	Hypothesis test result, returned as 1 or 0
pl	*p*-value of the test
cil	Confidence interval for the *difference* in population means of x and y
statsl.tstat	Value of the test statistic
statsl.df	Degrees of freedom of the test
statsl.sd	Unpooled estimates of the population SD

Females vs. Males

```
>> F

F =

  Columns 1 through 10

    0.7590   69.7429  257.0637   30.2571    6.2311   40.3637    2.4292    8.1321    5.9245  117.7217

  Columns 11 through 20

   26.8698    0.4167  336.3042   58.7547    3.4953   18.8439    3.5377   56.6241    2.2972    5.1745

  Columns 21 through 30

    3.6368    4.0613   26.2401  218.5047    2.4387   56.6632    1.0000    5.5613    4.6783   15.5075

  Columns 31 through 40

   18.9892   34.6943   81.0127   16.5165   83.4844    0.2340    9.3382    9.1736    7.1783   74.3151

  Columns 41 through 50

   46.5090   49.7179    3.7759   11.0726   88.9151   17.9722   63.7099   15.3123    2.3882   11.5458

  Columns 51 through 60

    2.8679   12.8811   11.4660    3.4915   13.7288    2.8217   10.3887    3.1297    9.9731       NaN

>> M

M =

  Columns 1 through 10

    0.9542   67.3893  259.8360   32.6107    6.4907   39.2084    2.2290    8.4439    6.1028  206.3178

  Columns 11 through 20

   30.8762    0.3740  336.6682   58.6028    3.5140   18.5252    3.5421   52.9986    2.1636    5.4252

  Columns 21 through 30

    3.5981    4.2430   26.6009  381.1869    2.2710   55.1678    1.0000    5.9439    7.1720   18.8322

  Columns 31 through 40

   18.4804   39.1780   81.5210   15.5322   84.4682    0.3280    8.7780    8.5799    6.7542   75.8916
```

Columns 41 through 50

46.9294   49.5977   3.4724   11.0640   88.9150   18.1808   58.4701   15.1215   2.6136   11.9500

Columns 51 through 60

2.9065   13.0383   11.5014   2.8150   12.2463   2.8953   12.0486   2.8477   10.0103   NaN

>> h

h =

Columns 1 through 18

0   0   0   0   0   0   0   1   0   1   1   0   0   0   0   0   0   1

Columns 19 through 36

0   0   0   0   0   1   0   0   NaN   0   1   0   0   1   0   0   0   0

Columns 37 through 54

0   0   0   0   0   0   0   0   0   0   0   0   0   0   0   0   0   0

Columns 55 through 60

0   0   1   0   0   NaN

>> p

p =

Columns 1 through 10

0.1336   0.2573   0.8774   0.2573   0.1052   0.3274   0.1753   0.0106   0.1687   0.0335

Columns 11 through 20

0.0311   0.2307   0.9942   0.9265   0.8934   0.7730   0.9744   0.0408   0.2933   0.0628

Columns 21 through 30

0.7341   0.5605   0.7542   0.0423   0.4422   0.5471   NaN   0.3695   0.0051   0.1241

Columns 31 through 40

0.7861   0.0303   0.7863   0.6080   0.6082   0.2035   0.5853   0.5582   0.7371   0.5718

Columns 41 through 50

```
    0.4559    0.8415    0.4069    0.9931    0.9999    0.8977    0.0762    0.8774    0.6043    0.6191

  Columns 51 through 60

    0.9448    0.8665    0.9687    0.1931    0.0832    0.8813    0.0377    0.5820    0.9623       NaN

>> ci

ci =

  Columns 1 through 10

   -0.4506   -1.7245  -38.0858   -6.4318   -0.5737   -1.1606   -0.0897   -0.5508   -0.4324 -170.2451
    0.0601    6.4318   32.5412    1.7245    0.0547    3.4711    0.4902   -0.0729    0.0759   -6.9470

  Columns 11 through 20

   -7.6463   -0.0273  -97.9204   -3.0818   -0.2934   -1.8511   -0.2685    0.1528   -0.1160   -0.5149
   -0.3664    0.1128   97.1924    3.3856    0.2560    2.4884    0.2598    7.0982    0.3832    0.0135

  Columns 21 through 30

   -0.1849   -0.7947   -2.6244 -319.6356   -0.2610   -3.3826       NaN   -1.2198   -4.2309   -7.5654
    0.2623    0.4313    1.9027   -5.7288    0.5963    6.3735       NaN    0.4546   -0.7564    0.9160

  Columns 31 through 40

   -3.1748   -8.5398   -4.1918   -2.7848   -4.7528   -0.2394   -1.4563   -1.3975   -2.0574   -7.0525
    4.1923   -0.4276    3.1752    4.7533    2.7852    0.0512    2.5766    2.5849    2.9056    3.8995

  Columns 41 through 50

   -1.5280   -1.0609   -0.4152   -1.9404   -1.9553   -3.3965   -0.5543   -2.2381   -1.0794   -2.0014
    0.6870    1.3015    1.0222    1.9576    1.9556    2.9791   11.0339    2.6196    0.6288    1.1929

  Columns 51 through 60

   -1.1344   -1.9935   -1.8059   -0.3437   -0.1953   -1.0420   -3.2250   -0.7242   -1.5820       NaN
    1.0571    1.6792    1.7352    1.6968    3.1604    0.8948   -0.0948    1.2883    1.5076       NaN

>> stats.tstat

ans =

  Columns 1 through 10

   -1.5031    1.1344   -0.1543   -1.1344   -1.6236    0.9805    1.3576   -2.5658   -1.3788   -2.1352

  Columns 11 through 20
```

```
   -2.1636    1.2004   -0.0073    0.0923   -0.1341    0.2887   -0.0321    2.0520    1.0523   -1.8655

  Columns 21 through 30

    0.3399   -0.5825   -0.3134   -2.0396    0.7694    0.6026       NaN   -0.8983   -2.8260   -1.5411

  Columns 31 through 40

    0.2715   -2.1729   -0.2712    0.5133   -0.5131   -1.2749    0.5460    0.5861    0.3360   -0.5659

  Columns 41 through 50

   -0.7463    0.2001    0.8303    0.0087    0.0001   -0.1287    1.7776    0.1544   -0.5186   -0.4975

  Columns 51 through 60

   -0.0693   -0.1683   -0.0393    1.3036    1.7370   -0.1494   -2.0847    0.5510   -0.0473       NaN

>> stats.df

ans =

  Columns 1 through 10

  417.7926  423.9781  422.9375  423.9781  422.5963  422.5755  419.2995  423.9988  422.7089  304.2942

  Columns 11 through 20

  412.6516  403.8743  385.2267  423.1365  422.5309  396.7257  423.7724  423.9777  420.8204  419.7811

  Columns 21 through 30

  423.7515  423.6452  414.7243  304.7293  341.7154  423.9703       NaN  423.9130  269.9686  415.3424

  Columns 31 through 40

  420.8359  419.9029  420.8390  421.6248  421.6266  270.5086  419.8334  419.6895  408.1686  422.6593

  Columns 41 through 50

  423.5113  422.8458  389.3274  423.7112  423.6242  423.3816  422.5603  399.4475  423.7061  421.7840

  Columns 51 through 60

  423.4015  421.7565  421.8504  401.1543  407.3811  423.6396  421.6604  423.5592  412.7178       NaN

>> stats.sd

ans =
```

```
  1.0e+03 *

 Columns 1 through 10

   0.0014    0.0214    0.1891    0.0214    0.0017    0.0124    0.0016    0.0012    0.0014    0.2592
   0.0013    0.0214    0.1816    0.0214    0.0016    0.0119    0.0014    0.0013    0.0013    0.5483

 Columns 11 through 20

   0.0174    0.0004    0.5853    0.0165    0.0014    0.0097    0.0014    0.0182    0.0014    0.0014
   0.0207    0.0003    0.4256    0.0174    0.0015    0.0129    0.0014    0.0183    0.0013    0.0013

 Columns 21 through 30

   0.0012    0.0032    0.0109    0.4993    0.0027    0.0256         0    0.0043    0.0045    0.0205
   0.0012    0.0033    0.0128    1.0535    0.0016    0.0256         0    0.0044    0.0121    0.0239

 Columns 31 through 40

   0.0201    0.0201    0.0201    0.0204    0.0204    0.0004    0.0111    0.0109    0.0142    0.0294
   0.0186    0.0224    0.0186    0.0191    0.0191    0.0010    0.0101    0.0100    0.0117    0.0281

 Columns 41 through 50

   0.0059    0.0060    0.0043    0.0101    0.0101    0.0170    0.0312    0.0110    0.0045    0.0080
   0.0057    0.0064    0.0032    0.0104    0.0105    0.0165    0.0297    0.0143    0.0044    0.0087

 Columns 51 through 60

   0.0056    0.0099    0.0096    0.0059    0.0096    0.0050    0.0079    0.0053    0.0074       NaN
   0.0059    0.0093    0.0090    0.0047    0.0079    0.0052    0.0086    0.0052    0.0088       NaN

>>
```

```
>> hr

hr =

  Columns 1 through 18

     0     0     0     0     0     0     0     0     0     0     0     0     0     0     0     0     0     1

  Columns 19 through 36

     0     0     0     0     0     0     0     0   NaN     0     0     0     0     0     0     0     0     0

  Columns 37 through 54

     0     0     0     0     0     0     0     0     0     0     1     0     0     0     0     0     0     0

  Columns 55 through 60

     1     0     0     0     0   NaN

>> pr

pr =

  Columns 1 through 10

    0.9332    0.1286    0.5613    0.8714    0.9474    0.1637    0.0877    0.9947    0.9157    0.9832

  Columns 11 through 20

    0.9845    0.1154    0.5029    0.4632    0.5533    0.3865    0.5128    0.0204    0.1466    0.9686

  Columns 21 through 30

    0.3671    0.7197    0.6229    0.9789    0.2211    0.2736       NaN    0.8152    0.9975    0.9380

  Columns 31 through 40

    0.3931    0.9848    0.6068    0.3040    0.6959    0.8983    0.2927    0.2791    0.3685    0.7141

  Columns 41 through 50

    0.7720    0.4207    0.2034    0.4965    0.4999    0.5512    0.0381    0.4387    0.6978    0.6904

  Columns 51 through 60

    0.5276    0.5668    0.5156    0.0966    0.0416    0.5594    0.9812    0.2910    0.5188       NaN

>> cir
```

```
cir =

  Columns 1 through 10

   -0.4094   -1.0665  -32.3884   -5.7739   -0.5230   -0.7869   -0.0429   -0.5122   -0.3914 -157.0540
       Inf       Inf       Inf       Inf       Inf       Inf       Inf       Inf       Inf       Inf

  Columns 11 through 20

   -7.0590   -0.0160  -82.1754   -2.5601   -0.2491   -1.5010   -0.2259    0.7130   -0.0757   -0.4722
       Inf       Inf       Inf       Inf       Inf       Inf       Inf       Inf       Inf       Inf

  Columns 21 through 30

   -0.1489   -0.6958   -2.2592 -294.2785   -0.1918   -2.5956       NaN   -1.0847   -3.9501   -6.8812
       Inf       Inf       Inf       Inf       Inf       Inf       Inf       Inf       Inf       Inf

  Columns 31 through 40

   -2.5805   -7.8853   -3.5975   -2.1767   -4.1447   -0.2159   -1.1310   -1.0762   -1.6570   -6.1690
       Inf       Inf       Inf       Inf       Inf       Inf       Inf       Inf       Inf       Inf

  Columns 41 through 50

   -1.3493   -0.8704   -0.2992   -1.6259   -1.6398   -2.8821    0.3805   -1.8461   -0.9416   -1.7437
       Inf       Inf       Inf       Inf       Inf       Inf       Inf       Inf       Inf       Inf

  Columns 51 through 60

   -0.9576   -1.6973   -1.5202   -0.1791    0.0754   -0.8858   -2.9725   -0.5618   -1.3327       NaN
       Inf       Inf       Inf       Inf       Inf       Inf       Inf       Inf       Inf       Inf


>> statsr.tstat

ans =

  Columns 1 through 10

   -1.5031    1.1344   -0.1543   -1.1344   -1.6236    0.9805    1.3576   -2.5658   -1.3788   -2.1352

  Columns 11 through 20

   -2.1636    1.2004   -0.0073    0.0923   -0.1341    0.2887   -0.0321    2.0520    1.0523   -1.8655

  Columns 21 through 30

    0.3399   -0.5825   -0.3134   -2.0396    0.7694    0.6026       NaN   -0.8983   -2.8260   -1.5411

  Columns 31 through 40
```

```
    0.2715   -2.1729   -0.2712    0.5133   -0.5131   -1.2749    0.5460    0.5861    0.3360   -0.5659

  Columns 41 through 50

   -0.7463    0.2001    0.8303    0.0087    0.0001   -0.1287    1.7776    0.1544   -0.5186   -0.4975

  Columns 51 through 60

   -0.0693   -0.1683   -0.0393    1.3036    1.7370   -0.1494   -2.0847    0.5510   -0.0473       NaN

>> statsr.df

ans =

  Columns 1 through 10

  417.7926  423.9781  422.9375  423.9781  422.5963  422.5755  419.2995  423.9988  422.7089  304.2942

  Columns 11 through 20

  412.6516  403.8743  385.2267  423.1365  422.5309  396.7257  423.7724  423.9777  420.8204  419.7811

  Columns 21 through 30

  423.7515  423.6452  414.7243  304.7293  341.7154  423.9703       NaN  423.9130  269.9686  415.3424

  Columns 31 through 40

  420.8359  419.9029  420.8390  421.6248  421.6266  270.5086  419.8334  419.6895  408.1686  422.6593

  Columns 41 through 50

  423.5113  422.8458  389.3274  423.7112  423.6242  423.3816  422.5603  399.4475  423.7061  421.7840

  Columns 51 through 60

  423.4015  421.7565  421.8504  401.1543  407.3811  423.6396  421.6604  423.5592  412.7178       NaN

>> statsr.sd

ans =

   1.0e+03 *

  Columns 1 through 10

    0.0014    0.0214    0.1891    0.0214    0.0017    0.0124    0.0016    0.0012    0.0014    0.2592
    0.0013    0.0214    0.1816    0.0214    0.0016    0.0119    0.0014    0.0013    0.0013    0.5483
```

```
  Columns 11 through 20

    0.0174    0.0004    0.5853    0.0165    0.0014    0.0097    0.0014    0.0182    0.0014    0.0014
    0.0207    0.0003    0.4256    0.0174    0.0015    0.0129    0.0014    0.0183    0.0013    0.0013

  Columns 21 through 30

    0.0012    0.0032    0.0109    0.4993    0.0027    0.0256         0    0.0043    0.0045    0.0205
    0.0012    0.0033    0.0128    1.0535    0.0016    0.0256         0    0.0044    0.0121    0.0239

  Columns 31 through 40

    0.0201    0.0201    0.0201    0.0204    0.0204    0.0004    0.0111    0.0109    0.0142    0.0294
    0.0186    0.0224    0.0186    0.0191    0.0191    0.0010    0.0101    0.0100    0.0117    0.0281

  Columns 41 through 50

    0.0059    0.0060    0.0043    0.0101    0.0101    0.0170    0.0312    0.0110    0.0045    0.0080
    0.0057    0.0064    0.0032    0.0104    0.0105    0.0165    0.0297    0.0143    0.0044    0.0087

  Columns 51 through 60

    0.0056    0.0099    0.0096    0.0059    0.0096    0.0050    0.0079    0.0053    0.0074       NaN
    0.0059    0.0093    0.0090    0.0047    0.0079    0.0052    0.0086    0.0052    0.0088       NaN

>> hl

hl =

  Columns 1 through 18

     0     0     0     0     0     0     0     1     0     1     1     0     0     0     0     0     0     0

  Columns 19 through 36

     0     1     0     0     0     1     0     0   NaN     0     1     0     0     1     0     0     0     0

  Columns 37 through 54

     0     0     0     0     0     0     0     0     0     0     0     0     0     0     0     0     0     0

  Columns 55 through 60

     0     0     1     0     0   NaN

>> pl

pl =

  Columns 1 through 10
```

```
    0.0668    0.8714    0.4387    0.1286    0.0526    0.8363    0.9123    0.0053    0.0843    0.0168

  Columns 11 through 20

    0.0155    0.8846    0.4971    0.5368    0.4467    0.6135    0.4872    0.9796    0.8534    0.0314

  Columns 21 through 30

    0.6329    0.2803    0.3771    0.0211    0.7789    0.7264       NaN    0.1848    0.0025    0.0620

  Columns 31 through 40

    0.6069    0.0152    0.3932    0.6960    0.3041    0.1017    0.7073    0.7209    0.6315    0.2859

  Columns 41 through 50

    0.2280    0.5793    0.7966    0.5035    0.5001    0.4488    0.9619    0.5613    0.3022    0.3096

  Columns 51 through 60

    0.4724    0.4332    0.4844    0.9034    0.9584    0.4406    0.0188    0.7090    0.4812       NaN

>> cil

cil =

  Columns 1 through 10

      -Inf      -Inf      -Inf      -Inf      -Inf      -Inf      -Inf      -Inf      -Inf      -Inf
    0.0189    5.7739   26.8438    1.0665    0.0040    3.0975    0.4435   -0.1115    0.0349  -20.1381

  Columns 11 through 20

      -Inf      -Inf      -Inf      -Inf      -Inf      -Inf      -Inf      -Inf      -Inf      -Inf
   -0.9537    0.1015   81.4474    2.8639    0.2116    2.1383    0.2172    6.5379    0.3429   -0.0292

  Columns 21 through 30

      -Inf      -Inf      -Inf      -Inf      -Inf      -Inf      -Inf      -Inf      -Inf      -Inf
    0.2262    0.3324    1.5375  -31.0859    0.5271    5.5865       NaN    0.3195   -1.0372    0.2318

  Columns 31 through 40

      -Inf      -Inf      -Inf      -Inf      -Inf      -Inf      -Inf      -Inf      -Inf      -Inf
    3.5980   -1.0820    2.5809    4.1452    2.1771    0.0277    2.2513    2.2636    2.5052    3.0160

  Columns 41 through 50

      -Inf      -Inf      -Inf      -Inf      -Inf      -Inf      -Inf      -Inf      -Inf      -Inf
```

```
    0.5083    1.1109    0.9062    1.6432    1.6401    2.4648   10.0991    2.2277    0.4910    0.9353

  Columns 51 through 60

     -Inf      -Inf      -Inf      -Inf      -Inf      -Inf      -Inf      -Inf      -Inf      -Inf
    0.8803    1.3829    1.4495    1.5322    2.8896    0.7385   -0.3473    1.1259    1.2584       NaN

>> statsl.tstat

ans =

  Columns 1 through 10

   -1.5031    1.1344   -0.1543   -1.1344   -1.6236    0.9805    1.3576   -2.5658   -1.3788   -2.1352

  Columns 11 through 20

   -2.1636    1.2004   -0.0073    0.0923   -0.1341    0.2887   -0.0321    2.0520    1.0523   -1.8655

  Columns 21 through 30

    0.3399   -0.5825   -0.3134   -2.0396    0.7694    0.6026       NaN   -0.8983   -2.8260   -1.5411

  Columns 31 through 40

    0.2715   -2.1729   -0.2712    0.5133   -0.5131   -1.2749    0.5460    0.5861    0.3360   -0.5659

  Columns 41 through 50

   -0.7463    0.2001    0.8303    0.0087    0.0001   -0.1287    1.7776    0.1544   -0.5186   -0.4975

  Columns 51 through 60

   -0.0693   -0.1683   -0.0393    1.3036    1.7370   -0.1494   -2.0847    0.5510   -0.0473       NaN

>> statsl.df

ans =

  Columns 1 through 10

  417.7926  423.9781  422.9375  423.9781  422.5963  422.5755  419.2995  423.9988  422.7089  304.2942

  Columns 11 through 20

  412.6516  403.8743  385.2267  423.1365  422.5309  396.7257  423.7724  423.9777  420.8204  419.7811

  Columns 21 through 30

  423.7515  423.6452  414.7243  304.7293  341.7154  423.9703       NaN  423.9130  269.9686  415.3424
```

```
  Columns 31 through 40

  420.8359  419.9029  420.8390  421.6248  421.6266  270.5086  419.8334  419.6895  408.1686  422.6593

  Columns 41 through 50

  423.5113  422.8458  389.3274  423.7112  423.6242  423.3816  422.5603  399.4475  423.7061  421.7840

  Columns 51 through 60

  423.4015  421.7565  421.8504  401.1543  407.3811  423.6396  421.6604  423.5592  412.7178       NaN


>> statsl.sd

ans =

   1.0e+03 *

  Columns 1 through 10

    0.0014    0.0214    0.1891    0.0214    0.0017    0.0124    0.0016    0.0012    0.0014    0.2592
    0.0013    0.0214    0.1816    0.0214    0.0016    0.0119    0.0014    0.0013    0.0013    0.5483

  Columns 11 through 20

    0.0174    0.0004    0.5853    0.0165    0.0014    0.0097    0.0014    0.0182    0.0014    0.0014
    0.0207    0.0003    0.4256    0.0174    0.0015    0.0129    0.0014    0.0183    0.0013    0.0013

  Columns 21 through 30

    0.0012    0.0032    0.0109    0.4993    0.0027    0.0256         0    0.0043    0.0045    0.0205
    0.0012    0.0033    0.0128    1.0535    0.0016    0.0256         0    0.0044    0.0121    0.0239

  Columns 31 through 40

    0.0201    0.0201    0.0201    0.0204    0.0204    0.0004    0.0111    0.0109    0.0142    0.0294
    0.0186    0.0224    0.0186    0.0191    0.0191    0.0010    0.0101    0.0100    0.0117    0.0281

  Columns 41 through 50

    0.0059    0.0060    0.0043    0.0101    0.0101    0.0170    0.0312    0.0110    0.0045    0.0080
    0.0057    0.0064    0.0032    0.0104    0.0105    0.0165    0.0297    0.0143    0.0044    0.0087

  Columns 51 through 60

    0.0056    0.0099    0.0096    0.0059    0.0096    0.0050    0.0079    0.0053    0.0074       NaN
    0.0059    0.0093    0.0090    0.0047    0.0079    0.0052    0.0086    0.0052    0.0088       NaN
```

```
>>


>> d

d =

  Columns 1 through 10

   -0.1457    0.1099   -0.0150   -0.1099   -0.1574    0.0950    0.1316   -0.2486   -0.1336   -0.2063

  Columns 11 through 20

   -0.2095    0.1164   -0.0007    0.0089   -0.0130    0.0279   -0.0031    0.1988    0.1020   -0.1808

  Columns 21 through 30

    0.0329   -0.0564   -0.0303   -0.1971    0.0747    0.0584       NaN   -0.0870   -0.2729   -0.1492

  Columns 31 through 40

    0.0263   -0.2104   -0.0263    0.0498   -0.0497   -0.1231    0.0529    0.0568    0.0326   -0.0548

  Columns 41 through 50

   -0.0723    0.0194    0.0806    0.0008    0.0000   -0.0125    0.1723    0.0149   -0.0503   -0.0482

  Columns 51 through 59

   -0.0067   -0.0163   -0.0038    0.1265    0.1685   -0.0145   -0.2019    0.0534   -0.0046

>>
```

Laymen vs. Professionals

```
>> L

L =

  Columns 1 through 10

    0.4662   62.2880  210.9745   37.7120    6.1806   42.0468    2.5926    7.9028    5.8981   82.3796

  Columns 11 through 20

   22.7611    0.3870  339.1255   60.6204    3.6481   20.7620    3.6204   59.9884    2.4259    5.2824

  Columns 21 through 30

    3.7315    4.3148   25.6940  141.2130    1.8981   64.4861    1.0000    4.6528    4.0426    7.8736

  Columns 31 through 40

    9.4148   33.0125   90.5875    7.4634   92.5375    0.1676    4.0000    3.8796    3.4639   88.6616

  Columns 41 through 50

   45.4542   50.9514    3.5968    7.8120   92.1667   20.3880   56.0171   14.9875    1.1190   12.6255

  Columns 51 through 60

    1.3838   14.8458   13.2407    1.5361   13.1042    1.1421   12.4602    1.2366   12.3167       NaN

>> P

P =

  Columns 1 through 10

    1.2524   75.2671  304.4671   24.7329    6.5190   37.5205    2.0667    8.6524    6.1048  241.9571

  Columns 11 through 20

   34.8090    0.4034  319.9724   56.4238    3.3381   16.5186    3.4476   49.3629    2.0381    5.3048

  Columns 21 through 30

    3.4905    3.9810   27.1548  459.6714    2.8143   47.1857    1.0000    6.8095    7.8476   26.2410

  Columns 31 through 40
```

```
   28.0552   40.7181   71.9457   24.5543   75.4462    0.4071   14.3681   14.1162   10.1857   61.3348

  Columns 41 through 50

   48.0024   48.3805    3.6181   14.3500   85.6381   15.7590   66.7319   15.6200    3.8186   10.8590

  Columns 51 through 60

    4.4905   10.9571    9.7000    4.6629   12.9052    4.6000   10.0100    4.8200    7.5467       NaN

>> h

h =

  Columns 1 through 18

     1     1     1     1     1     1     1     1     0     1     1     0     0     1     1     1     0     1

  Columns 19 through 36

     1     0     1     0     0     1     1     1   NaN     1     1     1     1     1     1     1     1     1

  Columns 37 through 54

     1     1     1     1     1     1     0     1     1     1     1     0     1     1     1     1     1     1

  Columns 55 through 60

     0     1     1     1     1   NaN

>> p

p =

  Columns 1 through 10

    0.0000    0.0000    0.0000    0.0000    0.0332    0.0001    0.0003    0.0000    0.1103    0.0002

  Columns 11 through 20

    0.0000    0.6446    0.6898    0.0101    0.0254    0.0001    0.1952    0.0000    0.0020    0.8677

  Columns 21 through 30

    0.0327    0.2861    0.2057    0.0001    0.0000    0.0000       NaN    0.0000    0.0000    0.0000

  Columns 31 through 40

    0.0000    0.0002    0.0000    0.0000    0.0000    0.0011    0.0000    0.0000    0.0000    0.0000
```

```
  Columns 41 through 50

    0.0000    0.0000    0.9533    0.0000    0.0000    0.0040    0.0002    0.6093    0.0000    0.0297

  Columns 51 through 60

    0.0000    0.0000    0.0001    0.0000    0.8163    0.0000    0.0021    0.0000    0.0000       NaN

>> ci

ci =

  Columns 1 through 10

   -1.0343  -16.8441 -127.4908    9.1142   -0.6499    2.2572    0.2401   -0.9780   -0.4604 -241.3875
   -0.5381   -9.1142  -59.4944   16.8441   -0.0270    6.7954    0.8117   -0.5212    0.0472  -77.7676

  Columns 11 through 20

  -15.4950   -0.0862  -75.0995    1.0049    0.0385    2.1155   -0.0890    7.2955    0.1426   -0.2860
   -8.6009    0.0534  113.4056    7.3882    0.5816    6.3714    0.4345   13.9556    0.6330    0.2413

  Columns 21 through 30

    0.0199   -0.2805   -3.7261 -475.4959   -1.3349   12.7183       NaN   -2.9635   -5.5476  -22.1837
    0.4621    0.9483    0.8046 -161.4211   -0.4973   21.8825       NaN   -1.3500   -2.0625  -14.5510

  Columns 31 through 40

  -21.8078  -11.6722   15.4745  -20.4260   13.7563   -0.3822  -12.1353  -11.9806   -8.9754   22.5239
  -15.4731   -3.7390   21.8091  -13.7557   20.4263   -0.0969   -8.6009   -8.4926   -4.4683   32.1297

  Columns 41 through 50

   -3.6225    1.4256   -0.7367   -8.3801    4.6794    1.4848  -16.4018   -3.0632   -3.4806    0.1743
   -1.4740    3.7162    0.6941   -4.6958    8.3777    7.7731   -5.0278    1.7982   -1.9186    3.3585

  Columns 51 through 60

   -4.1700    2.0825    1.8021   -4.0995   -1.4829   -4.3733    0.8904   -4.5471    3.2924       NaN
   -2.0434    5.6949    5.2794   -2.1540    1.8807   -2.5424    4.0099   -2.6197    6.2476       NaN

>> stats.tstat

ans =

  Columns 1 through 10

   -6.2410   -6.6009   -5.4054    6.6009   -2.1362    3.9210    3.6173   -6.4497   -1.6000   -3.8408
```

```
  Columns 11 through 20

   -6.8706   -0.4617    0.3994    2.5861    2.2451    3.9201    1.2980    6.2731    3.1107   -0.1667

  Columns 21 through 30

    2.1439    1.0682   -1.2676   -3.9934   -4.3002    7.4215       NaN   -5.2569   -4.3006   -9.4636

  Columns 31 through 40

  -11.5696   -3.8184   11.5707  -10.0764   10.0770   -3.3037  -11.5362  -11.5413   -5.8660   11.1872

  Columns 41 through 50

   -4.6652    4.4149   -0.0586   -6.9763    6.9398    2.8940   -3.7034   -0.5115   -6.8018    2.1809

  Columns 51 through 60

   -5.7475    4.2324    4.0030   -6.3217    0.2325   -7.4328    3.0877   -7.3184    6.3467       NaN

>> stats.df

ans =

  Columns 1 through 10

  247.8120  421.1950  419.6467  421.1950  423.9979  416.6873  414.4881  423.8976  420.1435  262.6222

  Columns 11 through 20

  411.8252  407.8769  423.3349  345.4193  356.8056  408.2729  349.6014  394.9389  362.4195  378.6862

  Columns 21 through 30

  340.8986  411.7077  406.1502  257.3486  413.3590  421.3262       NaN  374.5004  248.7106  374.2474

  Columns 31 through 40

  401.1507  423.7269  401.1136  374.5946  374.5884  327.6824  376.1750  375.6713  356.3053  378.9181

  Columns 41 through 50

  353.3992  348.8631  421.9465  420.6196  421.0572  413.4553  422.1894  416.2372  307.4682  418.7417

  Columns 51 through 60

  331.9718  406.4755  420.8793  353.3748  417.0100  306.2627  423.9694  292.0078  393.0200       NaN

>> stats.sd
```

```
ans =

   1.0e+03 *

  Columns 1 through 10

    0.0005    0.0214    0.1717    0.0214    0.0017    0.0128    0.0016    0.0012    0.0014    0.2072
    0.0017    0.0192    0.1848    0.0192    0.0016    0.0109    0.0014    0.0012    0.0012    0.5664

  Columns 11 through 20

    0.0167    0.0004    0.4919    0.0206    0.0017    0.0124    0.0017    0.0200    0.0015    0.0016
    0.0193    0.0003    0.4976    0.0119    0.0011    0.0099    0.0010    0.0147    0.0010    0.0011

  Columns 21 through 30

    0.0014    0.0030    0.0107    0.3793    0.0020    0.0253         0    0.0034    0.0038    0.0163
    0.0008    0.0034    0.0129    1.0934    0.0023    0.0227         0    0.0049    0.0122    0.0231

  Columns 31 through 40

    0.0147    0.0209    0.0147    0.0142    0.0142    0.0009    0.0076    0.0075    0.0091    0.0208
    0.0183    0.0208    0.0183    0.0202    0.0202    0.0005    0.0107    0.0105    0.0140    0.0289

  Columns 41 through 50

    0.0069    0.0074    0.0037    0.0094    0.0094    0.0180    0.0312    0.0120    0.0026    0.0080
    0.0041    0.0043    0.0038    0.0100    0.0100    0.0149    0.0284    0.0134    0.0051    0.0087

  Columns 51 through 60

    0.0039    0.0086    0.0089    0.0039    0.0084    0.0030    0.0083    0.0030    0.0089       NaN
    0.0068    0.0103    0.0094    0.0061    0.0093    0.0060    0.0080    0.0065    0.0065       NaN

>>
```

```
>> hr

hr =

  Columns 1 through 18

     0     0     0     1     0     1     1     0     0     0     0     0     0     1     1     1     0     1

  Columns 19 through 36

     1     0     1     0     0     0     0     1   NaN     0     0     0     0     0     1     0     1     0

  Columns 37 through 54

     0     0     0     1     0     1     0     0     1     1     0     0     0     1     0     1     1     0

  Columns 55 through 60

     0     0     1     0     1   NaN

>> pr

pr =

  Columns 1 through 10

    1.0000    1.0000    1.0000    0.0000    0.9834    0.0001    0.0002    1.0000    0.9448    0.9999

  Columns 11 through 20

    1.0000    0.6777    0.3449    0.0051    0.0127    0.0001    0.0976    0.0000    0.0010    0.5662

  Columns 21 through 30

    0.0164    0.1430    0.8972    1.0000    1.0000    0.0000       NaN    1.0000    1.0000    1.0000

  Columns 31 through 40

    1.0000    0.9999    0.0000    1.0000    0.0000    0.9995    1.0000    1.0000    1.0000    0.0000

  Columns 41 through 50

    1.0000    0.0000    0.5234    1.0000    0.0000    0.0020    0.9999    0.6954    1.0000    0.0149

  Columns 51 through 60

    1.0000    0.0000    0.0000    1.0000    0.4081    1.0000    0.0011    1.0000    0.0000       NaN
```

```
>> cir

cir =

  Columns 1 through 10

   -0.9942  -16.2206 -122.0054    9.7378   -0.5997    2.6233    0.2862   -0.9412   -0.4195 -228.1601
       Inf       Inf       Inf       Inf       Inf       Inf       Inf       Inf       Inf       Inf

  Columns 11 through 20

  -14.9388   -0.0749  -59.8930    1.5202    0.0823    2.4589   -0.0468    7.8329    0.1822   -0.2434
       Inf       Inf       Inf       Inf       Inf       Inf       Inf       Inf       Inf       Inf

  Columns 21 through 30

    0.0556   -0.1814   -3.3606 -450.1025   -1.2674   13.4576       NaN   -2.8333   -5.2658  -21.5677
       Inf       Inf       Inf       Inf       Inf       Inf       Inf       Inf       Inf       Inf

  Columns 31 through 40

  -21.2967  -11.0322   15.9856  -19.8877   14.2946   -0.3592  -11.8500  -11.6991   -8.6116   23.2991
       Inf       Inf       Inf       Inf       Inf       Inf       Inf       Inf       Inf       Inf

  Columns 41 through 50

   -3.4490    1.6105   -0.6213   -8.0829    4.9778    1.9921  -15.4842   -2.6710   -3.3544    0.4312
       Inf       Inf       Inf       Inf       Inf       Inf       Inf       Inf       Inf       Inf

  Columns 51 through 60

   -3.9983    2.3740    2.0826   -3.9424   -1.2115   -4.2254    1.1421   -4.3914    3.5308       NaN
       Inf       Inf       Inf       Inf       Inf       Inf       Inf       Inf       Inf       Inf

>> statsr.tstat

ans =

  Columns 1 through 10

   -6.2410   -6.6009   -5.4054    6.6009   -2.1362    3.9210    3.6173   -6.4497   -1.6000   -3.8408

  Columns 11 through 20

   -6.8706   -0.4617    0.3994    2.5861    2.2451    3.9201    1.2980    6.2731    3.1107   -0.1667

  Columns 21 through 30

    2.1439    1.0682   -1.2676   -3.9934   -4.3002    7.4215       NaN   -5.2569   -4.3006   -9.4636
```

```
  Columns 31 through 40

  -11.5696   -3.8184   11.5707  -10.0764   10.0770   -3.3037  -11.5362  -11.5413   -5.8660   11.1872

  Columns 41 through 50

   -4.6652    4.4149   -0.0586   -6.9763    6.9398    2.8940   -3.7034   -0.5115   -6.8018    2.1809

  Columns 51 through 60

   -5.7475    4.2324    4.0030   -6.3217    0.2325   -7.4328    3.0877   -7.3184    6.3467       NaN

>> statsr.df

ans =

  Columns 1 through 10

  247.8120  421.1950  419.6467  421.1950  423.9979  416.6873  414.4881  423.8976  420.1435  262.6222

  Columns 11 through 20

  411.8252  407.8769  423.3349  345.4193  356.8056  408.2729  349.6014  394.9389  362.4195  378.6862

  Columns 21 through 30

  340.8986  411.7077  406.1502  257.3486  413.3590  421.3262       NaN  374.5004  248.7106  374.2474

  Columns 31 through 40

  401.1507  423.7269  401.1136  374.5946  374.5884  327.6824  376.1750  375.6713  356.3053  378.9181

  Columns 41 through 50

  353.3992  348.8631  421.9465  420.6196  421.0572  413.4553  422.1894  416.2372  307.4682  418.7417

  Columns 51 through 60

  331.9718  406.4755  420.8793  353.3748  417.0100  306.2627  423.9694  292.0078  393.0200       NaN

>> statsr.sd

ans =

   1.0e+03 *

  Columns 1 through 10

    0.0005    0.0214    0.1717    0.0214    0.0017    0.0128    0.0016    0.0012    0.0014    0.2072
```

```
    0.0017    0.0192    0.1848    0.0192    0.0016    0.0109    0.0014    0.0012    0.0012    0.5664

  Columns 11 through 20

    0.0167    0.0004    0.4919    0.0206    0.0017    0.0124    0.0017    0.0200    0.0015    0.0016
    0.0193    0.0003    0.4976    0.0119    0.0011    0.0099    0.0010    0.0147    0.0010    0.0011

  Columns 21 through 30

    0.0014    0.0030    0.0107    0.3793    0.0020    0.0253         0    0.0034    0.0038    0.0163
    0.0008    0.0034    0.0129    1.0934    0.0023    0.0227         0    0.0049    0.0122    0.0231

  Columns 31 through 40

    0.0147    0.0209    0.0147    0.0142    0.0142    0.0009    0.0076    0.0075    0.0091    0.0208
    0.0183    0.0208    0.0183    0.0202    0.0202    0.0005    0.0107    0.0105    0.0140    0.0289

  Columns 41 through 50

    0.0069    0.0074    0.0037    0.0094    0.0094    0.0180    0.0312    0.0120    0.0026    0.0080
    0.0041    0.0043    0.0038    0.0100    0.0100    0.0149    0.0284    0.0134    0.0051    0.0087

  Columns 51 through 60

    0.0039    0.0086    0.0089    0.0039    0.0084    0.0030    0.0083    0.0030    0.0089       NaN
    0.0068    0.0103    0.0094    0.0061    0.0093    0.0060    0.0080    0.0065    0.0065       NaN

>> hl

hl =

  Columns 1 through 18

     1     1     1     0     1     0     0     1     0     1     1     0     0     0     0     0     0     0

  Columns 19 through 36

     0     0     0     0     0     1     1     0   NaN     1     1     1     1     1     0     1     0     1

  Columns 37 through 54

     1     1     1     0     1     0     0     1     0     0     1     0     1     0     1     0     0     1

  Columns 55 through 60

     0     1     0     1     0   NaN

>> pl

pl =
```

```
  Columns 1 through 10

    0.0000    0.0000    0.0000    1.0000    0.0166    0.9999    0.9998    0.0000    0.0552    0.0001

  Columns 11 through 20

    0.0000    0.3223    0.6551    0.9949    0.9873    0.9999    0.9024    1.0000    0.9990    0.4338

  Columns 21 through 30

    0.9836    0.8570    0.1028    0.0000    0.0000    1.0000       NaN    0.0000    0.0000    0.0000

  Columns 31 through 40

    0.0000    0.0001    1.0000    0.0000    1.0000    0.0005    0.0000    0.0000    0.0000    1.0000

  Columns 41 through 50

    0.0000    1.0000    0.4766    0.0000    1.0000    0.9980    0.0001    0.3046    0.0000    0.9851

  Columns 51 through 60

    0.0000    1.0000    1.0000    0.0000    0.5919    0.0000    0.9989    0.0000    1.0000       NaN

>> cil

cil =

  Columns 1 through 10

     -Inf      -Inf      -Inf      -Inf      -Inf      -Inf      -Inf      -Inf      -Inf      -Inf
   -0.5782   -9.7378  -64.9798   16.2206   -0.0773    6.4293    0.7656   -0.5580    0.0063  -90.9949

  Columns 11 through 20

     -Inf      -Inf      -Inf      -Inf      -Inf      -Inf      -Inf      -Inf      -Inf      -Inf
   -9.1571    0.0421   98.1991    6.8729    0.5378    6.0280    0.3923   13.4182    0.5934    0.1987

  Columns 21 through 30

     -Inf      -Inf      -Inf      -Inf      -Inf      -Inf      -Inf      -Inf      -Inf      -Inf
    0.4264    0.8491    0.4390 -186.8144   -0.5649   21.1432       NaN   -1.4802   -2.3443  -15.1670

  Columns 31 through 40

     -Inf      -Inf      -Inf      -Inf      -Inf      -Inf      -Inf      -Inf      -Inf      -Inf
  -15.9842   -4.3790   21.2980  -14.2941   19.8880   -0.1199   -8.8861   -8.7741   -4.8321   31.3545

  Columns 41 through 50
```

```
     -Inf     -Inf     -Inf     -Inf     -Inf     -Inf     -Inf     -Inf     -Inf     -Inf
   -1.6474    3.5313    0.5786   -4.9931    8.0794    7.2657   -5.9453    1.4060   -2.0448    3.1016

  Columns 51 through 60

     -Inf     -Inf     -Inf     -Inf     -Inf     -Inf     -Inf     -Inf     -Inf     -Inf
   -2.2151    5.4034    4.9989   -2.3111    1.6094   -2.6903    3.7583   -2.7755    6.0092       NaN

>> statsl.tstat

ans =

  Columns 1 through 10

   -6.2410   -6.6009   -5.4054    6.6009   -2.1362    3.9210    3.6173   -6.4497   -1.6000   -3.8408

  Columns 11 through 20

   -6.8706   -0.4617    0.3994    2.5861    2.2451    3.9201    1.2980    6.2731    3.1107   -0.1667

  Columns 21 through 30

    2.1439    1.0682   -1.2676   -3.9934   -4.3002    7.4215       NaN   -5.2569   -4.3006   -9.4636

  Columns 31 through 40

  -11.5696   -3.8184   11.5707  -10.0764   10.0770   -3.3037  -11.5362  -11.5413   -5.8660   11.1872

  Columns 41 through 50

   -4.6652    4.4149   -0.0586   -6.9763    6.9398    2.8940   -3.7034   -0.5115   -6.8018    2.1809

  Columns 51 through 60

   -5.7475    4.2324    4.0030   -6.3217    0.2325   -7.4328    3.0877   -7.3184    6.3467       NaN

>> statsl.df

ans =

  Columns 1 through 10

  247.8120  421.1950  419.6467  421.1950  423.9979  416.6873  414.4881  423.8976  420.1435  262.6222

  Columns 11 through 20

  411.8252  407.8769  423.3349  345.4193  356.8056  408.2729  349.6014  394.9389  362.4195  378.6862

  Columns 21 through 30
```

340.8986 411.7077 406.1502 257.3486 413.3590 421.3262   NaN 374.5004 248.7106 374.2474

Columns 31 through 40

401.1507 423.7269 401.1136 374.5946 374.5884 327.6824 376.1750 375.6713 356.3053 378.9181

Columns 41 through 50

353.3992 348.8631 421.9465 420.6196 421.0572 413.4553 422.1894 416.2372 307.4682 418.7417

Columns 51 through 60

331.9718 406.4755 420.8793 353.3748 417.0100 306.2627 423.9694 292.0078 393.0200   NaN

```
>> statsl.sd

ans =

   1.0e+03 *

  Columns 1 through 10

    0.0005    0.0214    0.1717    0.0214    0.0017    0.0128    0.0016    0.0012    0.0014    0.2072
    0.0017    0.0192    0.1848    0.0192    0.0016    0.0109    0.0014    0.0012    0.0012    0.5664

  Columns 11 through 20

    0.0167    0.0004    0.4919    0.0206    0.0017    0.0124    0.0017    0.0200    0.0015    0.0016
    0.0193    0.0003    0.4976    0.0119    0.0011    0.0099    0.0010    0.0147    0.0010    0.0011

  Columns 21 through 30

    0.0014    0.0030    0.0107    0.3793    0.0020    0.0253         0    0.0034    0.0038    0.0163
    0.0008    0.0034    0.0129    1.0934    0.0023    0.0227         0    0.0049    0.0122    0.0231

  Columns 31 through 40

    0.0147    0.0209    0.0147    0.0142    0.0142    0.0009    0.0076    0.0075    0.0091    0.0208
    0.0183    0.0208    0.0183    0.0202    0.0202    0.0005    0.0107    0.0105    0.0140    0.0289

  Columns 41 through 50

    0.0069    0.0074    0.0037    0.0094    0.0094    0.0180    0.0312    0.0120    0.0026    0.0080
    0.0041    0.0043    0.0038    0.0100    0.0100    0.0149    0.0284    0.0134    0.0051    0.0087

  Columns 51 through 60

    0.0039    0.0086    0.0089    0.0039    0.0084    0.0030    0.0083    0.0030    0.0089       NaN
    0.0068    0.0103    0.0094    0.0061    0.0093    0.0060    0.0080    0.0065    0.0065       NaN
```

```
>> d

d =

  Columns 1 through 10

   -0.6119   -0.6387   -0.5244    0.6387   -0.2069    0.3791    0.3497   -0.6247   -0.1548   -0.3762

  Columns 11 through 20

   -0.6672   -0.0446    0.0387    0.2489    0.2162    0.3787    0.1249    0.6054    0.2996   -0.0161

  Columns 21 through 30

    0.2063    0.1037   -0.1232   -0.3913   -0.4175    0.7181       NaN   -0.5119   -0.4216   -0.9215

  Columns 31 through 40

   -1.1246   -0.3700    1.1247   -0.9812    0.9812   -0.3176   -1.1232   -1.1237   -0.5718    1.0890

  Columns 41 through 50

   -0.4491    0.4249   -0.0057   -0.6767    0.6731    0.2797   -0.3584   -0.0496   -0.6647    0.2116

  Columns 51 through 59

   -0.5610    0.4112    0.3882   -0.6163    0.0226   -0.7264    0.2991   -0.7158    0.6124

>>
```

Young vs. Old

Y =

Columns 1 through 10

0.8306	69.6402	224.6469	30.3598	6.3923	40.8909	2.2632	8.1722	6.0191	182.7416

Columns 11 through 20

27.2809	0.3845	288.0756	58.7081	3.4928	18.5545	3.5120	54.4775	2.2967	5.3349

Columns 21 through 30

3.6364	4.2344	26.0536	337.8852	2.0000	58.8024	1.0000	5.0670	6.2115	14.0493

Columns 31 through 40

15.0373	36.3943	84.9641	12.1986	87.8019	0.2522	7.2732	7.0981	4.9263	80.7110

Columns 41 through 50

46.8952	49.6177	3.4885	10.3928	89.5823	16.5947	58.9713	14.4828	1.7627	12.2120

Columns 51 through 60

2.3177	14.5019	10.9962	2.5852	13.5837	2.0335	11.8627	2.2325	11.4321	NaN

>> O

O =

Columns 1 through 10

0.8820	67.5207	291.0194	32.4793	6.3318	38.7166	2.3917	8.4009	6.0092	142.4700

Columns 11 through 20

30.4249	0.4056	383.1138	58.6498	3.5161	18.8083	3.5668	55.1161	2.1659	5.2673

Columns 21 through 30

3.5991	4.0737	26.7756	263.9585	2.6959	53.1281	1.0000	6.4147	5.6608	20.1908

Columns 31 through 40

22.2935	37.4788	77.7083	19.7046	80.2963	0.3092	10.7747	10.5871	8.9290	69.7097

```
  Columns 41 through 50

   46.5516   49.6959    3.7535   11.7189   88.2724   19.5046   63.1065   15.9230    3.2129   11.3028

  Columns 51 through 60

    3.4359   11.4751   11.9535    3.6972   12.4065    3.6535   10.6060    3.7157    8.6046       NaN

>> h

h =

  Columns 1 through 18

     0     0     1     0     0     0     0     0     0     0     0     0     0     0     0     0     0     0

  Columns 19 through 36

     0     0     0     0     0     0     1     1   NaN     1     0     1     1     0     1     1     1     0

  Columns 37 through 54

     1     1     1     1     0     0     0     0     0     0     0     0     1     0     1     1     0     1

  Columns 55 through 60

     0     1     0     1     1   NaN

>> p

p =

  Columns 1 through 10

    0.6934    0.3069    0.0002    0.3069    0.7048    0.0648    0.3836    0.0610    0.9389    0.3421

  Columns 11 through 20

    0.0912    0.5521    0.0525    0.9718    0.8681    0.8176    0.6845    0.7193    0.3046    0.6170

  Columns 21 through 30

    0.7445    0.6061    0.5306    0.3641    0.0012    0.0222       NaN    0.0014    0.5431    0.0043

  Columns 31 through 40

    0.0001    0.6013    0.0001    0.0001    0.0001    0.4473    0.0006    0.0005    0.0013    0.0001

  Columns 41 through 50
```

```
    0.5411    0.8964    0.4666    0.1803    0.1872    0.0711    0.1615    0.2430    0.0007    0.2625

  Columns 51 through 60

    0.0442    0.0012    0.2858    0.0314    0.1703    0.0009    0.1160    0.0034    0.0003       NaN

>> ci

ci =

  Columns 1 through 10

   -0.3075   -1.9527 -100.8163   -6.1916   -0.2534   -0.1338   -0.4183   -0.4680   -0.2442  -43.0220
    0.2047    6.1916  -31.9286    1.9527    0.3745    4.4825    0.1612    0.0106    0.2641  123.5651

  Columns 11 through 20

   -6.7944   -0.0908 -191.1231   -3.1887   -0.2991   -2.4152   -0.3201   -4.1283   -0.1193   -0.1980
    0.5064    0.0486    1.0466    3.3054    0.2525    1.9077    0.2104    2.8511    0.3808    0.3333

  Columns 21 through 30

   -0.1875   -0.4514   -2.9830  -86.1265   -1.1140    0.8143       NaN   -2.1701   -1.2291  -10.3519
    0.2620    0.7729    1.5390  233.9797   -0.2777   10.5343       NaN   -0.5254    2.3304   -1.9311

  Columns 31 through 40

  -10.8612   -5.1611    3.6508  -11.1914    3.8203   -0.2046   -5.4859   -5.4479   -6.4360    5.6421
   -3.6512    2.9920   10.8608   -3.8207   11.1909    0.0905   -1.5170   -1.5302   -1.5695   16.3605

  Columns 41 through 50

   -0.7605   -1.2567   -0.9797   -3.2683   -0.6391   -6.0709   -9.9311   -3.8616   -2.2878   -0.6836
    1.4477    1.1004    0.4498    0.6162    3.2590    0.2511    1.6608    0.9811   -0.6126    2.5020

  Columns 51 through 60

   -2.2072    1.2060   -2.7180   -2.1243   -0.5076   -2.5716   -0.3115   -2.4741    1.3044       NaN
   -0.0293    4.8476    0.8034   -0.0999    2.8621   -0.6683    2.8248   -0.4922    4.3505       NaN

>> stats.tstat

ans =

  Columns 1 through 10

   -0.3945    1.0230   -3.7898   -1.0230    0.3791    1.8516   -0.8722   -1.8783    0.0767    0.9516

  Columns 11 through 20
```

```
   -1.6929   -0.5952   -1.9455    0.0353   -0.1661   -0.2308   -0.4066   -0.3597    1.0279    0.5005

  Columns 21 through 30

    0.3262    0.5160   -0.6276    0.9091   -3.2742    2.2950       NaN   -3.2226    0.6089   -2.8671

  Columns 31 through 40

   -3.9566   -0.5229    3.9564   -4.0037    4.0036   -0.7610   -3.4681   -3.5011   -3.2346    4.0351

  Columns 41 through 50

    0.6117   -0.1303   -0.7287   -1.3420    1.3211   -1.8097   -1.4024   -1.1692   -3.4042    1.1220

  Columns 51 through 60

   -2.0185    3.2680   -1.0688   -2.1597    1.3736   -3.3465    1.5752   -2.9425    3.6490       NaN

>> stats.df

ans =

  Columns 1 through 10

  420.6534  422.1926  351.7396  422.1926  414.4241  423.8548  422.4341  421.6016  421.3924  288.4702

  Columns 11 through 20

  423.6512  397.7719  339.8302  403.5670  405.7895  400.1669  402.1948  423.5199  416.0716  414.2345

  Columns 21 through 30

  393.0563  422.5145  423.0120  288.6391  318.4177  416.4523       NaN  373.7940  293.8197  423.6941

  Columns 31 through 40

  414.9638  423.9874  414.9654  408.8514  408.8435  305.5234  421.5183  420.8847  377.0153  416.6657

  Columns 41 through 50

  415.5922  418.8838  408.7500  422.8181  423.0525  404.5937  423.8907  419.0496  382.1155  421.6371

  Columns 51 through 60

  422.4259  399.2492  409.9297  414.8662  403.2914  402.1315  423.9925  392.8391  422.6070       NaN

>> sd
Undefined function or variable 'sd'.

>> stats.sd
```

```
ans =

   1.0e+03 *

  Columns 1 through 10

    0.0014    0.0203    0.1301    0.0203    0.0015    0.0120    0.0014    0.0012    0.0013    0.5585
    0.0013    0.0225    0.2213    0.0225    0.0018    0.0122    0.0016    0.0013    0.0014    0.2546

  Columns 11 through 20

    0.0191    0.0003    0.3472    0.0185    0.0016    0.0096    0.0015    0.0183    0.0014    0.0015
    0.0192    0.0004    0.6266    0.0153    0.0013    0.0129    0.0012    0.0184    0.0012    0.0013

  Columns 21 through 30

    0.0013    0.0031    0.0114    1.0729    0.0014    0.0267         0    0.0034    0.0119    0.0214
    0.0010    0.0034    0.0124    0.4897    0.0028    0.0242         0    0.0051    0.0056    0.0228

  Columns 31 through 40

    0.0171    0.0209    0.0171    0.0170    0.0170    0.0010    0.0098    0.0096    0.0100    0.0257
    0.0206    0.0219    0.0206    0.0215    0.0215    0.0005    0.0110    0.0109    0.0151    0.0305

  Columns 41 through 50

    0.0053    0.0057    0.0033    0.0097    0.0098    0.0144    0.0296    0.0118    0.0035    0.0079
    0.0063    0.0066    0.0042    0.0107    0.0107    0.0186    0.0312    0.0136    0.0052    0.0088

  Columns 51 through 60

    0.0054    0.0105    0.0082    0.0048    0.0096    0.0043    0.0081    0.0043    0.0081       NaN
    0.0060    0.0085    0.0102    0.0058    0.0079    0.0056    0.0084    0.0060    0.0079       NaN

>> hr

hr =

  Columns 1 through 18

     0     0     0     0     0     1     0     0     0     0     0     0     0     0     0     0     0     0

  Columns 19 through 36

     0     0     0     0     0     0     0     1   NaN     0     0     0     0     0     1     0     1     0

  Columns 37 through 54

     0     0     0     1     0     0     0     0     0     0     0     0     0     0     0     1     0     0
```

```
  Columns 55 through 60

     0     0     0     0     1   NaN

>> pr

pr =

  Columns 1 through 10

    0.6533    0.1534    0.9999    0.8466    0.3524    0.0324    0.8082    0.9695    0.4694    0.1710

  Columns 11 through 20

    0.9544    0.7240    0.9737    0.4859    0.5659    0.5912    0.6578    0.6404    0.1523    0.3085

  Columns 21 through 30

    0.3722    0.3030    0.7347    0.1820    0.9994    0.0111       NaN    0.9993    0.2715    0.9978

  Columns 31 through 40

    1.0000    0.6994    0.0000    1.0000    0.0000    0.7764    0.9997    0.9997    0.9993    0.0000

  Columns 41 through 50

    0.2705    0.5518    0.7667    0.9098    0.0936    0.9645    0.9192    0.8785    0.9996    0.1313

  Columns 51 through 60

    0.9779    0.0006    0.8571    0.9843    0.0852    0.9996    0.0580    0.9983    0.0001       NaN

>> cir

cir =

  Columns 1 through 10

   -0.2662   -1.2957  -95.2552   -5.5346   -0.2028    0.2386   -0.3715   -0.4294   -0.2032  -29.5616
       Inf       Inf       Inf       Inf       Inf       Inf       Inf       Inf       Inf       Inf

  Columns 11 through 20

   -6.2055   -0.0795 -175.6076   -2.6647   -0.2546   -2.0664   -0.2773   -3.5653   -0.0789   -0.1552
       Inf       Inf       Inf       Inf       Inf       Inf       Inf       Inf       Inf       Inf

  Columns 21 through 30

   -0.1512   -0.3527   -2.6182  -60.2616   -1.0464    1.5984       NaN   -2.0374   -0.9415   -9.6726
```

```
     Inf       Inf       Inf       Inf       Inf       Inf       Inf       Inf       Inf       Inf

  Columns 31 through 40

  -10.2796   -4.5034    4.2325  -10.5968    4.4149   -0.1808   -5.1658   -5.1318   -6.0432    6.5068
     Inf       Inf       Inf       Inf       Inf       Inf       Inf       Inf       Inf       Inf

  Columns 41 through 50

   -0.5824   -1.0665   -0.8644   -2.9549   -0.3246   -5.5608   -8.9960   -3.4710   -2.1527   -0.4266
     Inf       Inf       Inf       Inf       Inf       Inf       Inf       Inf       Inf       Inf

  Columns 51 through 60

   -2.0315    1.4998   -2.4339   -1.9609   -0.2357   -2.4180   -0.0585   -2.3142    1.5501       NaN
     Inf       Inf       Inf       Inf       Inf       Inf       Inf       Inf       Inf       Inf

>>
>> cir

cir =

  Columns 1 through 10

   -0.2662   -1.2957  -95.2552   -5.5346   -0.2028    0.2386   -0.3715   -0.4294   -0.2032  -29.5616
     Inf       Inf       Inf       Inf       Inf       Inf       Inf       Inf       Inf       Inf

  Columns 11 through 20

   -6.2055   -0.0795 -175.6076   -2.6647   -0.2546   -2.0664   -0.2773   -3.5653   -0.0789   -0.1552
     Inf       Inf       Inf       Inf       Inf       Inf       Inf       Inf       Inf       Inf

  Columns 21 through 30

   -0.1512   -0.3527   -2.6182  -60.2616   -1.0464    1.5984       NaN   -2.0374   -0.9415   -9.6726
     Inf       Inf       Inf       Inf       Inf       Inf       Inf       Inf       Inf       Inf

  Columns 31 through 40

  -10.2796   -4.5034    4.2325  -10.5968    4.4149   -0.1808   -5.1658   -5.1318   -6.0432    6.5068
     Inf       Inf       Inf       Inf       Inf       Inf       Inf       Inf       Inf       Inf

  Columns 41 through 50

   -0.5824   -1.0665   -0.8644   -2.9549   -0.3246   -5.5608   -8.9960   -3.4710   -2.1527   -0.4266
     Inf       Inf       Inf       Inf       Inf       Inf       Inf       Inf       Inf       Inf

  Columns 51 through 60

   -2.0315    1.4998   -2.4339   -1.9609   -0.2357   -2.4180   -0.0585   -2.3142    1.5501       NaN
```

```
       Inf       Inf       Inf       Inf       Inf       Inf       Inf       Inf       Inf       Inf

>> statsr.tstat

ans =

  Columns 1 through 10

   -0.3945    1.0230   -3.7898   -1.0230    0.3791    1.8516   -0.8722   -1.8783    0.0767    0.9516

  Columns 11 through 20

   -1.6929   -0.5952   -1.9455    0.0353   -0.1661   -0.2308   -0.4066   -0.3597    1.0279    0.5005

  Columns 21 through 30

    0.3262    0.5160   -0.6276    0.9091   -3.2742    2.2950       NaN   -3.2226    0.6089   -2.8671

  Columns 31 through 40

   -3.9566   -0.5229    3.9564   -4.0037    4.0036   -0.7610   -3.4681   -3.5011   -3.2346    4.0351

  Columns 41 through 50

    0.6117   -0.1303   -0.7287   -1.3420    1.3211   -1.8097   -1.4024   -1.1692   -3.4042    1.1220

  Columns 51 through 60

   -2.0185    3.2680   -1.0688   -2.1597    1.3736   -3.3465    1.5752   -2.9425    3.6490       NaN

>> statsr.df

ans =

  Columns 1 through 10

  420.6534  422.1926  351.7396  422.1926  414.4241  423.8548  422.4341  421.6016  421.3924  288.4702

  Columns 11 through 20

  423.6512  397.7719  339.8302  403.5670  405.7895  400.1669  402.1948  423.5199  416.0716  414.2345

  Columns 21 through 30

  393.0563  422.5145  423.0120  288.6391  318.4177  416.4523       NaN  373.7940  293.8197  423.6941

  Columns 31 through 40

  414.9638  423.9874  414.9654  408.8514  408.8435  305.5234  421.5183  420.8847  377.0153  416.6657
```

```
  Columns 41 through 50

  415.5922  418.8838  408.7500  422.8181  423.0525  404.5937  423.8907  419.0496  382.1155  421.6371

  Columns 51 through 60

  422.4259  399.2492  409.9297  414.8662  403.2914  402.1315  423.9925  392.8391  422.6070       NaN

>> statsr.sd

ans =

   1.0e+03 *

  Columns 1 through 10

    0.0014    0.0203    0.1301    0.0203    0.0015    0.0120    0.0014    0.0012    0.0013    0.5585
    0.0013    0.0225    0.2213    0.0225    0.0018    0.0122    0.0016    0.0013    0.0014    0.2546

  Columns 11 through 20

    0.0191    0.0003    0.3472    0.0185    0.0016    0.0096    0.0015    0.0183    0.0014    0.0015
    0.0192    0.0004    0.6266    0.0153    0.0013    0.0129    0.0012    0.0184    0.0012    0.0013

  Columns 21 through 30

    0.0013    0.0031    0.0114    1.0729    0.0014    0.0267         0    0.0034    0.0119    0.0214
    0.0010    0.0034    0.0124    0.4897    0.0028    0.0242         0    0.0051    0.0056    0.0228

  Columns 31 through 40

    0.0171    0.0209    0.0171    0.0170    0.0170    0.0010    0.0098    0.0096    0.0100    0.0257
    0.0206    0.0219    0.0206    0.0215    0.0215    0.0005    0.0110    0.0109    0.0151    0.0305

  Columns 41 through 50

    0.0053    0.0057    0.0033    0.0097    0.0098    0.0144    0.0296    0.0118    0.0035    0.0079
    0.0063    0.0066    0.0042    0.0107    0.0107    0.0186    0.0312    0.0136    0.0052    0.0088

  Columns 51 through 60

    0.0054    0.0105    0.0082    0.0048    0.0096    0.0043    0.0081    0.0043    0.0081       NaN
    0.0060    0.0085    0.0102    0.0058    0.0079    0.0056    0.0084    0.0060    0.0079       NaN

>> hl

hl =

  Columns 1 through 18
```

```
     0     0     1     0     0     0     0     1     0     0     1     0     1     0     0     0     0     0

  Columns 19 through 36

     0     0     0     0     0     0     1     0   NaN     1     0     1     1     0     0     1     0     0

  Columns 37 through 54

     1     1     1     0     0     0     0     0     0     1     0     0     1     0     1     0     0     1

  Columns 55 through 60

     0     1     0     1     0   NaN

>> pl

pl =

  Columns 1 through 10

    0.3467    0.8466    0.0001    0.1534    0.6476    0.9676    0.1918    0.0305    0.5306    0.8290

  Columns 11 through 20

    0.0456    0.2760    0.0263    0.5141    0.4341    0.4088    0.3422    0.3596    0.8477    0.6915

  Columns 21 through 30

    0.6278    0.6970    0.2653    0.8180    0.0006    0.9889       NaN    0.0007    0.7285    0.0022

  Columns 31 through 40

    0.0000    0.3006    1.0000    0.0000    1.0000    0.2236    0.0003    0.0003    0.0007    1.0000

  Columns 41 through 50

    0.7295    0.4482    0.2333    0.0902    0.9064    0.0355    0.0808    0.1215    0.0004    0.8687

  Columns 51 through 60

    0.0221    0.9994    0.1429    0.0157    0.9148    0.0004    0.9420    0.0017    0.9999       NaN

>> cil

cil =

  Columns 1 through 10

      -Inf      -Inf      -Inf      -Inf      -Inf      -Inf      -Inf      -Inf      -Inf      -Inf
    0.1634    5.5346  -37.4897    1.2957    0.3239    4.1101    0.1144   -0.0280    0.2231  110.1047
```

```
  Columns 11 through 20

    -Inf      -Inf      -Inf      -Inf      -Inf      -Inf      -Inf      -Inf      -Inf      -Inf
   -0.0826    0.0373  -14.4688    2.7814    0.2080    1.5589    0.1676    2.2881    0.3404    0.2905

  Columns 21 through 30

    -Inf      -Inf      -Inf      -Inf      -Inf      -Inf      -Inf      -Inf      -Inf      -Inf
    0.2258    0.6741    1.1742  208.1149   -0.3453    9.7501       NaN   -0.6581    2.0428   -2.6104

  Columns 31 through 40

    -Inf      -Inf      -Inf      -Inf      -Inf      -Inf      -Inf      -Inf      -Inf      -Inf
   -4.2329    2.3343   10.2791   -4.4153   10.5963    0.0667   -1.8371   -1.8462   -1.9622   15.4958

  Columns 41 through 50

    -Inf      -Inf      -Inf      -Inf      -Inf      -Inf      -Inf      -Inf      -Inf      -Inf
    1.2696    0.9102    0.3345    0.3028    2.9445   -0.2589    0.7257    0.5904   -0.7478    2.2450

  Columns 51 through 60

    -Inf      -Inf      -Inf      -Inf      -Inf      -Inf      -Inf      -Inf      -Inf      -Inf
   -0.2050    4.5538    0.5193   -0.2632    2.5903   -0.8219    2.5718   -0.6521    4.1048       NaN

>> statsl.tstat

ans =

  Columns 1 through 10

   -0.3945    1.0230   -3.7898   -1.0230    0.3791    1.8516   -0.8722   -1.8783    0.0767    0.9516

  Columns 11 through 20

   -1.6929   -0.5952   -1.9455    0.0353   -0.1661   -0.2308   -0.4066   -0.3597    1.0279    0.5005

  Columns 21 through 30

    0.3262    0.5160   -0.6276    0.9091   -3.2742    2.2950       NaN   -3.2226    0.6089   -2.8671

  Columns 31 through 40

   -3.9566   -0.5229    3.9564   -4.0037    4.0036   -0.7610   -3.4681   -3.5011   -3.2346    4.0351

  Columns 41 through 50

    0.6117   -0.1303   -0.7287   -1.3420    1.3211   -1.8097   -1.4024   -1.1692   -3.4042    1.1220
```

```
  Columns 51 through 60

   -2.0185    3.2680   -1.0688   -2.1597    1.3736   -3.3465    1.5752   -2.9425    3.6490       NaN

>> statsl.df

ans =

  Columns 1 through 10

  420.6534  422.1926  351.7396  422.1926  414.4241  423.8548  422.4341  421.6016  421.3924  288.4702

  Columns 11 through 20

  423.6512  397.7719  339.8302  403.5670  405.7895  400.1669  402.1948  423.5199  416.0716  414.2345

  Columns 21 through 30

  393.0563  422.5145  423.0120  288.6391  318.4177  416.4523       NaN  373.7940  293.8197  423.6941

  Columns 31 through 40

  414.9638  423.9874  414.9654  408.8514  408.8435  305.5234  421.5183  420.8847  377.0153  416.6657

  Columns 41 through 50

  415.5922  418.8838  408.7500  422.8181  423.0525  404.5937  423.8907  419.0496  382.1155  421.6371

  Columns 51 through 60

  422.4259  399.2492  409.9297  414.8662  403.2914  402.1315  423.9925  392.8391  422.6070       NaN

>> statsl.sd

ans =

   1.0e+03 *

  Columns 1 through 10

    0.0014    0.0203    0.1301    0.0203    0.0015    0.0120    0.0014    0.0012    0.0013    0.5585
    0.0013    0.0225    0.2213    0.0225    0.0018    0.0122    0.0016    0.0013    0.0014    0.2546

  Columns 11 through 20

    0.0191    0.0003    0.3472    0.0185    0.0016    0.0096    0.0015    0.0183    0.0014    0.0015
    0.0192    0.0004    0.6266    0.0153    0.0013    0.0129    0.0012    0.0184    0.0012    0.0013

  Columns 21 through 30
```

```
    0.0013    0.0031    0.0114    1.0729    0.0014    0.0267         0    0.0034    0.0119    0.0214
    0.0010    0.0034    0.0124    0.4897    0.0028    0.0242         0    0.0051    0.0056    0.0228

  Columns 31 through 40

    0.0171    0.0209    0.0171    0.0170    0.0170    0.0010    0.0098    0.0096    0.0100    0.0257
    0.0206    0.0219    0.0206    0.0215    0.0215    0.0005    0.0110    0.0109    0.0151    0.0305

  Columns 41 through 50

    0.0053    0.0057    0.0033    0.0097    0.0098    0.0144    0.0296    0.0118    0.0035    0.0079
    0.0063    0.0066    0.0042    0.0107    0.0107    0.0186    0.0312    0.0136    0.0052    0.0088

  Columns 51 through 60

    0.0054    0.0105    0.0082    0.0048    0.0096    0.0043    0.0081    0.0043    0.0081       NaN
    0.0060    0.0085    0.0102    0.0058    0.0079    0.0056    0.0084    0.0060    0.0079       NaN


>> d

d =

  Columns 1 through 10

   -0.0383    0.0990   -0.3640   -0.0990    0.0366    0.1794   -0.0844   -0.1817    0.0074    0.0934

  Columns 11 through 20

   -0.1640   -0.0574   -0.1867    0.0034   -0.0162   -0.0222   -0.0396   -0.0349    0.0998    0.0486

  Columns 21 through 30

    0.0318    0.0499   -0.0607    0.0892   -0.3137    0.2228       NaN   -0.3100    0.0597   -0.2775

  Columns 31 through 40

   -0.3821   -0.0506    0.3821   -0.3864    0.3863   -0.0746   -0.3354   -0.3385   -0.3112    0.3898

  Columns 41 through 50

    0.0591   -0.0126   -0.0703   -0.1298    0.1278   -0.1745   -0.1358   -0.1130   -0.3277    0.1085

  Columns 51 through 59

   -0.1953    0.3180   -0.1031   -0.2086    0.1336   -0.3227    0.1525   -0.2835    0.3538

>>
```

Lay Females vs. Lay Males

```
>> F

F =

  Columns 1 through 10

    0.4111   66.5931  201.9500   33.4069    5.6944   40.7750    2.4861    7.6250    5.4861   52.4167

  Columns 11 through 20

   21.7542    0.4619  336.4472   61.2500    3.6944   19.2958    3.6389   59.6222    2.4306    5.0833

  Columns 21 through 30

    3.7083    4.3333   24.6153   92.3472    1.9028   62.4431    1.0000    4.4583    3.1472    7.3958

  Columns 31 through 40

   10.7069   31.6333   89.2972    8.1361   91.8653    0.1278    4.5944    4.5125    3.5417   87.3556

  Columns 41 through 50

   46.3958   50.3486    3.2597   10.0722   89.9292   18.3056   59.5069   14.4958    1.4292   13.4556

  Columns 51 through 60

    1.2569   14.8069   13.4236    1.7069   12.7667    1.3194   12.4431    1.1986   11.6833       NaN

>> M

M =

  Columns 1 through 10

    0.4472   55.6514  189.0681   44.3486    6.4583   41.9167    2.5972    8.0694    6.1250   82.9722

  Columns 11 through 20

   22.4181    0.3119  377.3750   62.1250    3.8333   21.9361    3.7639   60.2181    2.4722    5.3472

  Columns 21 through 30

    3.8472    4.0694   26.3597  136.3056    1.6111   67.6542    1.0000    4.5000    4.8042    5.4236

  Columns 31 through 40
```

    5.8514   34.1333   94.1500   4.6569   95.3444   0.0708   2.3361   2.1514   2.3208   93.1958

  Columns 41 through 50

   45.4278   51.3361   3.2347   4.8111   95.1889   24.5875   53.7208   17.4903   0.7111   12.8250

  Columns 51 through 60

    1.0736   13.2333   13.6931   0.4986   13.0347   0.8000   12.4347   0.8667   13.3292   NaN

>> h

h =

  Columns 1 through 18

     0   1   0   1   1   0   0   1   1   1   0   1   0   0   0   0   0   0

  Columns 19 through 36

     0   0   0   0   0   0   0   0   NaN   0   1   0   1   0   1   0   0   0

  Columns 37 through 54

     0   1   0   0   0   0   0   1   1   1   0   0   0   0   0   0   0   1

  Columns 55 through 60

     0   0   0   0   0   NaN

>> p

p =

  Columns 1 through 10

    0.5358   0.0030   0.5673   0.0030   0.0029   0.5759   0.6697   0.0185   0.0029   0.0274

  Columns 11 through 20

    0.7899   0.0492   0.6655   0.7939   0.6226   0.2332   0.6489   0.8587   0.8671   0.3346

  Columns 21 through 30

    0.5482   0.6025   0.3643   0.0628   0.2975   0.2149   NaN   0.9375   0.0044   0.3773

  Columns 31 through 40

    0.0370   0.4610   0.0371   0.1093   0.1093   0.1548   0.0627   0.0475   0.3095   0.0754

```
  Columns 41 through 50

    0.4206    0.4432    0.9642    0.0009    0.0009    0.0480    0.2456    0.1724    0.1104    0.6410

  Columns 51 through 60

    0.7565    0.2169    0.8613    0.0113    0.8336    0.2694    0.9948    0.4495    0.2881       NaN

>> ci

ci =

  Columns 1 through 10

   -0.1513    3.7857  -31.5319  -18.0977   -1.2626   -5.1665   -0.6249   -0.8130   -1.0563  -57.6354
    0.0790   18.0977   57.2958   -3.7857   -0.2652    2.8831    0.4027   -0.0759   -0.2215   -3.4757

  Columns 11 through 20

   -5.5835    0.0006 -228.0220   -7.4843   -0.6955   -7.0063   -0.6665   -7.2039   -0.5329   -0.8028
    4.2558    0.2995  146.1664    5.7343    0.4177    1.7257    0.4165    6.0122    0.4495    0.2750

  Columns 21 through 30

   -0.5951   -0.7357   -5.5361  -90.3163   -0.2600  -13.4807       NaN   -1.0909   -2.7852   -2.4307
    0.3173    1.2635    2.0472    2.3997    0.8434    3.0584       NaN    1.0075   -0.5287    6.3751

  Columns 31 through 40

    0.2965   -9.1886   -9.4108   -0.7901   -7.7480   -0.0218   -0.1216    0.0260   -1.1456  -12.2871
    9.4146    4.1886   -0.2948    7.7485    0.7897    0.1357    4.6383    4.6962    3.5873    0.6065

  Columns 41 through 50

   -1.4017   -3.5271   -1.0735    2.1971   -8.3239  -12.5078   -4.0245   -7.3151   -0.1661   -2.0366
    3.3378    1.5521    1.1235    8.3252   -2.1956   -0.0561   15.5967    1.3262    1.6022    3.2977

  Columns 51 through 60

   -0.9850   -0.9347   -3.3121    0.2795   -2.7851   -0.4067   -2.5128   -0.5336   -4.7004       NaN
    1.3517    4.0819    2.7732    2.1372    2.2490    1.4456    2.5295    1.1975    1.4087       NaN

>> stats.tstat

ans =

  Columns 1 through 10

   -0.6209    3.0227    0.5734   -3.0227   -3.0289   -0.5607   -0.4275   -2.3850   -3.0261   -2.2404
```

```
  Columns 11 through 20

   -0.2670    1.9922   -0.4334   -0.2617   -0.4933   -1.1989   -0.4563   -0.1784   -0.1677   -0.9681

  Columns 21 through 30

   -0.6019    0.5220   -0.9106   -1.8812    1.0460   -1.2457       NaN   -0.0786   -2.9097    0.8858

  Columns 31 through 40

    2.1067   -0.7394   -2.1060    1.6121   -1.6123    1.4312    1.8779    2.0012    1.0198   -1.7927

  Columns 41 through 50

    0.8078   -0.7691    0.0450    3.3997   -3.3987   -1.9952    1.1659   -1.3731    1.6084    0.4673

  Columns 51 through 60

    0.3108    1.2402   -0.1751    2.5818   -0.2105    1.1087    0.0065    0.7585   -1.0671       NaN

>> stats.df

ans =

  Columns 1 through 10

  120.0755  141.6154  141.1618  141.6154  137.1104  141.9412  141.4601  136.4577  140.4277   93.8414

  Columns 11 through 20

  127.6305   96.5533  112.5066  141.7035  141.9256  106.3335  141.8211  132.3436  141.6491  140.5049

  Columns 21 through 30

  141.6487  139.6185  123.6132  100.2931  128.0440  141.3784       NaN  130.3369  112.2214  136.1428

  Columns 31 through 40

  132.2714  130.4898  132.2809  131.1106  131.1044  129.8495  125.4029  124.7454  141.7133  126.7592

  Columns 41 through 50

  136.8954  133.8293  137.7952  119.5468  119.5437  137.6679  141.9812  112.5033  116.8749  141.9947

  Columns 51 through 60

  116.8592  141.5188  137.6793   96.9580  141.8824  141.5315  132.4506  134.1500  117.4942       NaN

>> stats.sd
```

```
ans =

  Columns 1 through 10

    0.2641   21.1458  139.8948   21.1458    1.6499   12.0910    1.5105    0.9991    1.3321   43.5799
    0.4169   22.2781  129.5000   22.2781    1.3629   12.3397    1.6068    1.2256    1.1979  107.2060

  Columns 11 through 20

   12.1600    0.5867  696.6963   20.5137    1.7086    8.5719    1.6725   17.1243    1.5275    1.7178
   17.2396    0.2532  395.7992   19.5959    1.6699   16.6047    1.6141   22.5897    1.4532    1.5488

  Columns 21 through 30

    1.4186    2.8284    9.0086   83.5513    1.9295   24.2524         0    2.6640    2.3791   11.8928
    1.3496    3.2255   13.5297  179.8130    1.3692   25.9175         0    3.6270    4.2056   14.6789

  Columns 31 through 40

   15.5917   17.0087   15.5873   14.6968   14.6958    0.2728    8.4263    8.2915    7.3423   22.6843
   11.8057   23.1027   11.8041   10.9248   10.9230    0.1989    5.7552    5.6102    7.0191   15.7987

  Columns 41 through 50

    6.4588    6.6850    3.6126   11.1164   11.1168   17.1342   29.6050    9.1401    3.2407    8.1202
    7.8538    8.6036    3.0281    6.9892    6.9892   20.4988   29.9481   16.0894    1.9617    8.0705

  Columns 51 through 60

    2.5919    7.8317   10.0170    3.6414    7.5288    2.8907    6.5407    2.9262    6.8214       NaN
    4.2827    7.3876    8.3749    1.5846    7.7488    2.7289    8.6133    2.2862   11.1695       NaN

>> hr

hr =

  Columns 1 through 18

     0     1     0     0     0     0     0     0     0     0     0     1     0     0     0     0     0     0

  Columns 19 through 36

     0     0     0     0     0     0     0     0   NaN     0     0     0     1     0     0     0     0     0

  Columns 37 through 54

     1     1     0     0     0     0     0     1     0     0     0     0     0     0     0     0     0     1

  Columns 55 through 60
```

```
     0     0     0     0     0   NaN

>> pr

pr =

  Columns 1 through 10

    0.7321    0.0015    0.2836    0.9985    0.9985    0.7121    0.6652    0.9908    0.9985    0.9863

  Columns 11 through 20

    0.6051    0.0246    0.6672    0.6030    0.6887    0.8834    0.6756    0.5706    0.5665    0.8327

  Columns 21 through 30

    0.7259    0.3013    0.8179    0.9686    0.1488    0.8925       NaN    0.5313    0.9978    0.1886

  Columns 31 through 40

    0.0185    0.7695    0.9815    0.0547    0.9453    0.0774    0.0314    0.0238    0.1548    0.9623

  Columns 41 through 50

    0.2103    0.7784    0.4821    0.0005    0.9995    0.9760    0.1228    0.9138    0.0552    0.3205

  Columns 51 through 60

    0.3783    0.1085    0.5694    0.0057    0.5832    0.1347    0.4974    0.2247    0.8559       NaN

>> cir

cir =

  Columns 1 through 10

   -0.1325    4.9483  -24.3159  -16.9351   -1.1815   -4.5126   -0.5414   -0.7531   -0.9885  -53.2123
       Inf       Inf       Inf       Inf       Inf       Inf       Inf       Inf       Inf       Inf

  Columns 11 through 20

   -4.7833    0.0250 -197.5430   -6.4105   -0.6051   -6.2945   -0.5785   -6.1295   -0.4531   -0.7152
       Inf       Inf       Inf       Inf       Inf       Inf       Inf       Inf       Inf       Inf

  Columns 21 through 30

   -0.5209   -0.5733   -4.9191  -82.7521   -0.1703  -12.1371       NaN   -0.9203   -2.6014   -1.7150
       Inf       Inf       Inf       Inf       Inf       Inf       Inf       Inf       Inf       Inf
```

```
  Columns 31 through 40

    1.0377   -8.1010   -8.6697   -0.0959   -7.0539   -0.0090    0.2656    0.4060   -0.7612  -11.2384
       Inf       Inf       Inf       Inf       Inf       Inf       Inf       Inf       Inf       Inf

  Columns 41 through 50

   -1.0165   -3.1143   -0.8950    2.6958   -7.8251  -11.4960   -2.4306   -6.6112   -0.0221   -1.6033
       Inf       Inf       Inf       Inf       Inf       Inf       Inf       Inf       Inf       Inf

  Columns 51 through 60

   -0.7948   -0.5272   -2.8176    0.4311   -2.3761   -0.2562   -2.1029   -0.3929   -4.2030       NaN
       Inf       Inf       Inf       Inf       Inf       Inf       Inf       Inf       Inf       Inf

>> statsr.tstat

ans =

  Columns 1 through 10

   -0.6209    3.0227    0.5734   -3.0227   -3.0289   -0.5607   -0.4275   -2.3850   -3.0261   -2.2404

  Columns 11 through 20

   -0.2670    1.9922   -0.4334   -0.2617   -0.4933   -1.1989   -0.4563   -0.1784   -0.1677   -0.9681

  Columns 21 through 30

   -0.6019    0.5220   -0.9106   -1.8812    1.0460   -1.2457       NaN   -0.0786   -2.9097    0.8858

  Columns 31 through 40

    2.1067   -0.7394   -2.1060    1.6121   -1.6123    1.4312    1.8779    2.0012    1.0198   -1.7927

  Columns 41 through 50

    0.8078   -0.7691    0.0450    3.3997   -3.3987   -1.9952    1.1659   -1.3731    1.6084    0.4673

  Columns 51 through 60

    0.3108    1.2402   -0.1751    2.5818   -0.2105    1.1087    0.0065    0.7585   -1.0671       NaN

>> statsr.df

ans =

  Columns 1 through 10

  120.0755  141.6154  141.1618  141.6154  137.1104  141.9412  141.4601  136.4577  140.4277   93.8414
```

```
  Columns 11 through 20

  127.6305   96.5533  112.5066  141.7035  141.9256  106.3335  141.8211  132.3436  141.6491  140.5049

  Columns 21 through 30

  141.6487  139.6185  123.6132  100.2931  128.0440  141.3784       NaN  130.3369  112.2214  136.1428

  Columns 31 through 40

  132.2714  130.4898  132.2809  131.1106  131.1044  129.8495  125.4029  124.7454  141.7133  126.7592

  Columns 41 through 50

  136.8954  133.8293  137.7952  119.5468  119.5437  137.6679  141.9812  112.5033  116.8749  141.9947

  Columns 51 through 60

  116.8592  141.5188  137.6793   96.9580  141.8824  141.5315  132.4506  134.1500  117.4942       NaN

>> statsr.sd

ans =

  Columns 1 through 10

    0.2641   21.1458  139.8948   21.1458    1.6499   12.0910    1.5105    0.9991    1.3321   43.5799
    0.4169   22.2781  129.5000   22.2781    1.3629   12.3397    1.6068    1.2256    1.1979  107.2060

  Columns 11 through 20

   12.1600    0.5867  696.6963   20.5137    1.7086    8.5719    1.6725   17.1243    1.5275    1.7178
   17.2396    0.2532  395.7992   19.5959    1.6699   16.6047    1.6141   22.5897    1.4532    1.5488

  Columns 21 through 30

    1.4186    2.8284    9.0086   83.5513    1.9295   24.2524         0    2.6640    2.3791   11.8928
    1.3496    3.2255   13.5297  179.8130    1.3692   25.9175         0    3.6270    4.2056   14.6789

  Columns 31 through 40

   15.5917   17.0087   15.5873   14.6968   14.6958    0.2728    8.4263    8.2915    7.3423   22.6843
   11.8057   23.1027   11.8041   10.9248   10.9230    0.1989    5.7552    5.6102    7.0191   15.7987

  Columns 41 through 50

    6.4588    6.6850    3.6126   11.1164   11.1168   17.1342   29.6050    9.1401    3.2407    8.1202
    7.8538    8.6036    3.0281    6.9892    6.9892   20.4988   29.9481   16.0894    1.9617    8.0705
```

```
  Columns 51 through 60

    2.5919    7.8317   10.0170    3.6414    7.5288    2.8907    6.5407    2.9262    6.8214       NaN
    4.2827    7.3876    8.3749    1.5846    7.7488    2.7289    8.6133    2.2862   11.1695       NaN

>> hl

hl =

  Columns 1 through 18

     0     0     0     1     1     0     0     1     1     1     0     0     0     0     0     0     0     0

  Columns 19 through 36

     0     0     0     0     0     1     0     0   NaN     0     1     0     0     0     1     0     0     0

  Columns 37 through 54

     0     0     0     1     0     0     0     0     1     1     0     0     0     0     0     0     0     0

  Columns 55 through 60

     0     0     0     0     0   NaN

pl =

  Columns 1 through 10

    0.2679    0.9985    0.7164    0.0015    0.0015    0.2879    0.3348    0.0092    0.0015    0.0137

  Columns 11 through 20

    0.3949    0.9754    0.3328    0.3970    0.3113    0.1166    0.3244    0.4294    0.4335    0.1673

  Columns 21 through 30

    0.2741    0.6987    0.1821    0.0314    0.8512    0.1075       NaN    0.4687    0.0022    0.8114

  Columns 31 through 40

    0.9815    0.2305    0.0185    0.9453    0.0547    0.9226    0.9686    0.9762    0.8452    0.0377

  Columns 41 through 50

    0.7897    0.2216    0.5179    0.9995    0.0005    0.0240    0.8772    0.0862    0.9448    0.6795

  Columns 51 through 60

    0.6217    0.8915    0.4306    0.9943    0.4168    0.8653    0.5026    0.7753    0.1441       NaN
```

```
>> cil

cil =

  Columns 1 through 10

     -Inf      -Inf      -Inf      -Inf      -Inf      -Inf      -Inf      -Inf      -Inf      -Inf
   0.0603   16.9351   50.0798   -4.9483   -0.3462    2.2293    0.3192   -0.1358   -0.2893   -7.8988

  Columns 11 through 20

     -Inf      -Inf      -Inf      -Inf      -Inf      -Inf      -Inf      -Inf      -Inf      -Inf
   3.4556    0.2751  115.6874    4.6605    0.3273    1.0139    0.3285    4.9378    0.3697    0.1874

  Columns 21 through 30

     -Inf      -Inf      -Inf      -Inf      -Inf      -Inf      -Inf      -Inf      -Inf      -Inf
   0.2432    1.1010    1.4302   -5.1645    0.7536    1.7149       NaN    0.8369   -0.7125    5.6595

  Columns 31 through 40

     -Inf      -Inf      -Inf      -Inf      -Inf      -Inf      -Inf      -Inf      -Inf      -Inf
   8.6734    3.1010   -1.0358    7.0543    0.0956    0.1229    4.2511    4.3163    3.2028   -0.4422

  Columns 41 through 50

     -Inf      -Inf      -Inf      -Inf      -Inf      -Inf      -Inf      -Inf      -Inf      -Inf
   2.9526    1.1393    0.9450    7.8264   -2.6944   -1.0679   14.0029    0.6224    1.4582    2.8644

  Columns 51 through 60

     -Inf      -Inf      -Inf      -Inf      -Inf      -Inf      -Inf      -Inf      -Inf      -Inf
   1.1615    3.6744    2.2787    1.9856    1.8400    1.2951    2.1196    1.0568    0.9114       NaN

>> statsl.tstat

ans =

  Columns 1 through 10

  -0.6209    3.0227    0.5734   -3.0227   -3.0289   -0.5607   -0.4275   -2.3850   -3.0261   -2.2404

  Columns 11 through 20

  -0.2670    1.9922   -0.4334   -0.2617   -0.4933   -1.1989   -0.4563   -0.1784   -0.1677   -0.9681

  Columns 21 through 30

  -0.6019    0.5220   -0.9106   -1.8812    1.0460   -1.2457       NaN   -0.0786   -2.9097    0.8858
```

```
  Columns 31 through 40

    2.1067   -0.7394   -2.1060    1.6121   -1.6123    1.4312    1.8779    2.0012    1.0198   -1.7927

  Columns 41 through 50

    0.8078   -0.7691    0.0450    3.3997   -3.3987   -1.9952    1.1659   -1.3731    1.6084    0.4673

  Columns 51 through 60

    0.3108    1.2402   -0.1751    2.5818   -0.2105    1.1087    0.0065    0.7585   -1.0671       NaN

>> statsl.df

ans =

  Columns 1 through 10

  120.0755  141.6154  141.1618  141.6154  137.1104  141.9412  141.4601  136.4577  140.4277   93.8414

  Columns 11 through 20

  127.6305   96.5533  112.5066  141.7035  141.9256  106.3335  141.8211  132.3436  141.6491  140.5049

  Columns 21 through 30

  141.6487  139.6185  123.6132  100.2931  128.0440  141.3784       NaN  130.3369  112.2214  136.1428

  Columns 31 through 40

  132.2714  130.4898  132.2809  131.1106  131.1044  129.8495  125.4029  124.7454  141.7133  126.7592

  Columns 41 through 50

  136.8954  133.8293  137.7952  119.5468  119.5437  137.6679  141.9812  112.5033  116.8749  141.9947

  Columns 51 through 60

  116.8592  141.5188  137.6793   96.9580  141.8824  141.5315  132.4506  134.1500  117.4942       NaN

>> statsl.sd

ans =

  Columns 1 through 10

    0.2641   21.1458  139.8948   21.1458    1.6499   12.0910    1.5105    0.9991    1.3321   43.5799
    0.4169   22.2781  129.5000   22.2781    1.3629   12.3397    1.6068    1.2256    1.1979  107.2060
```

Columns 11 through 20

```
 12.1600    0.5867  696.6963   20.5137    1.7086    8.5719    1.6725   17.1243    1.5275    1.7178
 17.2396    0.2532  395.7992   19.5959    1.6699   16.6047    1.6141   22.5897    1.4532    1.5488
```

Columns 21 through 30

```
  1.4186    2.8284    9.0086   83.5513    1.9295   24.2524         0    2.6640    2.3791   11.8928
  1.3496    3.2255   13.5297  179.8130    1.3692   25.9175         0    3.6270    4.2056   14.6789
```

Columns 31 through 40

```
 15.5917   17.0087   15.5873   14.6968   14.6958    0.2728    8.4263    8.2915    7.3423   22.6843
 11.8057   23.1027   11.8041   10.9248   10.9230    0.1989    5.7552    5.6102    7.0191   15.7987
```

Columns 41 through 50

```
  6.4588    6.6850    3.6126   11.1164   11.1168   17.1342   29.6050    9.1401    3.2407    8.1202
  7.8538    8.6036    3.0281    6.9892    6.9892   20.4988   29.9481   16.0894    1.9617    8.0705
```

Columns 51 through 60

```
  2.5919    7.8317   10.0170    3.6414    7.5288    2.8907    6.5407    2.9262    6.8214       NaN
  4.2827    7.3876    8.3749    1.5846    7.7488    2.7289    8.6133    2.2862   11.1695       NaN
```

d =

Columns 1 through 10

```
 -0.1035    0.5038    0.0956   -0.5038   -0.5048   -0.0935   -0.0713   -0.3975   -0.5044   -0.3734
```

Columns 11 through 20

```
 -0.0445    0.3320   -0.0722   -0.0436   -0.0822   -0.1998   -0.0761   -0.0297   -0.0279   -0.1614
```

Columns 21 through 30

```
 -0.1003    0.0870   -0.1518   -0.3135    0.1743   -0.2076       NaN   -0.0131   -0.4850    0.1476
```

Columns 31 through 40

```
  0.3511   -0.1232   -0.3510    0.2687   -0.2687    0.2385    0.3130    0.3335    0.1700   -0.2988
```

Columns 41 through 50

```
  0.1346   -0.1282    0.0075    0.5666   -0.5665   -0.3325    0.1943   -0.2289    0.2681    0.0779
```

Columns 51 through 59

```
  0.0518    0.2067   -0.0292    0.4303   -0.0351    0.1848    0.0011    0.1264   -0.1778
```

Professional Females vs. Professional Males

```
F =

  Columns 1 through 10

    0.8507   73.5507  270.3507   26.4493    6.6620   38.0310    2.3239    8.7183    6.3239  142.6338

  Columns 11 through 20

   29.3282    0.3810  317.6972   56.6761    3.3803   18.7873    3.4085   52.4873    2.0704    5.1408

  Columns 21 through 30

    3.5070    3.9718   28.8282  268.1690    2.4789   49.5930    1.0000    5.7887    5.6690   19.7197

  Columns 31 through 40

   26.4845   35.9606   73.5155   23.9338   76.0676    0.3324   12.9380   12.6437   10.9901   63.4254

  Columns 41 through 50

   47.7704   48.8577    3.3676   12.6915   87.2930   16.7324   68.9183   18.1606    3.5282    9.9127

  Columns 51 through 60

    4.2606   11.0366    8.3915    4.6394   15.0521    4.3028    7.7648    4.6352    8.3028       NaN

>> M

M =

  Columns 1 through 10

    1.1347   77.9000  312.2764   22.1000    6.4028   35.7250    1.8750    8.7361    6.0556  309.3194

  Columns 11 through 20

   38.6889    0.4223  345.1111   54.9583    3.2083   15.5625    3.3611   45.4028    1.9167    5.2917

  Columns 21 through 30

    3.3750    4.1389   27.2361  592.8056    2.7778   45.6583    1.0000    7.0000    9.6667   31.9958

  Columns 31 through 40

   31.5653   43.2986   68.4361   27.7278   72.2722    0.5264   16.6181   16.2708   11.1083   55.9972
```

```
  Columns 41 through 50

   48.5611   47.8597    3.5819   16.5014   83.5000   14.0208   66.4167   14.4500    4.3319   10.6194

  Columns 51 through 60

    5.5625   10.3306    9.3139    5.1542   10.8264    5.4222   11.7194    5.1958    7.0694       NaN

>>

>> h

h =

  Columns 1 through 18

     0     0     0     0     0     0     0     0     0     0     1     0     0     0     0     0     0     1

  Columns 19 through 36

     0     0     0     0     0     0     0     0   NaN     0     0     1     0     1     0     0     0     1

  Columns 37 through 54

     1     1     0     0     0     0     0     1     1     0     0     0     0     0     0     0     0     0

  Columns 55 through 60

     1     0     1     0     0   NaN

>> p

p =

  Columns 1 through 10

    0.1314    0.1509    0.0614    0.1509    0.3615    0.1868    0.0537    0.9282    0.2217    0.0933

  Columns 11 through 20

    0.0038    0.4736    0.7443    0.3838    0.3277    0.0650    0.7811    0.0031    0.3093    0.4231

  Columns 21 through 30

    0.3046    0.7774    0.4867    0.0907    0.2041    0.2845       NaN    0.0628    0.0721    0.0011

  Columns 31 through 40

    0.1121    0.0408    0.1122    0.2853    0.2851    0.0345    0.0415    0.0413    0.9641    0.1325
```

```
  Columns 41 through 50

    0.2287    0.1605    0.6789    0.0255    0.0263    0.2303    0.5973    0.1296    0.3713    0.6229

  Columns 51 through 60

    0.2867    0.6846    0.5548    0.6306    0.0144    0.2988    0.0071    0.6123    0.2916       NaN

>> ci

ci =

  Columns 1 through 10

   -0.6543  -10.3042  -85.8892   -1.6056   -0.3007   -1.1307   -0.0072   -0.4078   -0.1639 -362.0123
    0.0863    1.6056    2.0378   10.3042    0.8190    5.7427    0.9051    0.3722    0.7007   28.6411

  Columns 11 through 20

  -15.6503   -0.1551 -193.3072   -2.1693   -0.1742   -0.2025   -0.2889    2.4304   -0.1442   -0.5220
   -3.0711    0.0725  138.4794    5.6048    0.5181    6.6522    0.3836   11.7387    0.4517    0.2203

  Columns 21 through 30

   -0.1213   -1.3331   -2.9213 -701.9777   -0.7620   -3.3065       NaN   -2.4884   -8.3627  -19.5525
    0.3854    0.9990    6.1054   52.7046    0.1642   11.1758       NaN    0.0658    0.3674   -4.9998

  Columns 31 through 40

  -11.3656  -14.3651   -1.2057  -10.7880   -3.1984   -0.3736   -7.2159   -7.1094   -5.3054   -2.2768
    1.2040   -0.3110   11.3645    3.2000   10.7892   -0.0144   -0.1442   -0.1449    5.0691   17.1331

  Columns 41 through 50

   -2.0842   -0.4008   -1.2362   -7.1462    0.4546   -1.7376   -6.8383   -1.1017   -2.5753   -3.5441
    0.5028    2.3968    0.8075   -0.4734    7.1313    7.1607   11.8416    8.5228    0.9678    2.1305

  Columns 51 through 60

   -3.7087   -2.7298   -4.0036   -2.6279    0.8562   -3.2414   -6.8152   -2.7425   -1.0708       NaN
    1.1048    4.1419    2.1590    1.5984    7.5953    1.0026   -1.0941    1.6212    3.5375       NaN

>> stats.tstat

ans =

  Columns 1 through 10

   -1.5199   -1.4444   -1.8854    1.4444    0.9156    1.3265    1.9498   -0.0903    1.2275   -1.6987
```

```
  Columns 11 through 20

   -2.9430   -0.7189   -0.3268    0.8736    0.9822    1.8601    0.2784    3.0116    1.0205   -0.8034

  Columns 21 through 30

    1.0305   -0.2832    0.6974   -1.7125   -1.2760    1.0743       NaN   -1.8782   -1.8237   -3.3357

  Columns 31 through 40

   -1.6003   -2.0645    1.5997   -1.0728    1.0732   -2.1372   -2.0584   -2.0601   -0.0451    1.5135

  Columns 41 through 50

   -1.2094    1.4111   -0.4149   -2.2576    2.2463    1.2050    0.5295    1.5247   -0.8970   -0.4929

  Columns 51 through 60

   -1.0695    0.4072   -0.5922   -0.4821    2.4843   -1.0429   -2.7343   -0.5080    1.0590       NaN

>> stats.df

ans =

  Columns 1 through 10

  110.3845  135.4537  140.0271  135.4537  135.0384  140.8516  113.5708  140.1284  139.9249   78.8017

  Columns 11 through 20

  136.9090  119.3378  136.6449  140.9266  140.7967  140.9920  140.9126  129.2597  136.1619  139.9377

  Columns 21 through 30

  138.7141  140.6736  139.6661   78.7401  139.0056  139.4144       NaN  117.9930   76.8526  139.4004

  Columns 31 through 40

  122.7570  140.7176  122.7682  135.8607  135.8634  129.3847  134.6406  134.3699  126.3178  137.5658

  Columns 41 through 50

  129.5277  134.9112  133.2290  139.9241  139.9562  139.0147  140.2514  137.7375  140.9911  127.7059

  Columns 51 through 60

  139.6463  110.9147  130.5596  123.9684  114.1591  140.9999  134.3586  140.9865  130.2327       NaN

>> stats.sd
```

```
ans =

   1.0e+03 *

  Columns 1 through 10

    0.0014    0.0196    0.1264    0.0196    0.0015    0.0105    0.0017    0.0011    0.0012    0.1890
    0.0008    0.0162    0.1393    0.0162    0.0019    0.0103    0.0010    0.0012    0.0014    0.8106

  Columns 11 through 20

    0.0172    0.0003    0.4511    0.0115    0.0010    0.0103    0.0010    0.0159    0.0010    0.0012
    0.0207    0.0004    0.5481    0.0120    0.0011    0.0105    0.0010    0.0119    0.0008    0.0011

  Columns 21 through 30

    0.0008    0.0036    0.0129    0.3637    0.0015    0.0229         0    0.0029    0.0037    0.0206
    0.0007    0.0035    0.0144    1.5663    0.0013    0.0209         0    0.0047    0.0182    0.0233

  Columns 31 through 40

    0.0222    0.0206    0.0222    0.0230    0.0230    0.0005    0.0117    0.0116    0.0180    0.0314
    0.0150    0.0219    0.0150    0.0191    0.0191    0.0006    0.0095    0.0094    0.0128    0.0271

  Columns 41 through 50

    0.0044    0.0046    0.0027    0.0096    0.0096    0.0141    0.0291    0.0133    0.0053    0.0070
    0.0033    0.0038    0.0035    0.0106    0.0106    0.0127    0.0274    0.0157    0.0054    0.0099

  Columns 51 through 60

    0.0069    0.0127    0.0078    0.0074    0.0123    0.0064    0.0076    0.0066    0.0079       NaN
    0.0077    0.0073    0.0106    0.0051    0.0074    0.0065    0.0096    0.0066    0.0059       NaN

>> hr

hr =

  Columns 1 through 18

     0     0     0     0     0     0     1     0     0     0     0     0     0     0     0     1     0     1

  Columns 19 through 36

     0     0     0     0     0     0     0     0   NaN     0     0     0     0     0     0     0     0     0

  Columns 37 through 54

     0     0     0     0     0     0     0     0     1     0     0     0     0     0     0     0     0     0
```

```
  Columns 55 through 60

     1     0     0     0     0   NaN

>> pr

pr =

  Columns 1 through 10

    0.9343    0.9245    0.9693    0.0755    0.1808    0.0934    0.0268    0.5359    0.1109    0.9533

  Columns 11 through 20

    0.9981    0.7632    0.6278    0.1919    0.1639    0.0325    0.3906    0.0016    0.1547    0.7884

  Columns 21 through 30

    0.1523    0.6113    0.2434    0.9546    0.8980    0.1423       NaN    0.9686    0.9640    0.9995

  Columns 31 through 40

    0.9439    0.9796    0.0561    0.8574    0.1425    0.9828    0.9793    0.9793    0.5179    0.0662

  Columns 41 through 50

    0.8856    0.0803    0.6606    0.9872    0.0131    0.1151    0.2986    0.0648    0.8144    0.6885

  Columns 51 through 60

    0.8567    0.3423    0.7226    0.6847    0.0072    0.8506    0.9965    0.6939    0.1458       NaN

>> pir

ans =

            Number of Ctxs : 0
>> cir

cir =

  Columns 1 through 10

   -0.5940   -9.3363  -78.7457   -0.6377   -0.2097   -0.5724    0.0671   -0.3444   -0.0937 -330.0121
       Inf       Inf       Inf       Inf       Inf       Inf       Inf       Inf       Inf       Inf

  Columns 11 through 20

  -14.6281   -0.1366 -166.3449   -1.5378   -0.1179    0.3543   -0.2342    3.1873   -0.0958   -0.4617
```

```
     Inf       Inf       Inf       Inf       Inf       Inf       Inf       Inf       Inf       Inf

  Columns 21 through 30

   -0.0801   -1.1436   -2.1879 -640.1572   -0.6868   -2.1299       NaN   -2.2804   -7.6473  -18.3701
       Inf       Inf       Inf       Inf       Inf       Inf       Inf       Inf       Inf       Inf

  Columns 31 through 40

  -10.3428  -13.2234   -0.1830   -9.6512   -2.0616   -0.3444   -6.6411   -6.5434   -4.4616   -0.6996
       Inf       Inf       Inf       Inf       Inf       Inf       Inf       Inf       Inf       Inf

  Columns 41 through 50

   -1.8738   -0.1734   -1.0701   -6.6041    0.9971   -1.0146   -5.3207   -0.3196   -2.2875   -3.0826
       Inf       Inf       Inf       Inf       Inf       Inf       Inf       Inf       Inf       Inf

  Columns 51 through 60

   -3.3176   -2.1699   -3.5026   -2.2840    1.4050   -2.8966   -6.3502   -2.3880   -0.6961       NaN
       Inf       Inf       Inf       Inf       Inf       Inf       Inf       Inf       Inf       Inf

>> statsr.tstat

ans =

  Columns 1 through 10

   -1.5199   -1.4444   -1.8854    1.4444    0.9156    1.3265    1.9498   -0.0903    1.2275   -1.6987

  Columns 11 through 20

   -2.9430   -0.7189   -0.3268    0.8736    0.9822    1.8601    0.2784    3.0116    1.0205   -0.8034

  Columns 21 through 30

    1.0305   -0.2832    0.6974   -1.7125   -1.2760    1.0743       NaN   -1.8782   -1.8237   -3.3357

  Columns 31 through 40

   -1.6003   -2.0645    1.5997   -1.0728    1.0732   -2.1372   -2.0584   -2.0601   -0.0451    1.5135

  Columns 41 through 50

   -1.2094    1.4111   -0.4149   -2.2576    2.2463    1.2050    0.5295    1.5247   -0.8970   -0.4929

  Columns 51 through 60

   -1.0695    0.4072   -0.5922   -0.4821    2.4843   -1.0429   -2.7343   -0.5080    1.0590       NaN
```

```
>> statsr.df

ans =

  Columns 1 through 10

  110.3845  135.4537  140.0271  135.4537  135.0384  140.8516  113.5708  140.1284  139.9249   78.8017

  Columns 11 through 20

  136.9090  119.3378  136.6449  140.9266  140.7967  140.9920  140.9126  129.2597  136.1619  139.9377

  Columns 21 through 30

  138.7141  140.6736  139.6661   78.7401  139.0056  139.4144       NaN  117.9930   76.8526  139.4004

  Columns 31 through 40

  122.7570  140.7176  122.7682  135.8607  135.8634  129.3847  134.6406  134.3699  126.3178  137.5658

  Columns 41 through 50

  129.5277  134.9112  133.2290  139.9241  139.9562  139.0147  140.2514  137.7375  140.9911  127.7059

  Columns 51 through 60

  139.6463  110.9147  130.5596  123.9684  114.1591  140.9999  134.3586  140.9865  130.2327       NaN

>> statsr.sd

ans =

   1.0e+03 *

  Columns 1 through 10

    0.0014    0.0196    0.1264    0.0196    0.0015    0.0105    0.0017    0.0011    0.0012    0.1890
    0.0008    0.0162    0.1393    0.0162    0.0019    0.0103    0.0010    0.0012    0.0014    0.8106

  Columns 11 through 20

    0.0172    0.0003    0.4511    0.0115    0.0010    0.0103    0.0010    0.0159    0.0010    0.0012
    0.0207    0.0004    0.5481    0.0120    0.0011    0.0105    0.0010    0.0119    0.0008    0.0011

  Columns 21 through 30

    0.0008    0.0036    0.0129    0.3637    0.0015    0.0229         0    0.0029    0.0037    0.0206
    0.0007    0.0035    0.0144    1.5663    0.0013    0.0209         0    0.0047    0.0182    0.0233

  Columns 31 through 40
```

```
    0.0222    0.0206    0.0222    0.0230    0.0230    0.0005    0.0117    0.0116    0.0180    0.0314
    0.0150    0.0219    0.0150    0.0191    0.0191    0.0006    0.0095    0.0094    0.0128    0.0271

  Columns 41 through 50

    0.0044    0.0046    0.0027    0.0096    0.0096    0.0141    0.0291    0.0133    0.0053    0.0070
    0.0033    0.0038    0.0035    0.0106    0.0106    0.0127    0.0274    0.0157    0.0054    0.0099

  Columns 51 through 60

    0.0069    0.0127    0.0078    0.0074    0.0123    0.0064    0.0076    0.0066    0.0079       NaN
    0.0077    0.0073    0.0106    0.0051    0.0074    0.0065    0.0096    0.0066    0.0059       NaN

>> hl

hl =

  Columns 1 through 18

     0     0     1     0     0     0     0     0     0     1     1     0     0     0     0     0     0     0

  Columns 19 through 36

     0     0     0     0     0     1     0     0   NaN     1     1     1     0     1     0     0     0     1

  Columns 37 through 54

     1     1     0     0     0     0     0     1     0     0     0     0     0     0     0     0     0     0

  Columns 55 through 60

     0     0     1     0     0   NaN

>> pl

pl =

  Columns 1 through 10

    0.0657    0.0755    0.0307    0.9245    0.8192    0.9066    0.9732    0.4641    0.8891    0.0467

  Columns 11 through 20

    0.0019    0.2368    0.3722    0.8081    0.8361    0.9675    0.6094    0.9984    0.8453    0.2116

  Columns 21 through 30

    0.8477    0.3887    0.7566    0.0454    0.1020    0.8577       NaN    0.0314    0.0360    0.0005
```

```
  Columns 31 through 40

    0.0561    0.0204    0.9439    0.1426    0.8575    0.0172    0.0207    0.0207    0.4821    0.9338

  Columns 41 through 50

    0.1144    0.9197    0.3394    0.0128    0.9869    0.8849    0.7014    0.9352    0.1856    0.3115

  Columns 51 through 60

    0.1433    0.6577    0.2774    0.3153    0.9928    0.1494    0.0035    0.3061    0.8542       NaN

>> cil

cil =

  Columns 1 through 10

      -Inf      -Inf      -Inf      -Inf      -Inf      -Inf      -Inf      -Inf      -Inf      -Inf
    0.0260    0.6377   -5.1056    9.3363    0.7280    5.1843    0.8308    0.3088    0.6304   -3.3592

  Columns 11 through 20

      -Inf      -Inf      -Inf      -Inf      -Inf      -Inf      -Inf      -Inf      -Inf      -Inf
   -4.0934    0.0539  111.5170    4.9732    0.4618    6.0953    0.3289   10.9818    0.4033    0.1600

  Columns 21 through 30

      -Inf      -Inf      -Inf      -Inf      -Inf      -Inf      -Inf      -Inf      -Inf      -Inf
    0.3442    0.8095    5.3720   -9.1159    0.0890    9.9991       NaN   -0.1421   -0.3480   -6.1821

  Columns 31 through 40

      -Inf      -Inf      -Inf      -Inf      -Inf      -Inf      -Inf      -Inf      -Inf      -Inf
    0.1813   -1.4527   10.3417    2.0632    9.6524   -0.0436   -0.7189   -0.7110    4.2252   15.5559

  Columns 41 through 50

      -Inf      -Inf      -Inf      -Inf      -Inf      -Inf      -Inf      -Inf      -Inf      -Inf
    0.2925    2.1694    0.6414   -1.0156    6.5888    6.4377   10.3240    7.7407    0.6800    1.6690

  Columns 51 through 60

      -Inf      -Inf      -Inf      -Inf      -Inf      -Inf      -Inf      -Inf      -Inf      -Inf
    0.7137    3.5821    1.6579    1.2546    7.0464    0.6578   -1.5591    1.2667    3.1628       NaN

>> statsl.tstat

ans =
```

```
  Columns 1 through 10

   -1.5199   -1.4444   -1.8854    1.4444    0.9156    1.3265    1.9498   -0.0903    1.2275   -1.6987

  Columns 11 through 20

   -2.9430   -0.7189   -0.3268    0.8736    0.9822    1.8601    0.2784    3.0116    1.0205   -0.8034

  Columns 21 through 30

    1.0305   -0.2832    0.6974   -1.7125   -1.2760    1.0743       NaN   -1.8782   -1.8237   -3.3357

  Columns 31 through 40

   -1.6003   -2.0645    1.5997   -1.0728    1.0732   -2.1372   -2.0584   -2.0601   -0.0451    1.5135

  Columns 41 through 50

   -1.2094    1.4111   -0.4149   -2.2576    2.2463    1.2050    0.5295    1.5247   -0.8970   -0.4929

  Columns 51 through 60

   -1.0695    0.4072   -0.5922   -0.4821    2.4843   -1.0429   -2.7343   -0.5080    1.0590       NaN

>> statsl.df

ans =

  Columns 1 through 10

  110.3845  135.4537  140.0271  135.4537  135.0384  140.8516  113.5708  140.1284  139.9249   78.8017

  Columns 11 through 20

  136.9090  119.3378  136.6449  140.9266  140.7967  140.9920  140.9126  129.2597  136.1619  139.9377

  Columns 21 through 30

  138.7141  140.6736  139.6661   78.7401  139.0056  139.4144       NaN  117.9930   76.8526  139.4004

  Columns 31 through 40

  122.7570  140.7176  122.7682  135.8607  135.8634  129.3847  134.6406  134.3699  126.3178  137.5658

  Columns 41 through 50

  129.5277  134.9112  133.2290  139.9241  139.9562  139.0147  140.2514  137.7375  140.9911  127.7059

  Columns 51 through 60
```

```
  139.6463  110.9147  130.5596  123.9684  114.1591  140.9999  134.3586  140.9865  130.2327       NaN

>> statsl.sd

ans =

   1.0e+03 *

  Columns 1 through 10

    0.0014    0.0196    0.1264    0.0196    0.0015    0.0105    0.0017    0.0011    0.0012    0.1890
    0.0008    0.0162    0.1393    0.0162    0.0019    0.0103    0.0010    0.0012    0.0014    0.8106

  Columns 11 through 20

    0.0172    0.0003    0.4511    0.0115    0.0010    0.0103    0.0010    0.0159    0.0010    0.0012
    0.0207    0.0004    0.5481    0.0120    0.0011    0.0105    0.0010    0.0119    0.0008    0.0011

  Columns 21 through 30

    0.0008    0.0036    0.0129    0.3637    0.0015    0.0229         0    0.0029    0.0037    0.0206
    0.0007    0.0035    0.0144    1.5663    0.0013    0.0209         0    0.0047    0.0182    0.0233

  Columns 31 through 40

    0.0222    0.0206    0.0222    0.0230    0.0230    0.0005    0.0117    0.0116    0.0180    0.0314
    0.0150    0.0219    0.0150    0.0191    0.0191    0.0006    0.0095    0.0094    0.0128    0.0271

  Columns 41 through 50

    0.0044    0.0046    0.0027    0.0096    0.0096    0.0141    0.0291    0.0133    0.0053    0.0070
    0.0033    0.0038    0.0035    0.0106    0.0106    0.0127    0.0274    0.0157    0.0054    0.0099

  Columns 51 through 60

    0.0069    0.0127    0.0078    0.0074    0.0123    0.0064    0.0076    0.0066    0.0079       NaN
    0.0077    0.0073    0.0106    0.0051    0.0074    0.0065    0.0096    0.0066    0.0059       NaN
```

```
>> d

d =

  Columns 1 through 10

   -0.2551   -0.2419   -0.3151    0.2419    0.1529    0.2219    0.3272   -0.0151    0.2051   -0.2823

  Columns 11 through 20

   -0.4916   -0.1199   -0.0546    0.1461    0.1642    0.3111    0.0466    0.5047    0.1709   -0.1344

  Columns 21 through 30

    0.1725   -0.0474    0.1166   -0.2846   -0.2136    0.1798       NaN   -0.3131   -0.3030   -0.5574

  Columns 31 through 40

   -0.2683   -0.3451    0.2683   -0.1797    0.1797   -0.3567   -0.3448   -0.3451   -0.0076    0.2534

  Columns 41 through 50

   -0.2027    0.2363   -0.0693   -0.3773    0.3754    0.2017    0.0886    0.2547   -0.1500   -0.0822

  Columns 51 through 59

   -0.1787    0.0684   -0.0988   -0.0808    0.4169   -0.1744   -0.4566   -0.0850    0.1775

>>
```

Young Laymen vs. Old Laymen

```
>> Y

Y =

  Columns 1 through 10

    0.4319   63.0000  175.7694   37.0000    6.2917   43.1986    2.4444    7.8472    5.9583   64.3750

  Columns 11 through 20

   21.2000    0.3422  271.7333   61.0694    3.6944   18.9375    3.5833   57.8042    2.4167    5.0833

  Columns 21 through 30

    3.6667    4.2083   24.3819  105.1667    1.6250   68.6833    1.0000    4.0556    3.4958    4.3014

  Columns 31 through 40

    4.7736   32.8389   95.2278    3.0264   96.9736    0.0444    1.2736    1.1375    1.7528   95.8472

  Columns 41 through 50

   46.5347   50.0472    3.4181    6.3222   93.6792   18.7181   53.4694   14.9764    0.3861   14.1028

  Columns 51 through 60

    0.4667   14.8208   14.0278    0.2778   13.0097    0.5486   13.7944    0.6014   12.9833       NaN

>> O

O =

  Columns 1 through 10

    0.4264   59.2444  215.2486   40.7556    5.8611   39.4931    2.6389    7.8472    5.6528   71.0139

  Columns 11 through 20

   22.9722    0.4316  442.0889   62.3056    3.8333   22.2944    3.8194   62.0361    2.4861    5.3472

  Columns 21 through 30

    3.8889    4.1944   26.5931  123.4861    1.8889   61.4139    1.0000    4.9028    4.4556    8.5181

  Columns 31 through 40
```

```
   11.7847   32.9278   88.2194    9.7667   90.2361    0.1542    5.6569    5.5264    4.1097   84.7042

  Columns 41 through 50

   45.2889   51.6375    3.0764    8.5611   91.4389   24.1750   59.7583   17.0097    1.7542   12.1778

  Columns 51 through 60

    1.8639   13.2194   13.0889    1.9278   12.7917    1.5708   11.0833    1.4639   12.0292       NaN

>> h

h =

  Columns 1 through 18

     0     0     0     0     0     0     0     0     0     0     0     0     0     0     0     0     0     0

  Columns 19 through 36

     0     0     0     0     0     0     0     0   NaN     0     0     0     1     0     1     1     1     1

  Columns 37 through 54

     1     1     1     1     0     0     0     0     0     0     0     0     1     0     1     0     0     1

  Columns 55 through 60

     0     1     1     1     0   NaN

>> p

p =

  Columns 1 through 10

    0.9242    0.3146    0.0785    0.3146    0.0972    0.0680    0.4551    1.0000    0.1601    0.6329

  Columns 11 through 20

    0.4765    0.2427    0.0717    0.7121    0.6226    0.1294    0.3893    0.2049    0.7802    0.3346

  Columns 21 through 30

    0.3363    0.9781    0.2503    0.4390    0.3462    0.0828       NaN    0.1093    0.1011    0.0578

  Columns 31 through 40

    0.0025    0.9791    0.0025    0.0018    0.0018    0.0057    0.0003    0.0002    0.0487    0.0006
```

```
  Columns 41 through 50

    0.2997    0.2163    0.5390    0.1635    0.1633    0.0865    0.2068    0.3549    0.0023    0.1532

  Columns 51 through 60

    0.0178    0.2089    0.5424    0.0006    0.8643    0.0288    0.0323    0.0483    0.5383       NaN

>> ci

ci =

  Columns 1 through 10

   -0.1100   -3.6006  -83.5242  -11.1117   -0.0794   -0.2779   -0.7077   -0.3758   -0.1223  -34.0571
    0.1211   11.1117    4.5659    3.6006    0.9405    7.6890    0.3188    0.3758    0.7334   20.7793

  Columns 11 through 20

   -6.6799   -0.2404 -356.0989   -7.8440   -0.6955   -7.7120   -0.7766  -10.8017   -0.5605   -0.8027
    3.1354    0.0617   15.3878    5.3718    0.4178    0.9981    0.3044    2.3378    0.4217    0.2750

  Columns 21 through 30

   -0.6776   -0.9865   -6.0053  -64.9852   -0.8163   -0.9571       NaN   -1.8868   -2.1099   -8.5752
    0.2332    1.0143    1.5831   28.3464    0.2885   15.4960       NaN    0.1924    0.1904    0.1419

  Columns 31 through 40

  -11.4981   -6.7877    2.5224  -10.9067    2.5714   -0.1869   -6.6904   -6.6527   -4.7002    4.8761
   -2.5242    6.6100   11.4943   -2.5739   10.9036   -0.0325   -2.0762   -2.1251   -0.0137   17.4100

  Columns 41 through 50

   -1.1213   -4.1231   -0.7555   -5.3996   -0.9204  -11.7065  -16.0916   -6.3707   -2.2340   -0.7253
    3.6129    0.9425    1.4388    0.9218    5.4009    0.7926    3.5138    2.3041   -0.5022    4.5753

  Columns 51 through 60

   -2.5465   -0.9065   -2.1031   -2.5624   -2.2992   -1.9372    0.2318   -1.7184   -2.1042       NaN
   -0.2479    4.1092    3.9808   -0.7376    2.7353   -0.1072    5.1904   -0.0066    4.0126       NaN

>> stats.tstat

ans =

  Columns 1 through 10

    0.0954    1.0092   -1.7746   -1.0092    1.6708    1.8395   -0.7491         0    1.4126   -0.4788
```

```
  Columns 11 through 20

   -0.7139   -1.1764   -1.8238   -0.3698   -0.4933   -1.5291   -0.8636   -1.2739   -0.2795   -0.9681

  Columns 21 through 30

   -0.9650    0.0274   -1.1563   -0.7760   -0.9458    1.7469       NaN   -1.6120   -1.6529   -1.9129

  Columns 31 through 40

   -3.0949   -0.0262    3.0944   -3.2050    3.2039   -2.8132   -3.7771   -3.8559   -1.9889    3.5274

  Columns 41 through 50

    1.0412   -1.2426    0.6158   -1.4008    1.4017   -1.7272   -1.2683   -0.9291   -3.1405    1.4359

  Columns 51 through 60

   -2.4157    1.2624    0.6109   -3.5993    0.1712   -2.2096    2.1618   -1.9942    0.6170       NaN

>> stats.df

ans =

  Columns 1 through 10

   96.5392  141.9222  120.6220  141.9222  126.7546  136.6986  137.1397  138.3477  132.1104  137.6065

  Columns 11 through 20

  140.8273   86.0743   84.1477  141.1442  140.8087  100.9322  140.6920  136.0255  141.7779  141.6388

  Columns 21 through 30

  136.7940  141.2808   99.1256  141.9121  120.4726  141.6542       NaN  132.3857  114.3362  138.5026

  Columns 31 through 40

  115.8660  136.1832  115.8684  113.4237  113.4309  120.3204   85.7753   83.2255  138.1121  100.5406

  Columns 41 through 50

  130.8821  125.2382  135.7337  135.7801  135.7802  132.1223  140.9682  109.4827   86.5195  140.5598

  Columns 51 through 60

   88.5999  141.2084  124.0862   79.0405  141.3494  134.3209  141.2108  126.0191  135.6402       NaN

>> stats.sd
```

```
ans =

  Columns 1 through 10

    0.4537   22.0643  101.5699   22.0643    1.2496   10.8311    1.4031    1.0436    1.1062   90.3244
    0.1957   22.5871  159.1171   22.5871    1.7944   13.2230    1.6976    1.2294    1.4647   75.3982

  Columns 11 through 20

   14.1988    0.2008  231.6426   20.8214    1.7654    7.9266    1.7178   17.7214    1.4608    1.6763
   15.5594    0.6127  757.9703   19.2585    1.6098   16.8577    1.5594   21.9223    1.5198    1.5936

  Columns 21 through 30

    1.5105    2.9260    6.7129  143.3904    1.2720   24.3441         0    2.6953    2.4833   12.1299
    1.2397    3.1428   14.7719  139.8654    1.9969   25.5780         0    3.5531    4.2551   14.2384

  Columns 31 through 40

    9.8493   18.1031    9.8473    8.9053    8.9053    0.1775    3.0382    2.7287    6.4866   11.3383
   16.5075   22.3266   16.5035   15.4643   15.4627    0.2793    9.3669    9.2648    7.6838   24.2890

  Columns 41 through 50

    6.0433    6.1148    3.6690    8.5015    8.5015   16.1583   28.4501    8.8576    1.1666    7.6255
    8.1588    8.9740    2.9496   10.5660   10.5660   21.3916   30.9977   16.3218    3.5074    8.4407

  Columns 51 through 60

    1.6412    7.3209    7.2612    0.9018    7.3762    2.4213    7.2380    2.0823    8.2132       NaN
    4.6252    7.8912   10.8325    3.7838    7.8949    3.0899    7.8009    3.0219   10.2345       NaN

>> hr

hr =

  Columns 1 through 18

     0     0     0     0     1     1     0     0     0     0     0     0     0     0     0     0     0     0

  Columns 19 through 36

     0     0     0     0     0     0     0     1   NaN     0     0     0     0     0     1     0     1     0

  Columns 37 through 54

     0     0     0     1     0     0     0     0     0     0     0     0     0     0     0     0     0     0

  Columns 55 through 60
```

```
     0     0     1     0     0   NaN

>> pr

pr =

  Columns 1 through 10

    0.4621    0.1573    0.9608    0.8427    0.0486    0.0340    0.7725    0.5000    0.0801    0.6836

  Columns 11 through 20

    0.7618    0.8787    0.9641    0.6440    0.6887    0.9353    0.8054    0.8976    0.6099    0.8327

  Columns 21 through 30

    0.8319    0.4891    0.8748    0.7805    0.8269    0.0414       NaN    0.9453    0.9495    0.9711

  Columns 31 through 40

    0.9988    0.5104    0.0012    0.9991    0.0009    0.9971    0.9999    0.9999    0.9757    0.0003

  Columns 41 through 50

    0.1499    0.8918    0.2695    0.9182    0.0816    0.9568    0.8966    0.8226    0.9988    0.0766

  Columns 51 through 60

    0.9911    0.1045    0.2712    0.9997    0.4321    0.9856    0.0162    0.9759    0.2691       NaN

>> cir

cir =

  Columns 1 through 10

   -0.0912   -2.4055  -76.3553   -9.9166    0.0036    0.3696   -0.6243   -0.3147   -0.0528  -29.6012
       Inf       Inf       Inf       Inf       Inf       Inf       Inf       Inf       Inf       Inf

  Columns 11 through 20

   -5.8825   -0.2157 -325.7051   -6.7704   -0.6051   -7.0015   -0.6888   -9.7338   -0.4808   -0.7152
       Inf       Inf       Inf       Inf       Inf       Inf       Inf       Inf       Inf       Inf

  Columns 21 through 30

   -0.6036   -0.8240   -5.3861  -57.4038   -0.7264    0.3794       NaN   -1.7178   -1.9226   -7.8669
       Inf       Inf       Inf       Inf       Inf       Inf       Inf       Inf       Inf       Inf

  Columns 31 through 40
```

```
  -10.7674   -5.6990    3.2529  -10.2280    3.2500   -0.1744   -6.3131   -6.2822   -4.3194    5.8986
       Inf       Inf       Inf       Inf       Inf       Inf       Inf       Inf       Inf       Inf

  Columns 41 through 50

   -0.7364   -3.7110   -0.5772   -4.8858   -0.4067  -10.6904  -14.4989   -5.6639   -2.0923   -0.2947
       Inf       Inf       Inf       Inf       Inf       Inf       Inf       Inf       Inf       Inf

  Columns 51 through 60

   -2.3586   -0.4990   -1.6081   -2.4130   -1.8902   -1.7885    0.6347   -1.5792   -1.6071       NaN
       Inf       Inf       Inf       Inf       Inf       Inf       Inf       Inf       Inf       Inf

>> statsr.tstat

ans =

  Columns 1 through 10

    0.0954    1.0092   -1.7746   -1.0092    1.6708    1.8395   -0.7491         0    1.4126   -0.4788

  Columns 11 through 20

   -0.7139   -1.1764   -1.8238   -0.3698   -0.4933   -1.5291   -0.8636   -1.2739   -0.2795   -0.9681

  Columns 21 through 30

   -0.9650    0.0274   -1.1563   -0.7760   -0.9458    1.7469       NaN   -1.6120   -1.6529   -1.9129

  Columns 31 through 40

   -3.0949   -0.0262    3.0944   -3.2050    3.2039   -2.8132   -3.7771   -3.8559   -1.9889    3.5274

  Columns 41 through 50

    1.0412   -1.2426    0.6158   -1.4008    1.4017   -1.7272   -1.2683   -0.9291   -3.1405    1.4359

  Columns 51 through 60

   -2.4157    1.2624    0.6109   -3.5993    0.1712   -2.2096    2.1618   -1.9942    0.6170       NaN

>> statsr.df

ans =

  Columns 1 through 10

   96.5392  141.9222  120.6220  141.9222  126.7546  136.6986  137.1397  138.3477  132.1104  137.6065
```

```
  Columns 11 through 20

  140.8273   86.0743   84.1477  141.1442  140.8087  100.9322  140.6920  136.0255  141.7779  141.6388

  Columns 21 through 30

  136.7940  141.2808   99.1256  141.9121  120.4726  141.6542       NaN  132.3857  114.3362  138.5026

  Columns 31 through 40

  115.8660  136.1832  115.8684  113.4237  113.4309  120.3204   85.7753   83.2255  138.1121  100.5406

  Columns 41 through 50

  130.8821  125.2382  135.7337  135.7801  135.7802  132.1223  140.9682  109.4827   86.5195  140.5598

  Columns 51 through 60

   88.5999  141.2084  124.0862   79.0405  141.3494  134.3209  141.2108  126.0191  135.6402       NaN

>> statsr.sd

ans =

  Columns 1 through 10

    0.4537   22.0643  101.5699   22.0643    1.2496   10.8311    1.4031    1.0436    1.1062   90.3244
    0.1957   22.5871  159.1171   22.5871    1.7944   13.2230    1.6976    1.2294    1.4647   75.3982

  Columns 11 through 20

   14.1988    0.2008  231.6426   20.8214    1.7654    7.9266    1.7178   17.7214    1.4608    1.6763
   15.5594    0.6127  757.9703   19.2585    1.6098   16.8577    1.5594   21.9223    1.5198    1.5936

  Columns 21 through 30

    1.5105    2.9260    6.7129  143.3904    1.2720   24.3441         0    2.6953    2.4833   12.1299
    1.2397    3.1428   14.7719  139.8654    1.9969   25.5780         0    3.5531    4.2551   14.2384

  Columns 31 through 40

    9.8493   18.1031    9.8473    8.9053    8.9053    0.1775    3.0382    2.7287    6.4866   11.3383
   16.5075   22.3266   16.5035   15.4643   15.4627    0.2793    9.3669    9.2648    7.6838   24.2890

  Columns 41 through 50

    6.0433    6.1148    3.6690    8.5015    8.5015   16.1583   28.4501    8.8576    1.1666    7.6255
    8.1588    8.9740    2.9496   10.5660   10.5660   21.3916   30.9977   16.3218    3.5074    8.4407

  Columns 51 through 60
```

```
    1.6412    7.3209    7.2612    0.9018    7.3762    2.4213    7.2380    2.0823    8.2132       NaN
    4.6252    7.8912   10.8325    3.7838    7.8949    3.0899    7.8009    3.0219   10.2345       NaN

>> hl

hl =

  Columns 1 through 18

     0     0     1     0     0     0     0     0     0     0     0     0     1     0     0     0     0     0

  Columns 19 through 36

     0     0     0     0     0     0     0     0   NaN     0     0     1     1     0     0     1     0     1

  Columns 37 through 54

     1     1     1     0     0     0     0     0     0     1     0     0     1     0     1     0     0     1

  Columns 55 through 60

     0     1     0     1     0   NaN

>> pl

pl =

  Columns 1 through 10

    0.5379    0.8427    0.0392    0.1573    0.9514    0.9660    0.2275    0.5000    0.9199    0.3164

  Columns 11 through 20

    0.2382    0.1213    0.0359    0.3560    0.3113    0.0647    0.1946    0.1024    0.3901    0.1673

  Columns 21 through 30

    0.1681    0.5109    0.1252    0.2195    0.1731    0.9586       NaN    0.0547    0.0505    0.0289

  Columns 31 through 40

    0.0012    0.4896    0.9988    0.0009    0.9991    0.0029    0.0001    0.0001    0.0243    0.9997

  Columns 41 through 50

    0.8501    0.1082    0.7305    0.0818    0.9184    0.0432    0.1034    0.1774    0.0012    0.9234

  Columns 51 through 60
```

```
    0.0089    0.8955    0.7288    0.0003    0.5679    0.0144    0.9838    0.0241    0.7309       NaN

>> cil

cil =

  Columns 1 through 10

     -Inf      -Inf      -Inf      -Inf      -Inf      -Inf      -Inf      -Inf      -Inf      -Inf
    0.1023    9.9166   -2.6030    2.4055    0.8576    7.0416    0.2354    0.3147    0.6639   16.3234

  Columns 11 through 20

     -Inf      -Inf      -Inf      -Inf      -Inf      -Inf      -Inf      -Inf      -Inf      -Inf
    2.3381    0.0370  -15.0060    4.2982    0.3273    0.2876    0.2166    1.2700    0.3419    0.1874

  Columns 21 through 30

     -Inf      -Inf      -Inf      -Inf      -Inf      -Inf      -Inf      -Inf      -Inf      -Inf
    0.1592    0.8518    0.9639   20.7649    0.1986   14.1595       NaN    0.0234    0.0031   -0.5664

  Columns 31 through 40

     -Inf      -Inf      -Inf      -Inf      -Inf      -Inf      -Inf      -Inf      -Inf      -Inf
   -3.2548    5.5212   10.7638   -3.2525   10.2250   -0.0451   -2.4536   -2.4956   -0.3945   16.3875

  Columns 41 through 50

     -Inf      -Inf      -Inf      -Inf      -Inf      -Inf      -Inf      -Inf      -Inf      -Inf
    3.2280    0.5304    1.2605    0.4080    4.8872   -0.2235    1.9211    1.5972   -0.6438    4.1447

  Columns 51 through 60

     -Inf      -Inf      -Inf      -Inf      -Inf      -Inf      -Inf      -Inf      -Inf      -Inf
   -0.4358    3.7018    3.4859   -0.8870    2.3263   -0.2560    4.7876   -0.1458    3.5154       NaN

>> statsl.tstat

ans =

  Columns 1 through 10

    0.0954    1.0092   -1.7746   -1.0092    1.6708    1.8395   -0.7491         0    1.4126   -0.4788

  Columns 11 through 20

   -0.7139   -1.1764   -1.8238   -0.3698   -0.4933   -1.5291   -0.8636   -1.2739   -0.2795   -0.9681

  Columns 21 through 30
```

```
   -0.9650    0.0274   -1.1563   -0.7760   -0.9458    1.7469       NaN   -1.6120   -1.6529   -1.9129

  Columns 31 through 40

   -3.0949   -0.0262    3.0944   -3.2050    3.2039   -2.8132   -3.7771   -3.8559   -1.9889    3.5274

  Columns 41 through 50

    1.0412   -1.2426    0.6158   -1.4008    1.4017   -1.7272   -1.2683   -0.9291   -3.1405    1.4359

  Columns 51 through 60

   -2.4157    1.2624    0.6109   -3.5993    0.1712   -2.2096    2.1618   -1.9942    0.6170       NaN

>> statsl.df

ans =

  Columns 1 through 10

   96.5392  141.9222  120.6220  141.9222  126.7546  136.6986  137.1397  138.3477  132.1104  137.6065

  Columns 11 through 20

  140.8273   86.0743   84.1477  141.1442  140.8087  100.9322  140.6920  136.0255  141.7779  141.6388

  Columns 21 through 30

  136.7940  141.2808   99.1256  141.9121  120.4726  141.6542       NaN  132.3857  114.3362  138.5026

  Columns 31 through 40

  115.8660  136.1832  115.8684  113.4237  113.4309  120.3204   85.7753   83.2255  138.1121  100.5406

  Columns 41 through 50

  130.8821  125.2382  135.7337  135.7801  135.7802  132.1223  140.9682  109.4827   86.5195  140.5598

  Columns 51 through 60

   88.5999  141.2084  124.0862   79.0405  141.3494  134.3209  141.2108  126.0191  135.6402       NaN

>> sd
Undefined function or variable 'sd'.

>> statsl.sd

ans =

  Columns 1 through 10
```

0.4537	22.0643	101.5699	22.0643	1.2496	10.8311	1.4031	1.0436	1.1062	90.3244
0.1957	22.5871	159.1171	22.5871	1.7944	13.2230	1.6976	1.2294	1.4647	75.3982

Columns 11 through 20

14.1988	0.2008	231.6426	20.8214	1.7654	7.9266	1.7178	17.7214	1.4608	1.6763
15.5594	0.6127	757.9703	19.2585	1.6098	16.8577	1.5594	21.9223	1.5198	1.5936

Columns 21 through 30

1.5105	2.9260	6.7129	143.3904	1.2720	24.3441	0	2.6953	2.4833	12.1299
1.2397	3.1428	14.7719	139.8654	1.9969	25.5780	0	3.5531	4.2551	14.2384

Columns 31 through 40

9.8493	18.1031	9.8473	8.9053	8.9053	0.1775	3.0382	2.7287	6.4866	11.3383
16.5075	22.3266	16.5035	15.4643	15.4627	0.2793	9.3669	9.2648	7.6838	24.2890

Columns 41 through 50

6.0433	6.1148	3.6690	8.5015	8.5015	16.1583	28.4501	8.8576	1.1666	7.6255
8.1588	8.9740	2.9496	10.5660	10.5660	21.3916	30.9977	16.3218	3.5074	8.4407

Columns 51 through 60

1.6412	7.3209	7.2612	0.9018	7.3762	2.4213	7.2380	2.0823	8.2132	NaN
4.6252	7.8912	10.8325	3.7838	7.8949	3.0899	7.8009	3.0219	10.2345	NaN

```
>> d

d =

  Columns 1 through 10

    0.0159    0.1682   -0.2958   -0.1682    0.2785    0.3066   -0.1249         0    0.2354   -0.0798

  Columns 11 through 20

   -0.1190   -0.1961   -0.3040   -0.0616   -0.0822   -0.2549   -0.1439   -0.2123   -0.0466   -0.1614

  Columns 21 through 30

   -0.1608    0.0046   -0.1927   -0.1293   -0.1576    0.2911       NaN   -0.2687   -0.2755   -0.3188

  Columns 31 through 40

   -0.5158   -0.0044    0.5157   -0.5342    0.5340   -0.4689   -0.6295   -0.6426   -0.3315    0.5879

  Columns 41 through 50

    0.1735   -0.2071    0.1026   -0.2335    0.2336   -0.2879   -0.2114   -0.1548   -0.5234    0.2393

  Columns 51 through 59

   -0.4026    0.2104    0.1018   -0.5999    0.0285   -0.3683    0.3603   -0.3324    0.1028

>>
```

Young Professionals vs. Old Professionals

Y =

Columns 1 through 10

1.0451   77.5141   269.8521   22.4859   6.4648   37.5887   1.8451   8.4366   6.0000   274.7746

Columns 11 through 20

32.8732   0.4201   319.0437   55.9859   3.3239   17.0873   3.4507   48.7676   2.1268   5.2817

Columns 21 through 30

3.5352   3.8732   28.4817   524.1690   2.4085   49.2704   1.0000   5.9859   8.6944   23.4042

Columns 31 through 40

26.3718   39.4028   73.6296   22.8268   77.1746   0.3803   14.5451   14.2225   8.2845   62.9521

Columns 41 through 50

48.6042   48.2493   3.1521   14.1944   85.8070   14.2789   66.8915   15.9338   3.2775   10.5451

Columns 51 through 60

4.8113   12.5099   7.0817   4.6465   14.1042   3.9394   9.3296   3.9845   9.8338   NaN

>> O

O =

Columns 1 through 10

0.9431   73.9917   312.7681   26.0083   6.5972   36.1611   2.3472   9.0139   6.3750   179.0139

Columns 11 through 20

35.1931   0.3837   343.7833   55.6389   3.2639   17.2389   3.3194   49.0708   1.8611   5.1528

Columns 21 through 30

3.3472   4.2361   27.5778   340.3611   2.8472   45.9764   1.0000   6.8056   6.6833   28.3625

Columns 31 through 40

31.6764   39.9042   68.3236   28.8194   71.1806   0.4792   15.0333   14.7139   13.7764   56.4639

```
  Columns 41 through 50

   47.7389   48.4597    3.7944   15.0194   84.9653   16.4403   68.4153   16.6458    4.5792    9.9958

  Columns 51 through 60

    5.0194    8.8778   10.6056    5.1472   11.7611    5.7806   10.1764    5.8375    5.5597       NaN

>> h

h =

  Columns 1 through 18

     0     0     0     0     0     0     1     1     0     0     0     0     0     0     0     0     0     0

  Columns 19 through 36

     0     0     0     0     0     0     0     0   NaN     0     0     0     0     0     0     0     0     0

  Columns 37 through 54

     0     0     1     0     0     0     0     0     0     0     0     0     0     0     0     1     1     0

  Columns 55 through 60

     0     0     0     0     1   NaN

>> p

p =

  Columns 1 through 10

    0.5897    0.2443    0.0555    0.2443    0.6412    0.4145    0.0295    0.0030    0.0867    0.3416

  Columns 11 through 20

    0.4797    0.5305    0.7688    0.8607    0.7331    0.9313    0.4409    0.9006    0.0780    0.4935

  Columns 21 through 30

    0.1431    0.5385    0.6941    0.3448    0.0610    0.3712       NaN    0.2098    0.3715    0.1942

  Columns 31 through 40

    0.0954    0.8897    0.0953    0.0895    0.0894    0.2832    0.7879    0.7833    0.0345    0.1883

  Columns 41 through 50
```

```
    0.1864    0.7677    0.2134    0.6316    0.6248    0.3387    0.7476    0.7726    0.1462    0.7029

  Columns 51 through 60

    0.8652    0.0360    0.0230    0.6382    0.1763    0.0860    0.5686    0.0918    0.0002       NaN

>> ci

ci =

  Columns 1 through 10

   -0.2725   -2.4339  -86.8487   -9.4787   -0.6934   -2.0212   -0.9536   -0.9554   -0.8048 -103.5253
    0.4766    9.4787    1.0168    2.4339    0.4285    4.8764   -0.0507   -0.1992    0.0548  295.0468

  Columns 11 through 20

   -8.7914   -0.0781 -190.7883   -3.5545   -0.2874   -3.6197   -0.2045   -5.0960   -0.0302   -0.2423
    4.1518    0.1509  141.3090    4.2486    0.4076    3.3166    0.4670    4.4895    0.5615    0.5002

  Columns 21 through 30

   -0.0644   -1.5263   -3.6342 -201.2604   -0.8980   -3.9682       NaN   -2.1059   -2.4401  -12.4723
    0.4404    0.8006    5.4420  568.8762    0.0205   10.5562       NaN    0.4666    6.4621    2.5558

  Columns 31 through 40

  -11.5516   -7.6333   -0.9413  -12.9228   -0.9358   -0.2806   -4.0687   -4.0171  -10.5746   -3.2143
    0.9424    6.6306   11.5532    0.9374   12.9239    0.0828    3.0922    3.0344   -0.4092   16.1907

  Columns 41 through 50

   -0.4229   -1.6158   -1.6590   -4.2192   -2.5536   -6.6122  -10.8672   -5.5751   -3.0631   -2.2917
    2.1536    1.1950    0.3743    2.5690    4.2371    2.2894    7.8197    4.1511    0.4597    3.3902

  Columns 51 through 60

   -2.6290    0.2426   -6.5532   -2.6031   -1.0660   -3.9465   -3.7781   -4.0118    2.0692       NaN
    2.2126    7.0216   -0.4945    1.6016    5.7522    0.2642    2.0844    0.3058    6.4790       NaN

>> stats.tstat

ans =

  Columns 1 through 10

    0.5411    1.1692   -1.9313   -1.1692   -0.4670    0.8184   -2.2021   -3.0182   -1.7255    0.9570

  Columns 11 through 20
```

```
  -0.7087    0.6291   -0.2945    0.1759    0.3417   -0.0864    0.7729   -0.1251    1.7757    0.6865

  Columns 21 through 30

   1.4728   -0.6167    0.3942    0.9507   -1.8889    0.8973       NaN   -1.2600    0.8985   -1.3046

  Columns 31 through 40

  -1.6796   -0.1390    1.6799   -1.7109    1.7113   -1.0784   -0.2696   -0.2755   -2.1429    1.3223

  Columns 41 through 50

   1.3279   -0.2960   -1.2507   -0.4806    0.4901   -0.9601   -0.3224   -0.2896   -1.4617    0.3822

  Columns 51 through 60

  -0.1700    2.1255   -2.3008   -0.4713    1.3594   -1.7299   -0.5715   -1.6988    3.8385       NaN

>> stats.df

ans =

  Columns 1 through 10

   89.5567  140.0054  139.5649  140.0054  130.4820  140.9731  121.9909  140.9892  134.1421   76.0069

  Columns 11 through 20

  140.5193  120.3337  140.9618  138.9772  139.7275  140.6764  139.8811  138.8351  134.0762  140.7933

  Columns 21 through 30

  138.4787  138.6310  124.6238   75.9916  139.0266  131.4480       NaN  137.1971   83.9158  140.3779

  Columns 31 through 40

  133.0732  140.7312  133.0795  129.3435  129.3371  109.6405  140.9657  140.8825  103.1063  137.9732

  Columns 41 through 50

  140.9296  140.9582  122.7641  140.6904  140.6798  139.9575  140.7582  135.4588  133.7094  140.3960

  Columns 51 through 60

  137.7128  101.5465  133.5481  128.4206  133.7326  131.9019  130.0433  124.8240  118.4207       NaN

>> stats.sd

ans =
```

```
   1.0e+03 *

  Columns 1 through 10

    0.0015    0.0171    0.1250    0.0171    0.0014    0.0103    0.0011    0.0011    0.0011    0.8256
    0.0006    0.0189    0.1404    0.0189    0.0019    0.0106    0.0016    0.0012    0.0014    0.1723

  Columns 11 through 20

    0.0189    0.0004    0.4945    0.0124    0.0011    0.0102    0.0011    0.0153    0.0010    0.0011
    0.0203    0.0003    0.5099    0.0111    0.0010    0.0108    0.0010    0.0137    0.0008    0.0011

  Columns 21 through 30

    0.0008    0.0033    0.0159    1.5953    0.0013    0.0246         0    0.0035    0.0180    0.0218
    0.0007    0.0038    0.0110    0.3326    0.0015    0.0189         0    0.0042    0.0057    0.0236

  Columns 31 through 40

    0.0163    0.0209    0.0163    0.0174    0.0174    0.0004    0.0107    0.0104    0.0095    0.0269
    0.0212    0.0222    0.0212    0.0240    0.0240    0.0007    0.0110    0.0109    0.0195    0.0316

  Columns 41 through 50

    0.0038    0.0042    0.0024    0.0099    0.0099    0.0128    0.0286    0.0160    0.0046    0.0082
    0.0040    0.0043    0.0036    0.0106    0.0106    0.0141    0.0279    0.0132    0.0060    0.0089

  Columns 51 through 60

    0.0078    0.0129    0.0079    0.0052    0.0114    0.0054    0.0074    0.0052    0.0079       NaN
    0.0068    0.0064    0.0102    0.0073    0.0091    0.0072    0.0101    0.0077    0.0050       NaN

>> hr

hr =

  Columns 1 through 18

     0     0     0     0     0     0     0     0     0     0     0     0     0     0     0     0     0     0

  Columns 19 through 36

     1     0     0     0     0     0     0     0   NaN     0     0     0     0     0     1     0     1     0

  Columns 37 through 54

     0     0     0     0     0     0     0     0     0     0     0     0     0     0     0     1     0     0

  Columns 55 through 60
```

```
     0     0     0     0     1   NaN

>> pr

pr =

  Columns 1 through 10

    0.2949    0.1222    0.9723    0.8778    0.6794    0.2073    0.9852    0.9985    0.9566    0.1708

  Columns 11 through 20

    0.7601    0.2652    0.6156    0.4303    0.3666    0.5344    0.2205    0.5497    0.0390    0.2468

  Columns 21 through 30

    0.0715    0.7308    0.3470    0.1724    0.9695    0.1856       NaN    0.8951    0.1858    0.9029

  Columns 31 through 40

    0.9523    0.5552    0.0477    0.9552    0.0447    0.8584    0.6061    0.6083    0.9828    0.0941

  Columns 41 through 50

    0.0932    0.6162    0.8933    0.6842    0.3124    0.8307    0.6262    0.6137    0.9269    0.3514

  Columns 51 through 60

    0.5674    0.0180    0.9885    0.6809    0.0882    0.9570    0.7157    0.9541    0.0001       NaN

>> cir

cir =

  Columns 1 through 10

   -0.2113   -1.4661  -79.7100   -8.5109   -0.6022   -1.4608   -0.8801   -0.8940   -0.7350  -70.8538
       Inf       Inf       Inf       Inf       Inf       Inf       Inf       Inf       Inf       Inf

  Columns 11 through 20

   -7.7399   -0.0595 -163.8097   -2.9205   -0.2310   -3.0562   -0.1500   -4.3171    0.0179   -0.1820
       Inf       Inf       Inf       Inf       Inf       Inf       Inf       Inf       Inf       Inf

  Columns 21 through 30

   -0.0234   -1.3373   -2.8958 -138.1309   -0.8234   -2.7874       NaN   -1.8969   -1.7117  -11.2514
       Inf       Inf       Inf       Inf       Inf       Inf       Inf       Inf       Inf       Inf

  Columns 31 through 40
```

```
  -10.5359   -6.4745    0.0744  -11.7957    0.1912   -0.2510   -3.4870   -3.4443   -9.7456   -1.6375
       Inf       Inf       Inf       Inf       Inf       Inf       Inf       Inf       Inf       Inf

  Columns 41 through 50

   -0.2136   -1.3875   -1.4935   -3.6677   -2.0019   -5.8890   -9.3491   -4.7847   -2.7767   -1.8301
       Inf       Inf       Inf       Inf       Inf       Inf       Inf       Inf       Inf       Inf

  Columns 51 through 60

   -2.2356    0.7955   -6.0607   -2.2612   -0.5118   -3.6041   -3.3014   -3.6605    2.4281       NaN
       Inf       Inf       Inf       Inf       Inf       Inf       Inf       Inf       Inf       Inf

>> statsr.tstat

ans =

  Columns 1 through 10

    0.5411    1.1692   -1.9313   -1.1692   -0.4670    0.8184   -2.2021   -3.0182   -1.7255    0.9570

  Columns 11 through 20

   -0.7087    0.6291   -0.2945    0.1759    0.3417   -0.0864    0.7729   -0.1251    1.7757    0.6865

  Columns 21 through 30

    1.4728   -0.6167    0.3942    0.9507   -1.8889    0.8973       NaN   -1.2600    0.8985   -1.3046

  Columns 31 through 40

   -1.6796   -0.1390    1.6799   -1.7109    1.7113   -1.0784   -0.2696   -0.2755   -2.1429    1.3223

  Columns 41 through 50

    1.3279   -0.2960   -1.2507   -0.4806    0.4901   -0.9601   -0.3224   -0.2896   -1.4617    0.3822

  Columns 51 through 60

   -0.1700    2.1255   -2.3008   -0.4713    1.3594   -1.7299   -0.5715   -1.6988    3.8385       NaN

>> statsr.df

ans =

  Columns 1 through 10

   89.5567  140.0054  139.5649  140.0054  130.4820  140.9731  121.9909  140.9892  134.1421   76.0069
```

```
  Columns 11 through 20

  140.5193  120.3337  140.9618  138.9772  139.7275  140.6764  139.8811  138.8351  134.0762  140.7933

  Columns 21 through 30

  138.4787  138.6310  124.6238   75.9916  139.0266  131.4480       NaN  137.1971   83.9158  140.3779

  Columns 31 through 40

  133.0732  140.7312  133.0795  129.3435  129.3371  109.6405  140.9657  140.8825  103.1063  137.9732

  Columns 41 through 50

  140.9296  140.9582  122.7641  140.6904  140.6798  139.9575  140.7582  135.4588  133.7094  140.3960

  Columns 51 through 60

  137.7128  101.5465  133.5481  128.4206  133.7326  131.9019  130.0433  124.8240  118.4207       NaN

>> statsr.sd

ans =

   1.0e+03 *

  Columns 1 through 10

    0.0015    0.0171    0.1250    0.0171    0.0014    0.0103    0.0011    0.0011    0.0011    0.8256
    0.0006    0.0189    0.1404    0.0189    0.0019    0.0106    0.0016    0.0012    0.0014    0.1723

  Columns 11 through 20

    0.0189    0.0004    0.4945    0.0124    0.0011    0.0102    0.0011    0.0153    0.0010    0.0011
    0.0203    0.0003    0.5099    0.0111    0.0010    0.0108    0.0010    0.0137    0.0008    0.0011

  Columns 21 through 30

    0.0008    0.0033    0.0159    1.5953    0.0013    0.0246         0    0.0035    0.0180    0.0218
    0.0007    0.0038    0.0110    0.3326    0.0015    0.0189         0    0.0042    0.0057    0.0236

  Columns 31 through 40

    0.0163    0.0209    0.0163    0.0174    0.0174    0.0004    0.0107    0.0104    0.0095    0.0269
    0.0212    0.0222    0.0212    0.0240    0.0240    0.0007    0.0110    0.0109    0.0195    0.0316

  Columns 41 through 50

    0.0038    0.0042    0.0024    0.0099    0.0099    0.0128    0.0286    0.0160    0.0046    0.0082
    0.0040    0.0043    0.0036    0.0106    0.0106    0.0141    0.0279    0.0132    0.0060    0.0089
```

```
  Columns 51 through 60

    0.0078    0.0129    0.0079    0.0052    0.0114    0.0054    0.0074    0.0052    0.0079       NaN
    0.0068    0.0064    0.0102    0.0073    0.0091    0.0072    0.0101    0.0077    0.0050       NaN

>> hl

hl =

  Columns 1 through 18

     0     0     1     0     0     0     1     1     1     0     0     0     0     0     0     0     0     0

  Columns 19 through 36

     0     0     0     0     0     0     1     0   NaN     0     0     0     1     0     0     1     0     0

  Columns 37 through 54

     0     0     1     0     0     0     0     0     0     0     0     0     0     0     0     0     1     0

  Columns 55 through 60

     0     1     0     1     0   NaN

>> pl

pl =

  Columns 1 through 10

    0.7051    0.8778    0.0277    0.1222    0.3206    0.7927    0.0148    0.0015    0.0434    0.8292

  Columns 11 through 20

    0.2399    0.7348    0.3844    0.5697    0.6334    0.4656    0.7795    0.4503    0.9610    0.7532

  Columns 21 through 30

    0.9285    0.2692    0.6530    0.8276    0.0305    0.8144       NaN    0.1049    0.8142    0.0971

  Columns 31 through 40

    0.0477    0.4448    0.9523    0.0448    0.9553    0.1416    0.3939    0.3917    0.0172    0.9059

  Columns 41 through 50

    0.9068    0.3838    0.1067    0.3158    0.6876    0.1693    0.3738    0.3863    0.0731    0.6486
```

```
  Columns 51 through 60

    0.4326    0.9820    0.0115    0.3191    0.9118    0.0430    0.2843    0.0459    0.9999       NaN

>> cil

cil =

  Columns 1 through 10

      -Inf      -Inf      -Inf      -Inf      -Inf      -Inf      -Inf      -Inf      -Inf      -Inf
    0.4153    8.5109   -6.1219    1.4661    0.3373    4.3161   -0.1242   -0.2606   -0.0150  262.3753

  Columns 11 through 20

      -Inf      -Inf      -Inf      -Inf      -Inf      -Inf      -Inf      -Inf      -Inf      -Inf
    3.1003    0.1322  114.3304    3.6146    0.3511    2.7531    0.4125    3.7107    0.5134    0.4398

  Columns 21 through 30

      -Inf      -Inf      -Inf      -Inf      -Inf      -Inf      -Inf      -Inf      -Inf      -Inf
    0.3994    0.6115    4.7036  505.7467   -0.0541    9.3754       NaN    0.2576    5.7337    1.3349

  Columns 31 through 40

      -Inf      -Inf      -Inf      -Inf      -Inf      -Inf      -Inf      -Inf      -Inf      -Inf
   -0.0732    5.4718   10.5376   -0.1896   11.7969    0.0532    2.5105    2.4616   -1.2382   14.6139

  Columns 41 through 50

      -Inf      -Inf      -Inf      -Inf      -Inf      -Inf      -Inf      -Inf      -Inf      -Inf
    1.9443    0.9666    0.2089    2.0176    3.6854    1.5662    6.3016    3.3606    0.1733    2.9286

  Columns 51 through 60

      -Inf      -Inf      -Inf      -Inf      -Inf      -Inf      -Inf      -Inf      -Inf      -Inf
    1.8192    6.4686   -0.9870    1.2597    5.1980   -0.0781    1.6078   -0.0455    6.1200       NaN

>> statsl.tstat

ans =

  Columns 1 through 10

    0.5411    1.1692   -1.9313   -1.1692   -0.4670    0.8184   -2.2021   -3.0182   -1.7255    0.9570

  Columns 11 through 20

   -0.7087    0.6291   -0.2945    0.1759    0.3417   -0.0864    0.7729   -0.1251    1.7757    0.6865
```

```
  Columns 21 through 30

    1.4728   -0.6167    0.3942    0.9507   -1.8889    0.8973       NaN   -1.2600    0.8985   -1.3046

  Columns 31 through 40

   -1.6796   -0.1390    1.6799   -1.7109    1.7113   -1.0784   -0.2696   -0.2755   -2.1429    1.3223

  Columns 41 through 50

    1.3279   -0.2960   -1.2507   -0.4806    0.4901   -0.9601   -0.3224   -0.2896   -1.4617    0.3822

  Columns 51 through 60

   -0.1700    2.1255   -2.3008   -0.4713    1.3594   -1.7299   -0.5715   -1.6988    3.8385       NaN

>> statsl.df

ans =

  Columns 1 through 10

   89.5567  140.0054  139.5649  140.0054  130.4820  140.9731  121.9909  140.9892  134.1421   76.0069

  Columns 11 through 20

  140.5193  120.3337  140.9618  138.9772  139.7275  140.6764  139.8811  138.8351  134.0762  140.7933

  Columns 21 through 30

  138.4787  138.6310  124.6238   75.9916  139.0266  131.4480       NaN  137.1971   83.9158  140.3779

  Columns 31 through 40

  133.0732  140.7312  133.0795  129.3435  129.3371  109.6405  140.9657  140.8825  103.1063  137.9732

  Columns 41 through 50

  140.9296  140.9582  122.7641  140.6904  140.6798  139.9575  140.7582  135.4588  133.7094  140.3960

  Columns 51 through 60

  137.7128  101.5465  133.5481  128.4206  133.7326  131.9019  130.0433  124.8240  118.4207       NaN

>> sd
Undefined function or variable 'sd'.

>> statsl.sd

ans =
```

1.0e+03 *

Columns 1 through 10

0.0015	0.0171	0.1250	0.0171	0.0014	0.0103	0.0011	0.0011	0.0011	0.8256
0.0006	0.0189	0.1404	0.0189	0.0019	0.0106	0.0016	0.0012	0.0014	0.1723

Columns 11 through 20

0.0189	0.0004	0.4945	0.0124	0.0011	0.0102	0.0011	0.0153	0.0010	0.0011
0.0203	0.0003	0.5099	0.0111	0.0010	0.0108	0.0010	0.0137	0.0008	0.0011

Columns 21 through 30

0.0008	0.0033	0.0159	1.5953	0.0013	0.0246	0	0.0035	0.0180	0.0218
0.0007	0.0038	0.0110	0.3326	0.0015	0.0189	0	0.0042	0.0057	0.0236

Columns 31 through 40

0.0163	0.0209	0.0163	0.0174	0.0174	0.0004	0.0107	0.0104	0.0095	0.0269
0.0212	0.0222	0.0212	0.0240	0.0240	0.0007	0.0110	0.0109	0.0195	0.0316

Columns 41 through 50

0.0038	0.0042	0.0024	0.0099	0.0099	0.0128	0.0286	0.0160	0.0046	0.0082
0.0040	0.0043	0.0036	0.0106	0.0106	0.0141	0.0279	0.0132	0.0060	0.0089

Columns 51 through 60

0.0078	0.0129	0.0079	0.0052	0.0114	0.0054	0.0074	0.0052	0.0079	NaN
0.0068	0.0064	0.0102	0.0073	0.0091	0.0072	0.0101	0.0077	0.0050	NaN

```
>> d

d =

  Columns 1 through 10

    0.0910    0.1954   -0.3228   -0.1954   -0.0780    0.1368   -0.3672   -0.5047   -0.2881    0.1611

  Columns 11 through 20

   -0.1185    0.1055   -0.0493    0.0294    0.0572   -0.0144    0.1293   -0.0209    0.2974    0.1148

  Columns 21 through 30

    0.2465   -0.1030    0.0661    0.1600   -0.3156    0.1503       NaN   -0.2105    0.1511   -0.2181

  Columns 31 through 40

   -0.2804   -0.0232    0.2805   -0.2855    0.2856   -0.1797   -0.0451   -0.0461   -0.3568    0.2209

  Columns 41 through 50

    0.2220   -0.0495   -0.2086   -0.0803    0.0819   -0.1605   -0.0539   -0.0485   -0.2440    0.0639

  Columns 51 through 59

   -0.0285    0.3570   -0.3841   -0.0786    0.2277   -0.2888   -0.0954   -0.2834    0.6439

>>
```
